# Detection of endometrial cancer in cervico-vaginal fluid and blood plasma: leveraging proteomics and machine learning for biomarker discovery

**DOI:** 10.1016/j.ebiom.2024.105064

**Published:** 2024-03-20

**Authors:** Kelechi Njoku, Andrew Pierce, Davide Chiasserini, Bethany Geary, Amy E. Campbell, Janet Kelsall, Rachel Reed, Nophar Geifman, Anthony D. Whetton, Emma J. Crosbie

**Affiliations:** a Division of Cancer Sciences, University of Manchester, School of Medical Sciences, Faculty of Biology, Medicine and Health, 5th Floor Research, St Mary's Hospital, Road, Manchester, M13 9WL, UK; b Stoller Biomarker Discovery Centre, Institute of Cancer Sciences, Faculty of Biology, Medicine and Health, University of Manchester, Manchester, UK; c Department of Clinical Oncology, Christie NHS Foundation Trust, Manchester, UK; d North Wales Medical School, Bangor University, Bangor, Gwynedd, LL57 2DG, UK; e Department of Medicine and Surgery, Section of Physiology and Biochemistry, University of Perugia, 06132, Perugia, Italy; f Medical Research Council Protein Phosphorylation and Ubiquitylation Unit, School of Life Sciences, University of Dundee, Dow Street, Dundee, DD1 5EH, UK; g School of Health Sciences, Faculty of Health and Medical Sciences, University of Surrey, GU2 7XH, UK; h Veterinary Health Innovation Engine, School of Veterinary Medicine, Faculty of Health and Medical Sciences, University of Surrey, GU2 7XH, UK

**Keywords:** Endometrial cancer, Cervico-vaginal fluid, Plasma, Proteins, Biomarker

## Abstract

**Background:**

The anatomical continuity between the uterine cavity and the lower genital tract allows for the exploitation of uterine-derived biomaterial in cervico-vaginal fluid for endometrial cancer detection based on non-invasive sampling methodologies. Plasma is an attractive biofluid for cancer detection due to its simplicity and ease of collection. In this biomarker discovery study, we aimed to identify proteomic signatures that accurately discriminate endometrial cancer from controls in cervico-vaginal fluid and blood plasma.

**Methods:**

Blood plasma and Delphi Screener-collected cervico-vaginal fluid samples were acquired from symptomatic post-menopausal women with (n = 53) and without (n = 65) endometrial cancer. Digitised proteomic maps were derived for each sample using sequential window acquisition of all theoretical mass spectra (SWATH-MS). Machine learning was employed to identify the most discriminatory proteins. The best diagnostic model was determined based on accuracy and model parsimony.

**Findings:**

A protein signature derived from cervico-vaginal fluid more accurately discriminated cancer from control samples than one derived from plasma. A 5-biomarker panel of cervico-vaginal fluid derived proteins (HPT, LG3BP, FGA, LY6D and IGHM) predicted endometrial cancer with an AUC of 0.95 (0.91–0.98), sensitivity of 91% (83%–98%), and specificity of 86% (78%–95%). By contrast, a 3-marker panel of plasma proteins (APOD, PSMA7 and HPT) predicted endometrial cancer with an AUC of 0.87 (0.81–0.93), sensitivity of 75% (64%–86%), and specificity of 84% (75%–93%). The parsimonious model AUC values for detection of stage I endometrial cancer in cervico-vaginal fluid and blood plasma were 0.92 (0.87–0.97) and 0.88 (0.82–0.95) respectively.

**Interpretation:**

Here, we leveraged the natural shed of endometrial tumours to potentially develop an innovative approach to endometrial cancer detection. We show proof of principle that endometrial cancers secrete unique protein signatures that can enable cancer detection via cervico-vaginal fluid assays. Confirmation in a larger independent cohort is warranted.

**Funding:**

10.13039/501100000289Cancer Research UK, 10.13039/501100015570Blood Cancer UK, 10.13039/501100000272National Institute for Health Research.

**Research in context**

**Evidence before this study**

Endometrial cancer is the most common gynaecological malignancy in high-income countries and its incidence is rising. Most women present following the onset of post-menopausal bleeding, a red-flag symptom that triggers urgent investigations by sequential transvaginal ultrasound scan, outpatient hysteroscopy and endometrial biopsy. These diagnostic tests are invasive, anxiety-provoking and avoidable as only 5–10% of symptomatic women have a sinister underlying pathology. A simple, non-invasive and cost-effective detection tool that accurately identifies women with endometrial cancer, whilst safely reassuring the vast majority of women who do not have cancer could transform patient care. Plasma is an attractive biofluid for cancer detection due to its simplicity and ease of collection but may be limited by the low yield of cancer related signals in blood, especially in small and early-stage cancers. There is growing evidence that endometrial cancers secrete cancer-related biomolecules through the cervix into the lower genital tract where they can be collected using non-invasive sampling methodologies.

**Added value of this study**

In this study, we leverage the natural shed of endometrial tumours to identify a set of high-performing protein signatures that can enable the detection of endometrial cancer in cervico-vaginal fluid. We show proof-of-principle that endometrial cancers secrete unique proteins that can be assayed in samples collected from the vagina by gentle lavage using the Delphi screener. The identified biomarker signatures showed excellent accuracy (AUC 0.95) to warrant clinical translation and have mechanistic links with the malignant transformation process.

**Implications of all the available evidence**

Protein signatures assayed in vaginal fluid samples collected by gentle lavage using the Delphi screener may provide a safe, acceptable and economic detection tool in symptomatic post-menopausal women. We envisage that the translation of the biomarker signatures to clinical practice is contingent on the development of clinically tractable assays based on ELISA, Lumipulse ® technology, or even lateral flow test technology for point of care testing. A validated cervico-vaginal fluid multi-protein biomarker assay using any of these platforms can lead to a rapid and efficient triage of symptomatic women with suspected endometrial cancer, with substantial cost-saving implications for health service providers, whilst saving the vast majority of healthy women from invasive tests that are distressing and avoidable. Confirmatory studies based on immunoassays or targeted proteomics in a larger study cohort are now planned to validate these biomarker signatures and to elucidate their role in endometrial tumorigenesis.

## Introduction

Endometrial cancer is the most common gynaecological malignancy in high-income countries, with over 400,000 incident cases and 97,000 deaths reported globally in 2020.^1^ Its incidence is rising, year-on-year, in tandem with the rising prevalence of obesity.^2^ In 2021, there were an estimated 66,570 new cases in the United States of America and 12,940 succumbed to their disease.^3^ When diagnosed early, endometrial cancer is amenable to curative hysterectomy, with over 90% surviving for at least five years following treatment. By contrast, those with advanced or metastatic disease have poor outcomes, with five-year survival estimate of about 15%. Early detection is therefore crucial to good survival outcomes.^1^^,^^2^

Over 90% of women with endometrial cancer present with postmenopausal bleeding, a red flag symptom that triggers urgent investigation by sequential transvaginal ultrasound scan (TVS), outpatient hysteroscopy and endometrial biopsy. These investigations can be painful and anxiety-provoking, and for most, they are avoidable as only 5–10% of symptomatic women have an underlying malignancy. At the forefront of the priorities of patients, clinicians and the general public is the development of simple, non-invasive and cost-effective tests for cancer early detection.^4^ Novel approaches to the detection of endometrial cancer based on minimally-invasive sampling methodologies and measurement of specific biomarkers are urgently needed to reduce the burden of the disease on patients and health service providers.^5^

Blood sampling is easily accessible and acceptable to patients, qualities that make it attractive for cancer biomarker discovery and validation. However, the potential for blood-based biomarkers to enable cancer detection may be limited by the low yield of cancer-derived signals in blood, especially in small and early-stage tumours.^6^ The anatomical continuity between the uterine cavity and the lower genital tract allows for the exploitation of uterine derived biomaterial for biomarker discovery.^7^ Cervico-vaginal fluid, a complex mixture of uterine, cervical and vaginal secretions, has previously been explored as a source of biomarkers for pregnancy related pathologies, infective/inflammatory conditions of the lower genital tract and cervical neoplasia.^8^ Building on this work, O'Flynn and colleagues showed that endometrial cancer can be detected in cervico-vaginal fluid with good diagnostic accuracy based on cytology.^9^ In their cross-sectional study of 216 women with or at risk of endometrial cancer, cervico-vaginal fluid cytology demonstrated a sensitivity of 89.6% and 88.7% specificity for endometrial cancer detection.^9^ Whilst potentially of clinical value, cytology is labour intensive with only moderate inter-observer reproducibility.^10^ Its translational potential is limited by the need for highly trained cytopathology specialists.^9^

Advances in high-throughput technologies and data analysis using artificial intelligence have stimulated a renewed interest in cancer biomarker discovery.^11^ Proteomics, for example, allows for comprehensive measurement of thousands of proteins simultaneously in biological samples, enabling the identification of biomarkers for cancer detection.^5^ Sequential window acquisition of all theoretical mass-spectra (SWATH-MS) is a proteomic profiling platform with high reproducibility, precision, and accuracy.^12^ In this study, cervico-vaginal fluid protein signatures from both supernatant and cell-pellet fractions for the detection of endometrial cancer were generated from SWATH-MS analyses. We compared the diagnostic performance of these signatures to those derived from matched plasma samples and explored the potential of the models to not only detect early-stage tumours but also locally advanced/metastatic disease and biologically aggressive endometrial cancer phenotypes.

## Methods

### Research ethics, approvals and patient involvement

This study received ethical approval by the North-West Greater Manchester Research Ethics Committee (reference-16/NW/0660). All study participants gave written informed consent for their clinical data and biological samples to be used for research. The study was conducted in line with Good Clinical Practice guidelines and in accordance with the Helsinki declarations and the Human Tissue Act of 2004. The study question was developed in partnership with patients, carers and physician groups in the James Lind Alliance (JLA) Detecting Cancer Early Priority Setting Partnership (Question#1: “What simple, non-invasive, painless, cost-effective, and convenient tests can be used to detect cancer early?”).^4^ The study also addresses the 2nd most important research question of the JLA Womb Cancer Priority Setting Partnership (Question #2: “Which women with abnormal uterine bleeding should be referred for specialist review?”).^13^

### Study participants

We recruited women referred with postmenopausal bleeding as well as those with known endometrial cancer attending the Gynaecology Outpatient Departments at St Marys Hospital, Manchester University NHS Foundation Trust and the Royal Oldham Hospital of the Northern Care Alliance NHS Group, between April 2019 and March 2020. Cases consisted of women with histo-pathological evidence of endometrial cancer based on hysterectomy specimens, assessed by at least two specialist gynaecological pathologists reporting to the Royal College of Pathology Standards. Controls were symptomatic women with no evidence of endometrial cancer or atypical hyperplasia, following routine diagnostic investigations that included TVS, endometrial biopsy and/or hysteroscopy. Women with benign pathologies such as atrophic vaginitis and benign polyps were eligible to serve as controls. All samples were acquired prior to commencement of treatment, including surgery, hormone therapy or chemotherapy. Women who had previously had a hysterectomy and those with a previous history of gynaecological malignancy were excluded.

### Research sample and clinical data collection

Blood and cervico-vaginal fluid samples were acquired from each study participant at the same time point (Figure S1). Blood samples were collected in standard EDTA tubes, centrifuged at 1500 g for 15 min at room temperature and the supernatant (plasma) stored at −80 °C pending further analysis. Cervico-vaginal fluid was collected using the Delphi screener (Rovers, Netherlands), a sterile plastic, syringe-like device approximately 20 cm in length. Participants were placed in a supine position with legs apart, knees bent and heels brought up to the bottom. The device was then inserted into the posterior fornix of the vagina and the reservoir of saline expelled. The fluid was re-aspirated by taking the pressure off the plunger and the device slowly rotated and retracted. Following collection, cervico-vaginal samples were centrifuged at 1000 g for 10 min to separate cellular pellets/debris from supernatant fractions. The supernatant fractions were stored at −80 °C pending further analyses while the pellets were treated with 1 ml of red blood cell (RBC) lysis solution (BD CytoRich Red, Becton Dickinson UK), re-suspended by gentle pipetting, incubated for 5 min at room temperature and centrifuged at 1000 g for 10 min. The RBC lysis supernatant was discarded and the cellular pellets/debris washed by centrifugation at 1000 g for 5 min with phosphate buffered saline prior to storage at −80 °C.

### Plasma and cervico-vaginal fluid sample preparation

Plasma samples were immunodepleted of high abundant proteins using Pierce™ Top 12 Abundant Protein Depletion Spin Columns based on the manufacturer's instructions (ThermoFisher Scientific, Hemel Hempstead). Depleted plasma samples and cervicovaginal fluid samples were purified and concentrated using Amicon® Ultra-0.5 centrifugal filter device (Sigma–Aldrich, Merck KGaA, Darmstadt, Germany) and Agilent spin concentrator (4 mil 30 K MWCO concentrator, Agilent UK), respectively. Using the same spin, buffer exchange with 25 mM ammonium bicarbonate was performed. Cervico-vaginal fluid cellular pellets/debris were lysed in 0.5 M TEAB buffer with 5% (w/v) Deoxycholate (Phosphatase inhibitor (PhosSTOP) and Benzonase) and incubated for 30 min at 4 °C. Lysates were vortexed briefly every 10 min and samples checked until clear of DNA. Samples were centrifuged at 10,000 g at 4 °C degrees for 10 min and supernatants collected in pre-chilled Eppendorf vials. Protein concentration was measured using the Bradford assay (Bio-rad laboratories, Watford, UK). Based on the estimated protein concentrations of the cervico-vaginal fluid supernatant and cellular lysates, appropriate volumes containing 50 μg of protein were transferred into clean Eppendorf vials. Disulphide bonds were reduced with the use of 5 mM of Dithiothreitol and 1% Sodium Deoxycholate and incubation in a heating block at 60° for 30 min. Alkylation was performed using 50 mM iodoacetamide in the dark at room temperature for 30 min and digestion completed with trypsin (Promega, Southampton, UK) at a 10:1 protein: trypsin ratio and incubated overnight at 37 °C. 1% Formic acid was added to the sample for a final concentration of 0.5%. Deoxycholate was then pelleted by centrifugation at 12,000 × *g* for 10 min at 10 °C and the supernatant transferred to fresh microfuge tubes. Samples were then dried using the MiVac Quattro Concentrator for 3 h.

### SWATH-MS data acquisition

Mass spectrometric analysis of the cervico-vaginal fluid samples and plasma was performed using a 6600 TripleTOF (Sciex, Warrington, UK). Liquid chromatography employed a 120-min gradient between a buffer A of 98% Water, 2% (v/v) Acetonitrile and 0.1% (w/v) Formic Acid and a buffer B of 80% Acetonitrile, 20% Water, 0.1% Formic Acid. Dried sample peptides were vigorously re-suspended in a buffer of 4% (v/v) Acetonitrile and 0.1% Formic Acid and injected in duplicate. We used an Eksigent system comprising of a nanoLC 400 autosampler along with a 425 pump module with YMC-Triart C18 trap column and a YMC-Triart C18 analytical column. Mass spectra were collected via data independent acquisition (SWATH-MS) and utilising the 100-variable window with optimised collision energy equations. The resultant spectral data files were converted using wiffconverter (Sciex, Warrington, UK) and searched against the human plasma library (for plasma data files) and our already published bespoke consensus spectral library comprising 19,394 peptides and 2425 cervico-vaginal fluid proteins (for cervico-vaginal fluid data files)^14^ using OpenSwath (version 2.0.0). Peptide matches were assessed using pyProphet (version 0.18.3) within the TransProteomic Pipeline (TPP) and then aligned using the TRIC tool from the OpenSWATH pipeline. Researchers were blinded to relevant clinical data and histopathological findings during sample preparation and mass-spectrometric analyses. Downstream statistical analysis was performed on proteins present in at least 20% of samples using the Bioconductor (release 3.5) packages SWATH2Stats and MSstats within the R language (version 3.4.1). We excluded all potential contaminants and decoy sequences prior to statistical analyses.

### Statistics and data analyses

All data analyses were carried out using R version 4.1.1 (R Development Core Team, Vienna, Austria) and GraphPad Prism 9.3.1. Our power estimation confirmed that a sample size of 100 women, including n = 50 cancer cases and n = 50 controls, is required to identify a (true) endometrial cancer biomarker or biomarker signature with an expected AUC of 0.90 (0.84, 0.96) at a 95% confidence level and power >90%. Data normality was assessed using the Shapiro–Wilk test alongside Q–Q plots. Descriptive analyses were performed using medians (IQR) for continuous data and counts (%) for categorical data. Differences between study groups were assessed using Mann–Whitney U test for continuous variables and the chi-square test for categorical variables. The assumptions underlying the use of these tests were assessed and met. Missing values were replaced by zero values, as these are likely a result of proteins being at low concentrations below the detection limit.^15^ We assessed the log2 fold change of protein concentration between cases and controls and applied a false discovery rate adjustment for multiple testing using the Benjamini–Hochberg correction method. Principal component analysis (PCA) was used for dimensionality reduction and visualisation. Gene ontology analysis was carried by inputting differentially expressed proteins against a background of all identified proteins in the study. Functional enrichment analyses were performed using the clusterprofiler package in R. Feature selection was undertaken by random forest (RF) modelling using the randomForest package in the R statistical language. Random forests (using a random seed set at 1000) from a bootstrap selection (approximately 80% for a randomly selected training set and 20% as test set) were used to perform cross validation. The model was initially tuned to obtain the best *mtry* parameter on the training set. This parameter decides the number of features to be considered at each split of the decision trees. The *mtry* parameter defined on the training set was then used in RF modelling still on the training dataset using a number of trees = 1000. The RF model built on the training set was then used for prediction on the test set, from which the final accuracy metrics were derived. The most discriminatory proteins were ranked according to their contribution to the Mean Decrease in Accuracy plot of the RF algorithm. For AUCs, threshold values, sensitivities, and specificities, 95% CI were calculated by using 2000 bootstrap replicates. Nested logistic regression models of increasing complexity, adjusting for age and BMI as continuous variables, were developed by the sequential incorporation of proteins based on their ranking in the Mean Decrease in Accuracy of the RF algorithm. The linearity assumption underlying logistic regression modelling for quantitative predictors was assessed using scatter plots and met for all models. The parsimonious model, defined as the model which best balances diagnostic accuracy with model simplicity, was identified. We assessed model performance by generating receiver-operator characteristic curves and computing the area under the curve (AUC) and the 95% confidence intervals. Akaike information criteria (AIC) and Likelihood ratio tests were used to compare nested model performances. We adjusted the positive and negative predictive values for disease prevalence using a 9% pooled risk estimate based on the meta-analysis of the prevalence of endometrial cancer in symptomatic post-menopausal women.^16^ To assess the robustness of the biomarkers identified, feature selection using the Boruta algorithm was undertaken and the confirmed proteins compared to those identified by our RF model. The Boruta algorithm compares the performance of each candidate feature in the classification model to that of a randomly created ‘shadow feature’. The Boruta algorithm incorporates data from all collinearly related proteins rather than randomly selecting one among them as is commonly done by other algorithms.

### Role of funders

The funders had no role in the study design, data collection, data analyses, interpretation or writing of the report.

## Results

### Participant demographics

In total, 118 symptomatic post-menopausal women participated in this study, including 53 (45%) with a confirmed diagnosis of endometrial cancer and 65 (55%) with no evidence of cancer (Table 1). Their median age and BMI were 57 years (Interquartile range (IQR) 52, 67) and 28 kg/m^2^ (IQR 24, 34), respectively, and they were mostly of White British ethnicity (86% White, 10% Asian and 4% Afro-Caribbean). Women with endometrial cancer were older (median age 67 years (IQR 58, 73) vs 53 years (IQR 51, 58), p < 0.0001, Mann–Whitney U test) and with higher BMI (median 30.8 kg/m^2^ (IQR 25.3, 37) vs 27 kg/m^2^ (IQR 24, 33), p = 0.048, Mann–Whitney U test), than their control counterparts (Table 1). Most of the women with cancer had low-grade (64% grade I/II), early-stage (77% FIGO stage I) endometrial tumours of endometrioid histological phenotype (79%). Eighteen women (38%) had lympho-vascular space invasion and 21 (45%) had a myometrial depth of ≥50%. A STARD diagram showing the flow of study participants is shown in Figure S2.

**Table 1:** Clinico-pathological characteristics of the study cohort.

Participant characteristics	Total cohort (n = 118)	Cases (n = 53)	Controls (n = 65)	p-value
**Clinical characteristics**				
Age (years) median (IQR)	57 (52–67)	67 (58–73)	53 (51–58)	p < 0.0001^a^
BMI (kg/m^2^) median (IQR)	28.0 (24–34)	30.8 (25.3–37)	27.0 (24–33)	0.048^a^
White ethnicity	102 (86%)	44 (83%)	58 (89%)	0.327^b^
Endometrial thickness median (IQR), (n = 115)	7.4 (3.9–18)	18.8 (12–26.5)	4.4 (3–5.9)	p < 0.0001^a^

IQR, Interquartile range; BMI, Body Mass Index.

a Mann–Whitney U test.

b Fisher exact.

### Comparative overview of protein biomarkers across sample types

**Fig. 1:**
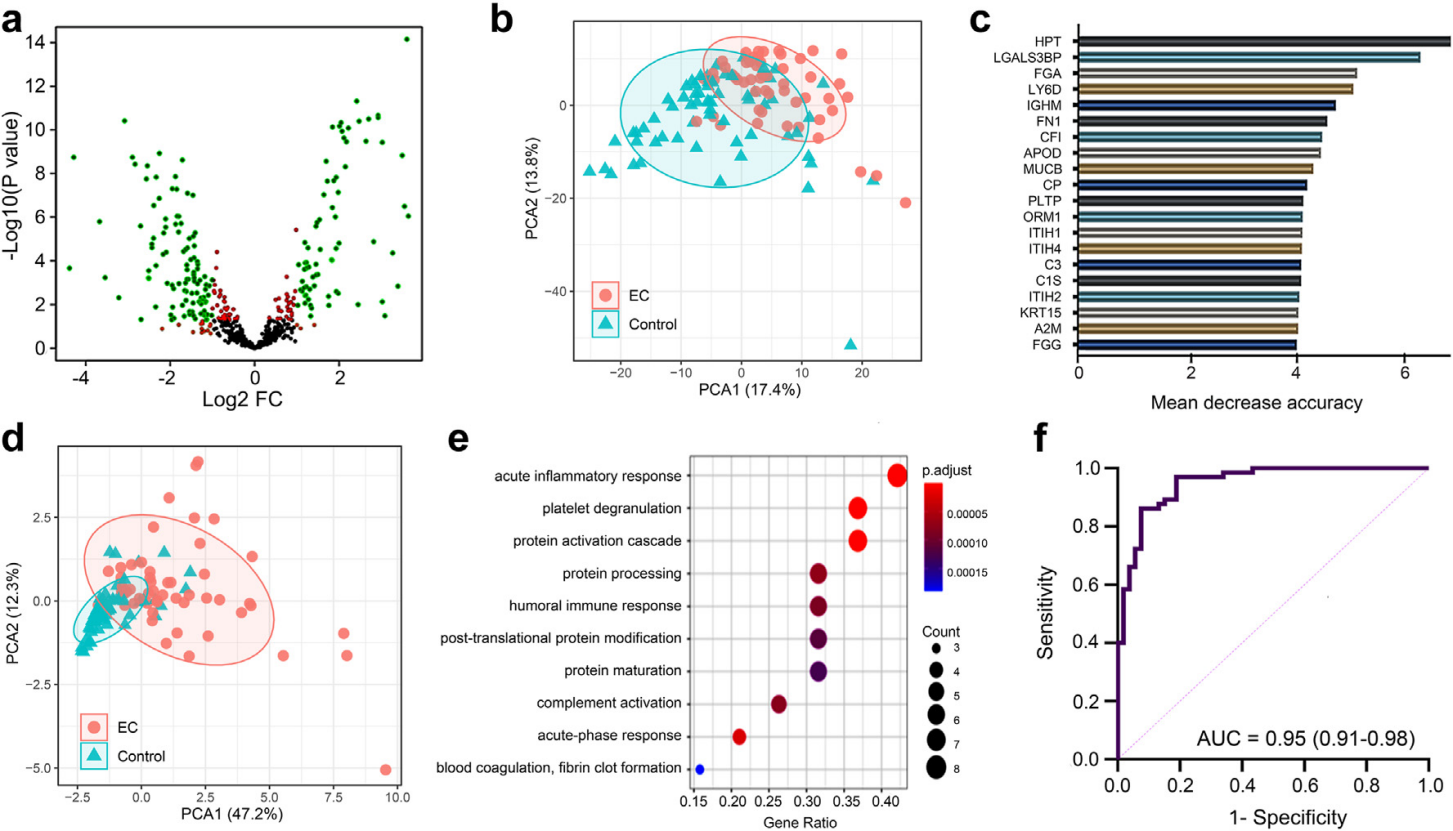
**(a)** Volcano plots summarising the differential expression of cervico-vaginal fluid supernatant proteins based on the degree of log2 fold change and test of statistical significance. Proteins with log2 FC of >1 only are represented as orange dots. Those with p < 0.05 are represented as red dots. Proteins exhibiting log2 FC >1 and p < 0.05 are represented as green dots and those exhibiting neither are represented as black dots. **(b)** Principal component analysis showing discrimination between cancers (n = 53) and controls (n = 65) based on all identified cervico-vaginal fluid supernatant proteins (n = 597 proteins). **(c)** Important discriminatory proteins identified by the random forest machine learning algorithm for cervico-vaginal fluid supernatant proteins and ranked according to their contribution to the overall diagnostic accuracy based on the mean decrease accuracy metric. **(d)** Principal component analysis showing discrimination between cancers and controls based on top ten discriminatory cervico-vaginal supernatant proteins **(e)** Functional pathway analysis of top discriminatory cervico-vaginal fluid supernatant proteins. (f) Diagnostic performance of the parsimonious model of cervico-vaginal fluid supernatant proteins (5-biomarker panel) for the detection of endometrial cancer.

The distribution of identified proteins across the various sample types is summarised in Figure S3. In total, 597 proteins were quantified in the cervico-vaginal fluid supernatant samples while 310 and 533 proteins were quantified in the matched vaginal cell pellet and plasma samples respectively. There were 941 unique proteins across all sample types, including 302 that were exclusive to plasma samples, 203 to vaginal supernatant samples and 29 to vaginal fluid cell pellets. A total of 90 proteins were quantified in all three sample types. The distribution of the differentially expressed proteins with log2 FC >1.0 across sample types is summarised in Figure S3b. There was a statistically significant difference in the proportion of proteins exhibiting log2 FC >1 by sample type (32.5% in cervico-vaginal supernatant vs 13.3% in cell pellets vs 1.3% in plasma, p < 0.05, Chi–Square test). A total of 194 (32.5%) cervico-vaginal fluid supernatant proteins had a log2 FC >1 with the degree of log2 FC ranging between −4.4 and + 3.6 (Fig. 1a, Table S1). For the proteins originating from matched cervico-vaginal fluid cell pellets, 41 (13.3%) had a log2 FC >1.0. The degree of log2 FC here ranged between −1.0 and +3.5 (Table S2). Only 7 (1.3%) plasma proteins had a log2 FC >1.0, and the range of log2 FC was narrow (−1.9 to +1.1) (Table S3).

### Cervico-vaginal fluid derived protein biomarkers for endometrial cancer detection

There was good evidence of separation between the cancers and controls based on all cervico-vaginal supernatant derived proteins (Fig. 1b). The selection of the most important classifiers was based on the mean decrease accuracy metric of the RF model (Fig. 1c). Principal component analyses using the top discriminatory proteins showed a stronger degree of discrimination between cancers and controls (Fig. 1d) and functional pathway analyses confirmed these proteins to have inflammatory, immune and protein regulatory functions (Fig. 1e). Forward stepwise regression modelling, adjusting for age and BMI as continuous variables, was used to create nested logistic regression models based on the most important classifiers, summarised in Table 2. We did not observe any significant interactions when first-order interaction terms were introduced in the models. The sequential addition of the discriminatory proteins increased model complexity and led to an improved model performance, especially with respect to sensitivity and positive predictive value estimates (Table 2). There was a relative plateau in the accuracy metrics between the 5th and subsequent models. The model combining the top-five discriminatory proteins had the least AIC value and was chosen to be the parsimonious model in terms of balancing model and future validated assay simplicity with predictive accuracy (Fig. 1f). This model predicted endometrial cancer with an AUC of 0.95 (95% CI 0.91–0.98), sensitivity of 91% (83%–98%) and specificity of 86% (78%–95%) (Table 2).

**Table 2:** Nested diagnostic model composition and predictive accuracy based on the top discriminatory biomarkers in cervico-vaginal fluid using the mean decrease accuracy metric of the random forest model.

	HPT	LG3BP	FGA	LY6D	IGHM	FN1	AUC (95% CI)	AIC	SEN, % (95% CI)	SPE, % (95% CI)	PPV^a^, % (95% CI)	NPV^a^, % (95% CI)
Model 1	X						0.89 (0.84–0.95)	113	66 (53–79)	85 (76–93)	30 (12–49)	96 (92–99)
Model 2	X	X					0.90 (0.85–0.96)	106	74 (62–85)	86 (78–95)	35 (15–54)	97 (93–100)
Model 3	X	X	X				0.91 (0.87–0.96)	101	83 (73–93)	86 (78–95)	38 (18–57)	98 (95–100)
Model 4	X	X	X	X			0.94 (0.91–0.98)	84	87 (78–96)	86 (78–95)	38 (18–57)	99 (97–100)
Model 5	X	X	X	X	X		0.95 (0.91–0.98)	83	91 (83–98)	86 (78–95)	40 (21–59)	99 (97–100)
Model 6	X	X	X	X	X	X	0.95 (0.91–0.98)	85	91 (83–98)	86 (78–95)	40 (21–59)	99 (97–100)

The order in which the classifiers were entered in the model was determined by their ranking in the mean decrease accuracy metric of random forest. All models were adjusted for age and BMI as continuous variables. AUC, area under the receiver operator characteristic curve; AIC, Akaike information criterion; SEN, sensitivity; SPE, specificity; PPV, positive predictive value; NPV, negative predictive value; CI, Confidence interval. The parsimonious model of cervico-vaginal fluid supernatant proteins for endometrial cancer detection comprised HPT, LG3BP, FGA, LY6D and IGHM.

a Assumed disease prevalence of 9%.

**Fig. 2:**
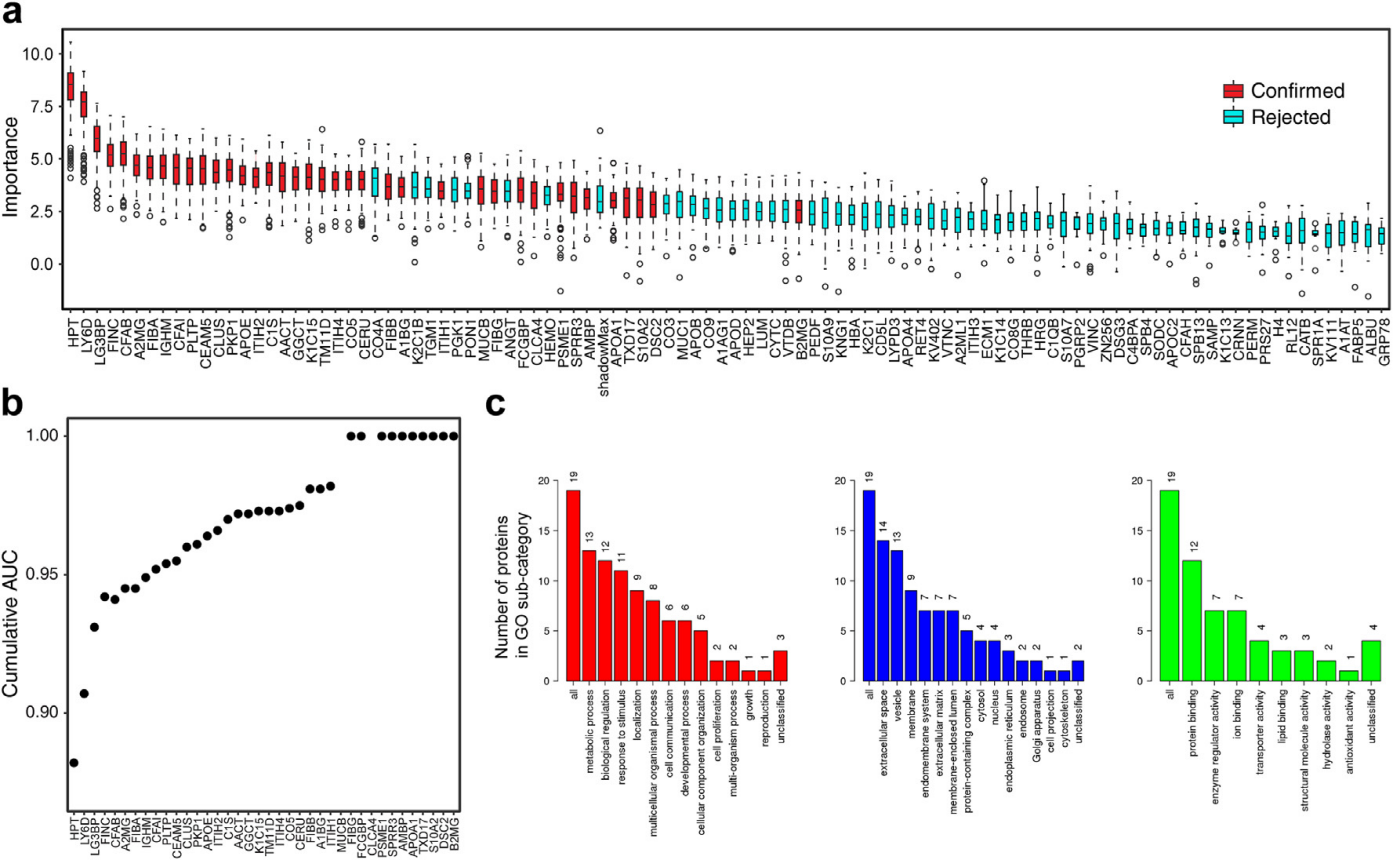
(a) Box plots showing the permutation importance of the cervico-vaginal fluid supernatant proteins confirmed by the Boruta algorithm to be important. (b) Crude cumulative AUC analyses for the Boruta-identified proteins based on multiple forward stepwise logistic regression. (c) Gene ontology analysis of the unique Boruta identified biomarkers using the webserver WebGestalt and showing the biological processes (red), cellular components (blue) and molecular functions (green).

To assess the robustness of the biomarkers identified, we carried out a feature selection analysis using the Boruta algorithm. This identified 38 proteins as being important for the discrimination between cancer and control samples (Fig. 2a). The box-plots of the permutation importance of these proteins are as shown in Fig. 2a. The proteins identified by the Boruta algorithm were consistent with those identified by the accuracy metric of the RF model. A crude cumulative AUC analysis based on a forward stepwise logistic regression modelling of the identified Boruta proteins is presented in Fig. 2b. A gene ontology analysis of the Boruta derived proteins, 19 of which were unique proteins, is summarised in Fig. 2c. Cervico-vaginal fluid cell pellets/debris derived proteins showed less promise in comparison to the supernatant derived proteins as endometrial cancer biomarkers, the findings of which are presented as supplementary data (Tables S2 and S4, Figure S4).

### Plasma protein biomarkers for endometrial cancer detection

**Fig. 3:**
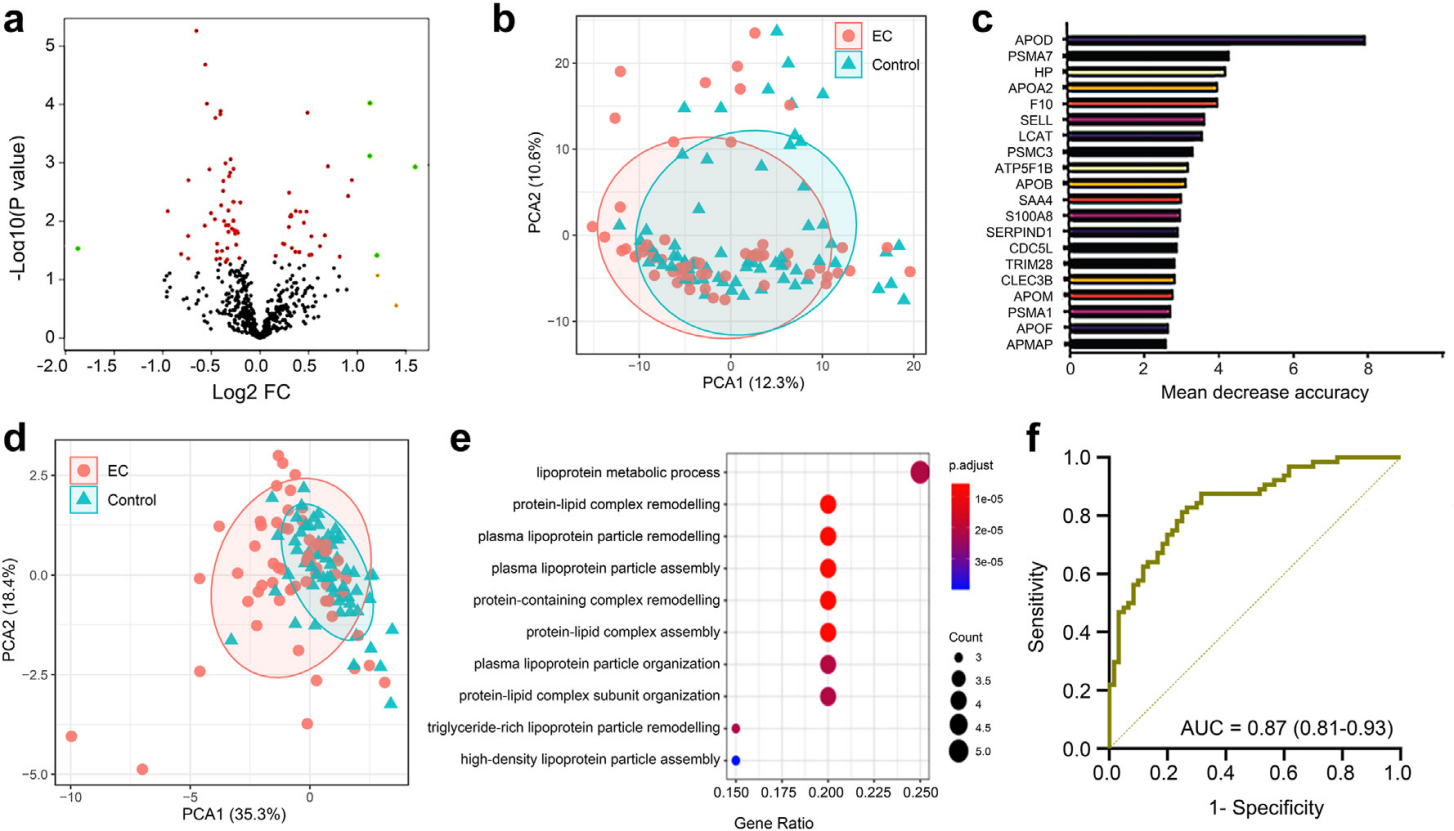
**(a)** Volcano plots summarising the differential expression of plasma proteins based on the degree of log2 fold change and test of statistical significance. Proteins with log2 FC of >1 only are represented as orange dots. Those with p < 0.05 are represented as red dots. Proteins exhibiting log2 FC >1 and p < 0.05 are represented as green dots and those exhibiting neither are represented as black dots. **(b)** Principal component analysis showing discrimination between cancers and controls based on all identified plasma proteins (n = 533 proteins). **(c)** Important discriminatory proteins identified by the random forest machine learning algorithm for plasma proteins and ranked according to their contribution to the overall diagnostic accuracy based on the mean decrease accuracy metric. **(d)** Principal component analysis showing discrimination between cancers and controls based on top ten discriminatory plasma proteins **(e)** Functional pathway analysis of top discriminator plasma proteins. (f) Diagnostic performance of the parsimonious model of plasma proteins (3-biomarker panel) for the detection of endometrial cancer.

Fewer plasma proteins were differentially expressed between cancers and controls compared with cervico-vaginal fluid (Fig. 3a). There was little evidence of sample separation based on all plasma proteins quantified in the study (Fig. 3b). The most important plasma diagnostic classifiers are presented in Fig. 3c. Principal component analysis using the top discriminatory proteins showed modest separation between cancers and controls (Fig. 3d) and the functional pathway analysis of the significant proteins is presented in Fig. 3e. Next, we carried out forward stepwise logistic regression modelling, adjusting for age and BMI, and created multiple nested models for the detection of endometrial cancer in plasma, the performances of which are summarised in Table 3. A 3-marker panel combining APOD, PSMA7 and HPT, predicted endometrial cancer with an AUC of 0.87 (0.81–0.93), sensitivity of 75% (64%–86%), specificity of 84% (75%–93%), and had the least AIC value (Table 3, Fig. 3f). No significant improvement in model performance was observed with incorporation of subsequent classifiers.

**Table 3:** Nested diagnostic model composition and predictive accuracy based on the top discriminatory plasma biomarkers using the mean decrease accuracy metric of the random forest algorithm.

	APOD	PSMA7	HPT	APOA2	FA10	SELL	AUC (95% CI)	AIC	SEN, % (95% CI)	SPE, % (95% CI)	PPV^a^, % (95% CI)	NPV^a^, % (95% CI)
Model 1	X						0.84 (0.77–0.91)	133	73 (62–85)	81 (72–91)	29 (12–45)	97 (93–100)
Model 2	X	X					0.86 (0.80–0.93)	127	75 (64–86)	84 (75–93)	32 (14–50)	97 (93–100)
Model 3	X	X	X				0.87 (0.81–0.93)	126	75 (64–86)	84 (75–93)	32 (14–50)	97 (93–100)
Model 4	X	X	X	X			0.87 (0.81–0.93)	126	75 (64–86)	80 (67–88)	28 (11–44)	97 (93–100)
Model 5	X	X	X	X	X		0.86 (0.81–0.93)	127	75 (64–86)	81 (72–91)	29 (12–45)	97 (93–100)
Model 6	X	X	X	X	X	X	0.87 (0.81–0.93)	130	77 (66–87)	81 (72–91)	29 (12–45)	98 (95–100)

The order in which the classifiers were entered in the model was determined by their ranking in the mean decrease accuracy metric of random forest. All models were adjusted for age and BMI as continuous variables. AUC, area under the receiver operator characteristic curve; AIC, Akaike information criterion; SEN, sensitivity; SPE, specificity; PPV, positive predictive value; NPV, negative predictive value; CI, Confidence interval. The parsimonious model of plasma proteins for endometrial cancer detection comprised APOD, PSMA7 and HPT.

a Assumed disease prevalence of 9%.

**Fig. 4:**
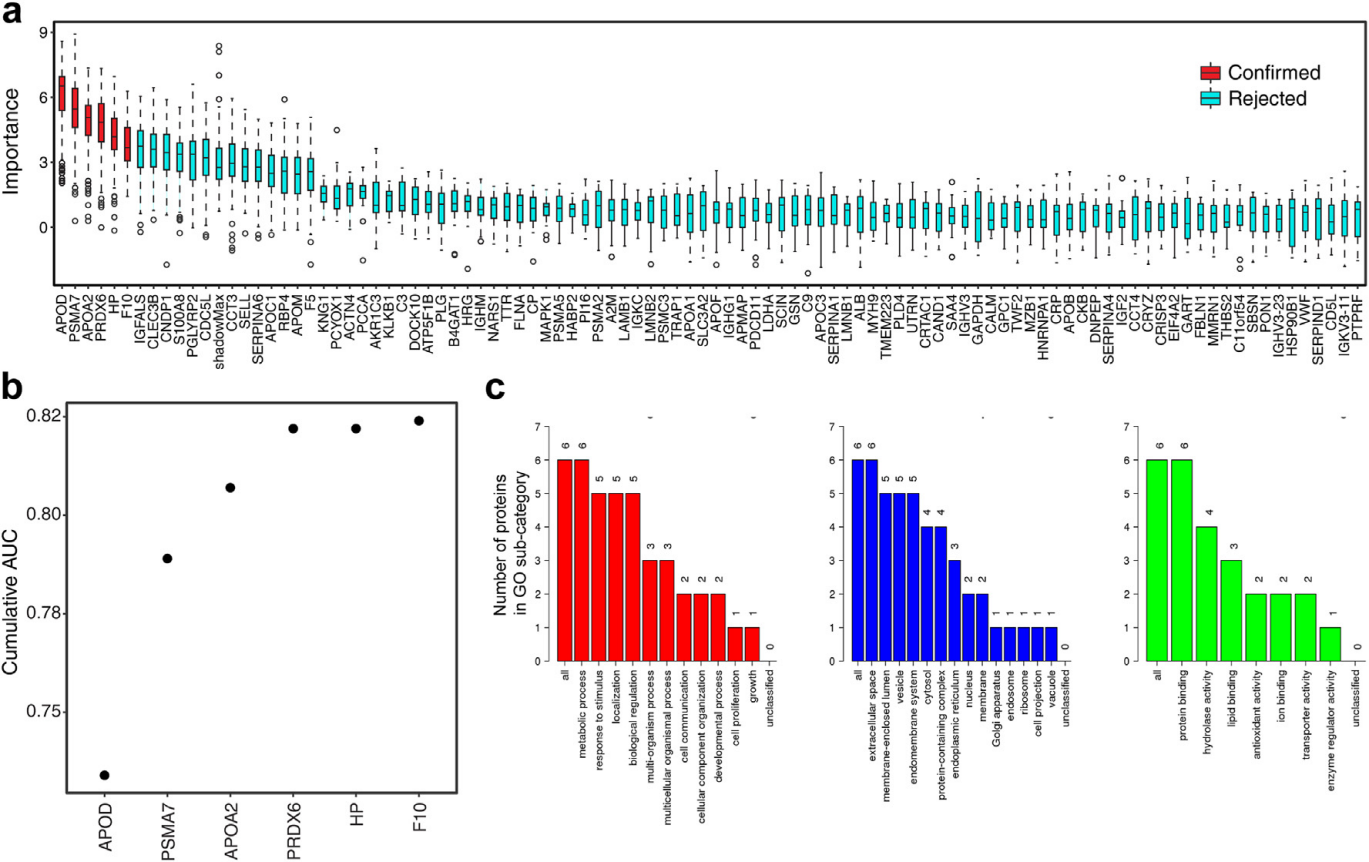
**(a)** Box plots showing the permutation importance of the plasma proteins confirmed by the Boruta algorithm to be important. **(b)** Crude cumulative AUC analyses for the Boruta-identified plasma proteins based on multiple forward stepwise logistic regression. **(c)** Gene ontology analysis of the unique Boruta identified plasma biomarkers using the webserver WebGestalt and showing biological processes (red), cellular components (blue) and molecular functions (green).

To assess the robustness of the identified plasma proteins in detecting endometrial cancer, we carried out feature selection using the Boruta algorithm. This identified 6 proteins as being important consistent with the top discriminatory biomarkers identified by the RF model (Fig. 4a). The box-plots of the permutation's importance of the proteins, crude cumulative AUC and gene ontology analyses are presented in Fig. 4.

A correlation analyses between the overlapping plasma and cervico-vaginal fluid derived biomarkers is presented in Table S5.

### Proteomic analysis of cervico-vaginal fluid and plasma for the detection of early-stage, advanced stage and non-endometrioid endometrial cancers

Early detection of endometrial cancer is key to achieving best survival outcomes. We therefore sought to identify protein biomarker signatures across the various sample types that can distinguish early stage (FIGO stage 1) endometrial cancer (n = 41) from controls (n = 65) (Table S6). A three-marker panel of cervico-vaginal fluid derived proteins comprising HPT, LY6D, and C5 predicted stage I endometrial cancer with an AUC of 0.92 (0.87–0.97) (Table S6). For plasma proteins, a 4-marker panel combining CNDP1, CDC5L, APOD and PRDX6 had the least AIC value and predicted early-stage endometrial cancer with an AUC of 0.88 (0.82–0.95) (Table S7).

A clinically translatable endometrial cancer biomarker assay should in addition to detecting early-stage disease be able to identify endometrial cancers that are not confined to the uterus. We therefore sought to describe biomarkers that can aid the detection of FIGO stages II-IV endometrial cancer. A 5-biomarker panel of cervico-vaginal fluid proteins combining APOE, GGCT, CFAB, LY6D and CEAM5 predicted advanced stage endometrial cancer with AUC 0.96 (0.92–1.00). For plasma proteins, a six-biomarker panel combining SERPINA4, APOA2, TTR, CRP, CLEC3B and APOD predicted advanced stage endometrial cancer with AUC 0.93 (0.85–0.99).

Early detection of the biologically aggressive tumours will improve outcomes. Classical univariate ROC curve analyses confirmed the discriminatory potential of cervico-vaginal fluid derived PLTP, A2MG, APOE, FIBB, CO5 and FIBA for the detection of non-endometrioid endometrial cancers with AUC's >0.97 respectively. A biomarker signature incorporating these six proteins predicted non-endometrioid endometrial cancer with AUC 0.99 (0.98–1.00). A combined panel of five plasma proteins, namely SELL, CCT3, IGF2, IFGALS, and IGFBP3 predicted non-endometrioid endometrial cancer with AUC of 0.88 (0.77–1.00).

## Discussion

### Main findings

In this study, we exploit the anatomical continuity between the uterine cavity and the lower genital tract to identify protein signatures that detect endometrial cancer in cervico-vaginal fluid with high accuracy. Using machine learning technique, robust predictive models that distinguish women with endometrial cancer from symptomatic controls were developed. Cervico-vaginal fluid derived protein signatures demonstrated better accuracy for endometrial cancer detection when compared with plasma proteins. A 5-biomarker signature of cervico-vaginal fluid proteins combining HPT, LG3BP, FGA, LY6D and IGHM predicted endometrial cancer with an AUC of 0.95 (0.91–0.98), sensitivity of 91%, and specificity of 86%. By contrast, a 3-marker panel of plasma proteins combining APOD, PSMA7 and HPT predicted endometrial cancer with an AUC of 0.87 (0.81–0.93), sensitivity of 75%, and specificity of 84%. Cervico-vaginal fluid derived biomarker panels outperformed those derived from plasma in detecting early-stage, advanced stage and biologically aggressive endometrial cancer phenotypes. These data suggest that cervico-vaginal fluid protein panels could offer simple, minimally invasive endometrial cancer detection tools and enable innovative point of care tests that could transform patient care.

### Strengths and limitations

This study has several strengths. We report a promising endometrial cancer detection tool that is based on the minimally invasive sampling of proteins expressed in the cervico-vaginal fluid of women with or at risk of endometrial cancer. Our cervico-vaginal fluid samples were collected using a Delphi screener, a device that has shown superiority in terms of reproducibility, sample quality and patient-acceptability, when compared with other sample collection methods.^9^^,^^17^ Lower mean pain scores have been reported for this device, in comparison to diagnostic hysteroscopy and endometrial biopsy, thus enhancing its translational potential.^17^ The Delphi device has the added advantage of being a self-sampling device and can be used in the community setting by practice nurses and clinicians.^9^ The choice of our control group, composed of symptomatic post-menopausal women, is another strength as this constitutes the ideal comparison group for endometrial cancer biomarker discovery studies, since these are the women for whom the new test is intended. The protein panels identified in this study showed sufficient accuracy for the detection of early-stage as well as advanced stage endometrial cancers and thus have good potential for clinical translation. Indeed, many of the proteins have mechanistic links with either the malignant transformation process or the body's immunological (inflammatory) response to malignancy. Availability of matched plasma samples allowed for a comparative analyses of biomarker performance in plasma. Due to the limited sample size of the cohort enrolled in this study, we cannot exclude the possibility that accuracy estimation for the parsimonious biomarker models may be biased by overfitting, especially for the advanced stage and non-endometrioid tumours. Studies on larger cohorts are needed to validate the cervico-vaginal fluid and plasma signatures reported. Whilst we adjusted for age and BMI, our findings remain open to residual confounding factors. Our study is also prone to verification bias and other inherent limitations of a case–control study design. However, control participants were followed up for 12 months, thus minimising the possibility of misclassification. Our lack of data on the molecular classification of endometrial cancer precluded our ability to explore biomarkers for more specific endometrial cancer molecular phenotypes. Furthermore, we need to define how well these protein panels will perform in asymptomatic women, or pre-menopausal women with a genetic predisposition to endometrial cancer as seen in Lynch syndrome. As the study participants were mostly of White British ethnicity, we cannot necessarily extrapolate the findings to women from other nationalities or ethnic backgrounds. Further studies are needed to confirm the potential utility of these biomarker panels in other populations.

### Interpretation

The incidence of endometrial cancer is continuing to rise across high-income countries, alongside growing rates of obesity.^1^ Early detection remains the cornerstone of endometrial cancer control but is limited by the lack of simple, patient-friendly, minimally invasive detection tools.^2^ Cervico-vaginal fluid and plasma are viable sources of cancer-derived biomarkers and have potential for clinical translation due to their easy accessibility and high acceptability.^7^ A number of cervico-vaginal fluid proteins were identified as important biomarkers for the detection of endometrial cancer, many of which have mechanistic links with the malignant transformation process. Galectin-3-binding protein (LG3BP) was significantly increased in endometrial cancer cases and showed promise as an endometrial cancer diagnostic biomarker. LG3BP plays a crucial role in integrin mediated cell adhesion and stimulates the host defence against viruses and tumour cells.^18^ Studies have been consistent in demonstrating pro-tumorigenic properties for LG3BP via its regulation of cell proliferation, apoptosis, cell adhesion, angiogenesis, and metastasis by binding to cell surface beta-galactose-containing glycoconjugates or glycolipids.^18^, ^19^, ^20^ LG3BP has also been reported to be upregulated in other malignancies including those of the colorectum,^21^ central nervous system,^22^ stomach,^19^ lung^23^ and breast.^20^ Lymphocyte antigen 6D (LY6D) identified endometrial cancer with an AUC of 0.89 and was able to predict early-stage tumours with good accuracy. LY6D plays an important role in lymphocyte differentiation, cell adhesion, cancer progression and immune escape.^24^^,^^25^ This biomarker has been reported to be associated with distant metastasis in women with oestrogen receptor positive breast cancer^26^ and has also been shown to be prognostic in hepatocellular malignancies and cancers of the head and neck.^27^^,^^28^

Immunoglobulins, including the immunoglobulin heavy constant mu (IGHM), were identified as potential endometrial cancer biomarkers. Immunoglobulins are antibodies produced by the B lymphocytes that play crucial roles in the body's primary defence mechanisms.^29^ They are involved in the early recognition and elimination of external invaders, including pre-cancerous and cancerous lesions. The up-regulation of IGHM in the cervico-vaginal fluid of women with endometrial cancer is likely to result from an immunological response to the presence of the malignancy.^30^ However, there is evidence to suggest that endometrial cancers harbour more mutations in the IGHM protein, compared to cancers of other sites.^31^ Further studies are needed to validate these biomarkers prior to clinical usage. HPT is an acute-phase glycoprotein that regulates the immune response.^32^ Serum HPT levels have been reported to be elevated in several malignancies, including those of the lung, breast and ovary.^15^^,^^33^ FGA is a cell adhesion molecule and a cancer-related gene that has been reported as a biomarker in endometrial cancer.^5^ Studies elucidating the mechanistic links of these markers in endometrial carcinogenesis are urgently needed.

Our findings are consistent with previous proof-of-concept studies in demonstrating the feasibility of detecting endometrial cancer by leveraging high-throughput technologies on clinical samples acquired using minimally invasive sampling. Herzog and colleagues, using cervical smear specimens obtained from 726 women with and without endometrial cancer, and validated in 562 cervico-vaginal fluid samples, identified a 3-marker assay for endometrial cancer detection based on DNA methylation changes in gene regions of GYPC and ZSCAN12.^34^ This test detected endometrial with sensitivities of 97.2%, 90.1% and 100% in cervical, self-collected genital, and vaginal swab samples respectively. This study was limited by its case–control design and low yield of DNA in up to 12% of self-collected samples. The PapSEEK test, a novel detection tool incorporating assays for mutations in 18 genes as well as aneuploidy in Pap brush samples acquired from 382 women with endometrial cancer demonstrated a detection rate of 81%. When a Tao brush was used, a higher detection accuracy of 93% was reported.^35^ Whilst the smear test is widely acceptable to women, it is an intimate procedure and embarrassment and discomfort of speculum examination are common reasons for non-uptake. He and colleagues reported higher levels of cancer antigen 125 in cervico-vaginal secretions of women with endometrial cancer (n = 148) compared to controls (n = 77), a finding that aligns with the small pilot study by Calis et al. who found that at a threshold of 575 μ/ml, cervico-vaginal CA125 detects endometrial precancer or cancer with a sensitivity of 78%.^36^ The sub-optimal sensitivity has clinical implications including risk of false reassurance and delayed presentation. We did not identify cervico-vaginal fluid CA125 as an important biomarker for endometrial cancer detection.

Using targeted proteomics, Martinez-Garcia and colleagues explored the levels of 52 proteins in the fluid fraction of uterine aspirates acquired from 69 women with endometrial cancer and 47 controls and found that the biomarker panel combining MMP9 and KPYM detected endometrial cancer with 94% sensitivity and 87% specificity.^37^ These findings are yet to be validated for potential clinical translation. Further evidence can be found in the study by Bakkum-Gamez and colleagues who, using tampon collected vaginal fluid, showed that a 28-methylated DNA marker discriminated between endometrial cancers and controls with 76% sensitivity at 96% specificity (AUC 0.88).^38^ However, age differences in both the discovery and validation cohorts exist which may have impacted on the study results. Moreover, vaginal tampons are generally unappealing to post-menopausal women and may be inadequate for endometrial cancer detection in women without bleeding symptoms. The case–control study by Pelegrina and colleagues found that 73% of cervico-vaginal fluid samples acquired from women with endometrial cancer had detectable somatic mutations.^39^ These findings are consistent with the growing body of evidence of the potential to detect endometrial cancer based on minimally invasive sampling methodologies.^40^, ^41^, ^42^

Cervico-vaginal fluid protein signatures outperformed plasma proteins in detecting endometrial cancer. This is in keeping with the proximal nature of cervico-vaginal fluids which derive from or have been in direct contact with endometrial tumours.^5^ The significantly reduced protein dynamic range in cervico-vaginal fluids compared to plasma allows for better sensitivity for the detection of clinically relevant biomarkers in the former.^14^ Cervico-vaginal fluid supernatant protein biomarkers showed more promise than those derived from cell pellets/debris. This may be related to the fact that the cell pellets were most likely dominated by normal epithelial cells of the female genital tract rather than endometrial cancer cells shed down the lower genital tract, creating a background matrix that limits the detection of differentially expressed proteins.

Our finding of plasma apolipoproteins and haptoglobin as potential endometrial cancer biomarkers is consistent with previous work.^5^ Takano and colleagues in a protein profiling study of serum samples acquired from 65 endometrial cancer cases and 40 controls reported that serum apolipoprotein A-1 detects endometrial cancer with 75% sensitivity while the combined panel of apolipoproteins A1 and C-1 exhibited a sensitivity of 82%. Several other blood-based biomarker candidates for endometrial cancer detection have been reported, but the evidence to warrant clinical translation is limited.^5^ Cervico-vaginal protein signatures were consistent in accurately detecting early-stage, locally advanced or metastatic disease and non-endometrioid cancers with AUC values >0.90. Plasma protein signatures, on the other hand, showed moderate accuracy for the detection of early-stage and biologically aggressive endometrial tumours. However, for locally advanced/metastatic disease, plasma proteins had good accuracy with AUC of 0.93. These findings must be interpreted with caution given the small sample size. However, it is in keeping with the low-yield in plasma of cancer related-signals in early-stage disease vs advanced stage disease.^7^ Cervico-vaginal fluid protein signatures performed best for advanced and biologically aggressive endometrial tumours (AUC 0.96 vs 0.99) compared with early-stage cancers (AUC 0.92), suggesting that advanced and biologically aggressive cancers are more likely to shed protein biomarkers down the lower genital tract in sufficient quantity to be detected in the cervico-vaginal fluid than early-stage cancers. These findings have important clinical implications. Cervico-vaginal fluid biomarker signatures demonstrated high accuracy for high-grade and biologically aggressive cancers, the early detection of which significantly improves outcomes.^2^ The protein signatures identified in this study out-performed clinical risk predictors such as age, BMI and even endometrial thickness, the current gold standard triage tool, in predicting endometrial cancer. These protein panels thus have the potential to replace or be used alongside transvaginal ultrasound scan in symptomatic women to direct further investigations and may offer screening tools for those with a genetic predisposition to endometrial cancer as seen in Lynch syndrome.

The use of SWATH-MS for the detection of endometrial cancer in cervico-vaginal fluid is presently not easily translatable to clinical settings. We envisage that the successful application of the biomarker signatures to clinical practice will be dependent on the development of clinically actionable assays based on ELISA, Lumipulse ® technology, or even lateral flow test technology for point of care testing. A validated cervico-vaginal fluid multi-protein biomarker assay based on any of these platforms can lead to rapid discrimination of symptomatic women with and without endometrial cancer, with substantial cost-saving implications for health service providers, whilst saving healthy women from invasive tests that are distressing and avoidable in over 90% of cases.^16^

In conclusion, cervico-vaginal fluid proteins sampled using a Delphi screener offer a simple, relatively patient-friendly and easy to administer detection tool for endometrial cancer. A combined panel of proteins showed great promise with AUCs >0.90 for endometrial cancer detection. These data can inform the development of innovative point of care diagnostic tests that can transform patient care. Confirmatory studies using immunoassays or targeted proteomics in a larger study cohort are needed to validate these biomarker candidates and to elucidate their role in endometrial carcinogenesis.

## Contributors

K.N. recruited study participants, collected relevant samples, performed study experiments, analysed/interpreted data and wrote the manuscript. K.N, A.P, D.C, A.D.W, and E.J.C designed laboratory study. A.P and D.C contributed to study experiments, data interpretation and analysis, B.G and A.E.C contributed to data interpretation and analysis, J.K and R.R contributed to study experiments and data interpretation, N.G contributed to data interpretation and analysis. E.J.C. conceptualised the study. E.J.C. and A.D.W. supervised study execution, contributed to data interpretation and edited the manuscript. E.J.C. is Principal Investigator and obtained funding for the study. K.N., A.E.C, and D.C verified the underlying data. All authors provided critical comment. All authors have read and agreed to the published version of the manuscript.

## Data sharing statement

The mass spectrometry proteomics data have been deposited in the ProteomeXchange Consortium via the PRIDE^43^ partner repository with identifier PXD050276.

## Declaration of interests

The authors declare no conflict of interest.

## Appendix A Supplementary data

Suppementary Tables S1–S7 and Figures S1–S4
