# Supplementary material for: Detection of endometrial cancer in cervico-vaginal fluid and blood plasma: leveraging proteomics and machine learning for biomarker discovery
Source: eBioMedicine. 2024 Mar 20;102:105064. doi: 10.1016/j.ebiom.2024.105064 (PMC10960138; doi:10.1016/j.ebiom.2024.105064)
Supplement: Suppementary Tables S1–S7 and Figures S1–S4 [file mmc1.docx]

**Supplementary Figures and Tables (online only)**

**Supplementary Figure S1** (Graphical abstract)


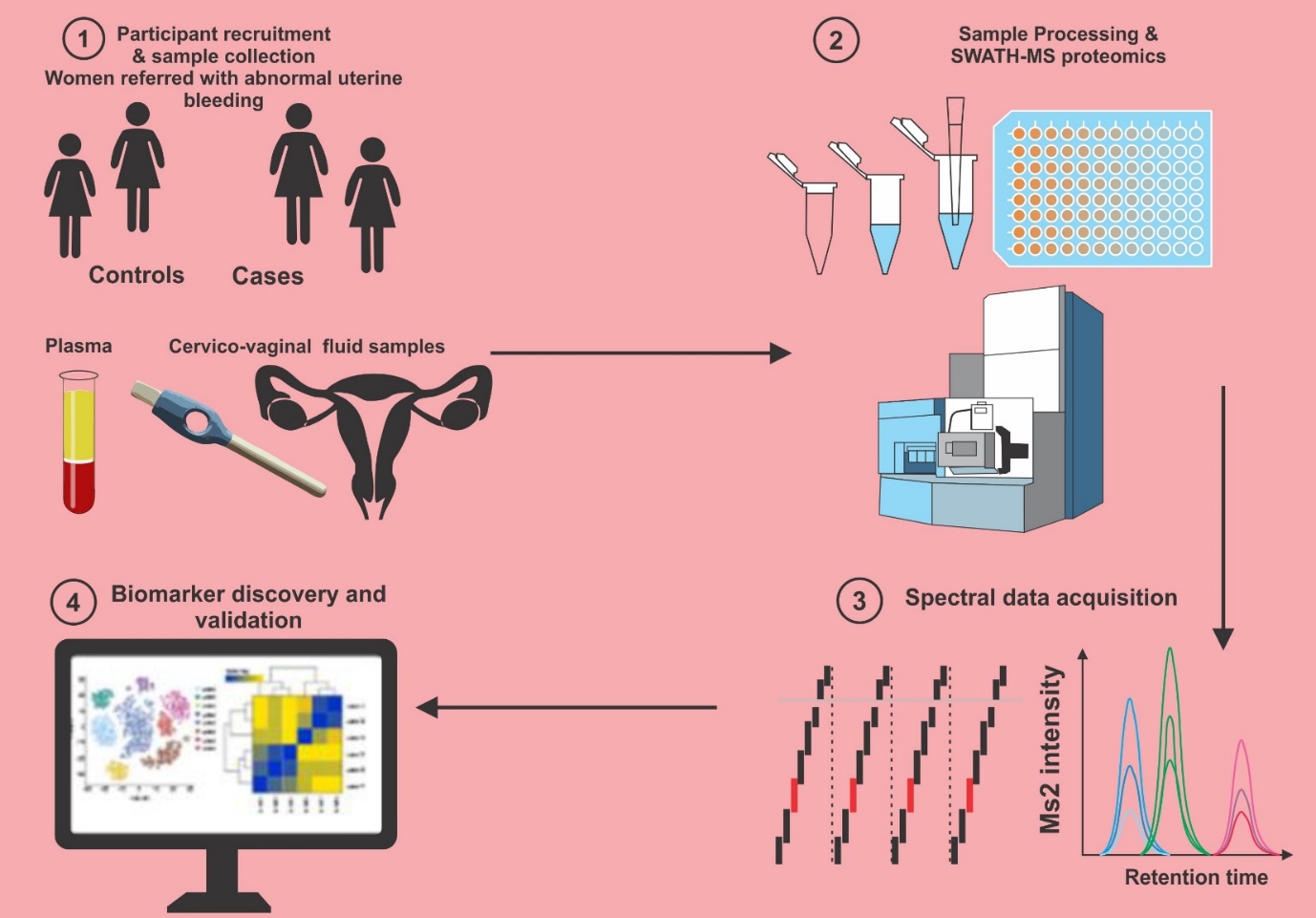


**Figure S2: A STARD (2015) diagram showing the flow of participants enrolled in the study.**


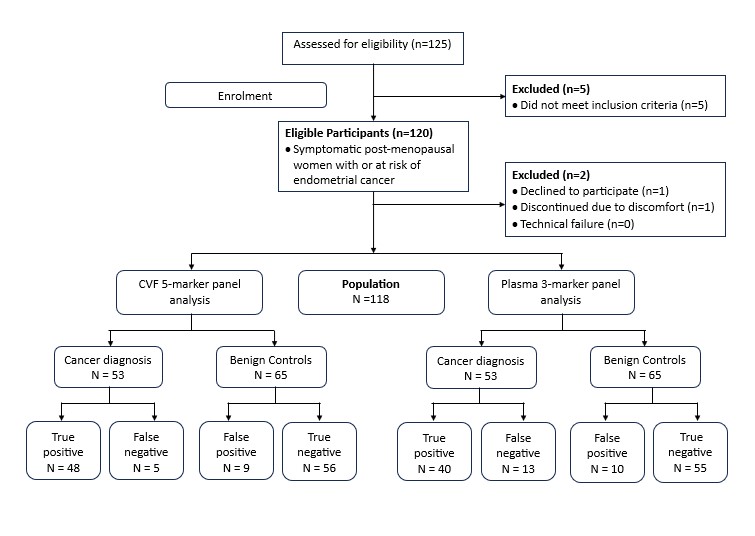


CVF= Cervico-vaginal fluid.

**Supplementary Figure S3**: Venn diagrams showing (A) Distribution of all proteins quantified in cervico-vaginal fluid supernatant (green), cell pellet (blue) and plasma (yellow) samples. (B) Distribution of proteins exhibiting a log2 FC >1.0 across sample types


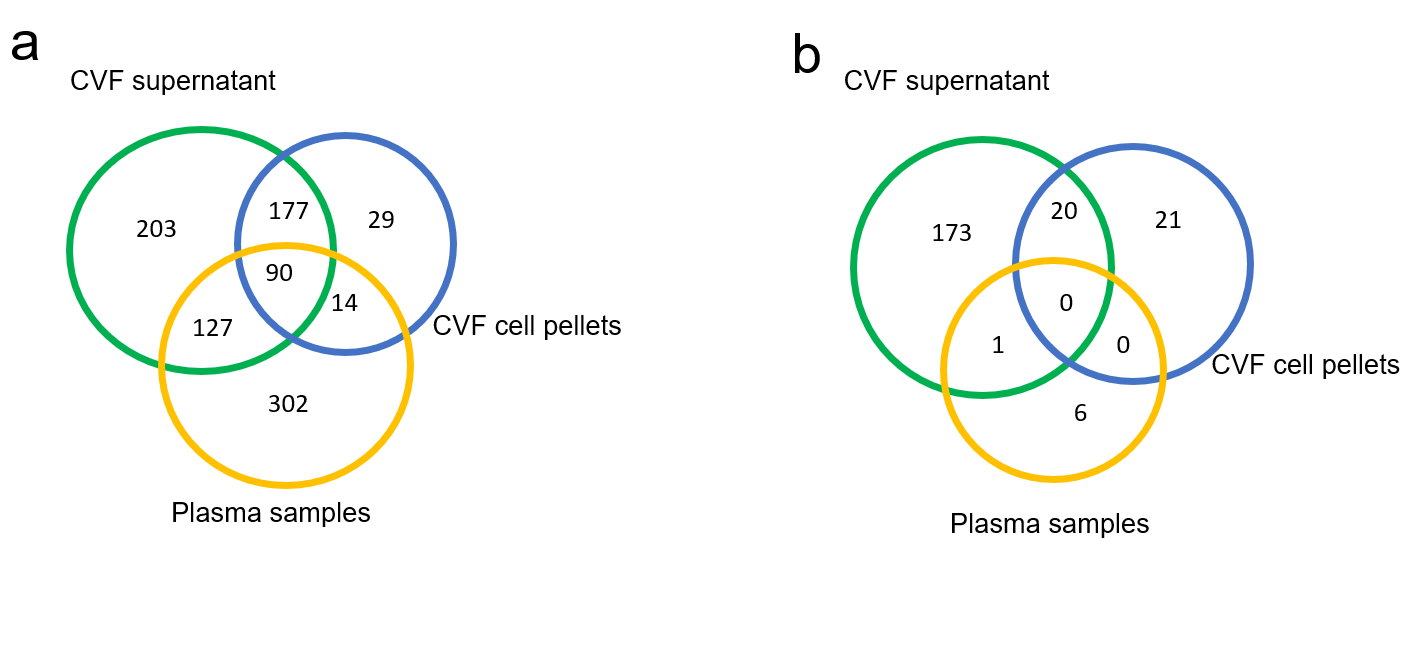


**Supplementary Table S1**: List of identified proteins and their log2 fold change values comparing cases and controls in cervico-vaginal fluid supernatant samples with unadjusted and adjusted p values (Benjamini–Hochberg correction method).

| **Proteins** | **Log2FC** | **P-value** | **Adjusted P-value** |
| --- | --- | --- | --- |
| P00738\|HPT_HUMAN | 3.609 | <0.0001 | <0.0001 |
| P00751\|CFAB_HUMAN | 2.417 | <0.0001 | <0.0001 |
| P02790\|HEMO_HUMAN | 2.928 | <0.0001 | <0.0001 |
| P02675\|FIBB_HUMAN | 2.927 | <0.0001 | <0.0001 |
| P01023\|A2MG_HUMAN | 2.702 | <0.0001 | <0.0001 |
| P02763\|A1AG1_HUMAN | 2.474 | <0.0001 | <0.0001 |
| P22735\|TGM1_HUMAN | -3.080 | <0.0001 | <0.0001 |
| P01871\|IGHM_HUMAN | 2.047 | <0.0001 | <0.0001 |
| P01024\|CO3_HUMAN | 2.011 | <0.0001 | <0.0001 |
| P02760\|AMBP_HUMAN | 1.845 | <0.0001 | <0.0001 |
| Q14624\|ITIH4_HUMAN | 2.221 | <0.0001 | <0.0001 |
| P04217\|A1BG_HUMAN | 2.072 | <0.0001 | <0.0001 |
| P01019\|ANGT_HUMAN | 2.116 | <0.0001 | <0.0001 |
| P00450\|CERU_HUMAN | 2.639 | <0.0001 | <0.0001 |
| P02671\|FGA_HUMAN | 2.173 | <0.0001 | <0.0001 |
| P02679\|FIBG_HUMAN | 3.027 | <0.0001 | <0.0001 |
| P06702\|S10A9_HUMAN | -2.253 | <0.0001 | <0.0001 |
| P01009\|A1AT_HUMAN | 3.497 | <0.0001 | <0.0001 |
| Q01469\|FABP5_HUMAN | -2.892 | <0.0001 | <0.0001 |
| Q9UBC9\|SPRR3_HUMAN | -4.285 | <0.0001 | <0.0001 |
| O75223\|GGCT_HUMAN | -1.710 | <0.0001 | <0.0001 |
| P02751\|FINC_HUMAN | 1.694 | <0.0001 | <0.0001 |
| P31151\|S10A7_HUMAN | -2.830 | <0.0001 | <0.0001 |
| A8K2U0\|A2ML1_HUMAN | -2.539 | <0.0001 | <0.0001 |
| P01042\|KNG1_HUMAN | 2.149 | <0.0001 | <0.0001 |
| P13987\|CD59_HUMAN | -1.874 | <0.0001 | <0.0001 |
| P04196\|HRG_HUMAN | 1.931 | <0.0001 | <0.0001 |
| Q14210\|LY6D_HUMAN | -2.331 | <0.0001 | <0.0001 |
| O60235\|TM11D_HUMAN | -2.562 | <0.0001 | <0.0001 |
| P10909\|CLUS_HUMAN | 1.875 | <0.0001 | <0.0001 |
| P02787\|TRFE_HUMAN | 1.721 | <0.0001 | <0.0001 |
| P80188\|NGAL_HUMAN | -1.948 | <0.0001 | <0.0001 |
| P06731\|CEAM5_HUMAN | -1.845 | <0.0001 | <0.0001 |
| P02774\|VTDB_HUMAN | 1.980 | <0.0001 | <0.0001 |
| P30740\|ILEU_HUMAN | -1.589 | <0.0001 | <0.0001 |
| P05156\|CFAI_HUMAN | 1.639 | <0.0001 | <0.0001 |
| P18206\|VINC_HUMAN | -1.461 | <0.0001 | <0.0001 |
| P01040\|CYTA_HUMAN | -2.258 | <0.0001 | <0.0001 |
| P07355\|ANXA2_HUMAN | -2.388 | <0.0001 | <0.0001 |
| P68871\|HBB_HUMAN | 3.551 | <0.0001 | <0.0001 |
| P02748\|CO9_HUMAN | 1.841 | <0.0001 | <0.0001 |
| P02647\|APOA1_HUMAN | 3.643 | <0.0001 | <0.0001 |
| P10412\|H14_HUMAN | -1.795 | <0.0001 | <0.0001 |
| P16403\|H12_HUMAN | -1.832 | <0.0001 | <0.0001 |
| P04114\|APOB_HUMAN | 1.913 | <0.0001 | <0.0001 |
| Q7Z794\|K2C1B_HUMAN | -2.108 | <0.0001 | <0.0001 |
| P01008\|ANT3_HUMAN | 1.536 | <0.0001 | <0.0001 |
| P07476\|INVO_HUMAN | -3.673 | <0.0001 | <0.0001 |
| Q08380\|LG3BP_HUMAN | 1.478 | <0.0001 | <0.0001 |
| Q02487\|DSC2_HUMAN | -1.740 | <0.0001 | <0.0001 |
| P04083\|ANXA1_HUMAN | -1.870 | <0.0001 | <0.0001 |
| Q06323\|PSME1_HUMAN | -2.700 | <0.0001 | <0.0001 |
| P02766\|TTHY_HUMAN | 1.452 | <0.0001 | <0.0001 |
| P29508\|SPB3_HUMAN | -2.105 | <0.0001 | <0.0001 |
| P25311\|ZA2G_HUMAN | 0.983 | <0.0001 | <0.0001 |
| P00441\|SODC_HUMAN | -1.800 | <0.0001 | <0.0001 |
| Q13813\|SPTN1_HUMAN | -1.409 | <0.0001 | <0.0001 |
| P16401\|H15_HUMAN | -2.153 | <0.0001 | <0.0001 |
| P05109\|S10A8_HUMAN | -1.451 | <0.0001 | <0.0001 |
| Q16610\|ECM1_HUMAN | -2.395 | <0.0001 | <0.0001 |
| P10599\|THIO_HUMAN | -1.545 | <0.0001 | <0.0001 |
| P69905\|HBA_HUMAN | 2.821 | <0.0001 | 0.00013 |
| P02749\|APOH_HUMAN | 1.191 | <0.0001 | 0.00014 |
| P05546\|HEP2_HUMAN | 1.999 | <0.0001 | 0.00015 |
| P04264\|K2C1_HUMAN | -2.433 | <0.0001 | 0.00016 |
| Q9BQR3\|PRS27_HUMAN | -1.428 | <0.0001 | 0.00019 |
| P02765\|FETUA_HUMAN | 1.264 | <0.0001 | 0.00022 |
| P54108\|CRIS3_HUMAN | -1.589 | <0.0001 | 0.00022 |
| P29966\|MARCS_HUMAN | -2.430 | <0.0001 | 0.00022 |
| P02533\|K1C14_HUMAN | 1.930 | <0.0001 | 0.00023 |
| P02768\|ALBU_HUMAN | 1.553 | <0.0001 | 0.00025 |
| Q9Y6R7\|FCGBP_HUMAN | -1.635 | <0.0001 | 0.00025 |
| P63313\|TYB10_HUMAN | -1.682 | <0.0001 | 0.00030 |
| Q15365\|PCBP1_HUMAN | -0.890 | <0.0001 | 0.00032 |
| Q9UBG3\|CRNN_HUMAN | -2.093 | <0.0001 | 0.00032 |
| P01011\|AACT_HUMAN | 3.272 | <0.0001 | 0.00035 |
| P19012\|K1C15_HUMAN | -2.166 | <0.0001 | 0.00039 |
| Q9UIV8\|SPB13_HUMAN | -1.435 | <0.0001 | 0.00056 |
| P11021\|GRP78_HUMAN | -1.512 | <0.0001 | 0.00067 |
| P04004\|VTNC_HUMAN | 1.823 | <0.0001 | 0.00067 |
| P01860\|IGHG3_HUMAN | 1.833 | <0.0001 | 0.00069 |
| Q14CN2\|CLCA4_HUMAN | -1.356 | 0.00011 | 0.00079 |
| P02743\|SAMP_HUMAN | 1.226 | 0.00012 | 0.00087 |
| P01031\|CO5_HUMAN | 1.196 | 0.00012 | 0.00089 |
| P19823\|ITIH2_HUMAN | 1.453 | 0.00014 | 0.00100 |
| Q92876\|KLK6_HUMAN | -1.346 | 0.00014 | 0.00100 |
| P00734\|THRB_HUMAN | 1.044 | 0.00015 | 0.0010 |
| P03973\|SLPI_HUMAN | -1.606 | 0.00016 | 0.0011 |
| Q09666\|AHNK_HUMAN | -2.334 | 0.00017 | 0.0011 |
| P08603\|CFAH_HUMAN | 1.282 | 0.00019 | 0.0013 |
| P02753\|RET4_HUMAN | 1.236 | 0.00021 | 0.0014 |
| P04632\|CPNS1_HUMAN | -0.908 | 0.00021 | 0.0014 |
| P13646\|K1C13_HUMAN | -4.386 | 0.00022 | 0.0014 |
| P32926\|DSG3_HUMAN | -1.352 | 0.00023 | 0.0015 |
| P00747\|PLMN_HUMAN | 1.182 | 0.00025 | 0.0015 |
| P08727\|K1C19_HUMAN | -1.598 | 0.00026 | 0.0016 |
| Q08188\|TGM3_HUMAN | -2.507 | 0.00028 | 0.0018 |
| Q96FQ6\|S10AG_HUMAN | -1.442 | 0.00034 | 0.0021 |
| P29401\|TKT_HUMAN | 1.497 | 0.00042 | 0.0025 |
| P13473\|LAMP2_HUMAN | -1.441 | 0.00043 | 0.0025 |
| P00558\|PGK1_HUMAN | 1.455 | 0.00045 | 0.0026 |
| Q6P4A8\|PLBL1_HUMAN | -1.181 | 0.00053 | 0.0031 |
| P07360\|CO8G_HUMAN | 0.759 | 0.00054 | 0.0031 |
| P19827\|ITIH1_HUMAN | 1.379 | 0.00055 | 0.0032 |
| P13647\|K2C5_HUMAN | -3.543 | 0.00059 | 0.0033 |
| P62328\|TYB4_HUMAN | -1.432 | 0.00060 | 0.0034 |
| O95171\|SCEL_HUMAN | -2.513 | 0.00061 | 0.0034 |
| P22528\|SPR1B_HUMAN | -2.504 | 0.00064 | 0.0035 |
| P20962\|PTMS_HUMAN | -1.569 | 0.00070 | 0.0038 |
| O00391\|QSOX1_HUMAN | -1.601 | 0.00070 | 0.0038 |
| P61916\|NPC2_HUMAN | -1.004 | 0.00075 | 0.0041 |
| P02545\|LMNA_HUMAN | -0.974 | 0.00078 | 0.0041 |
| P61626\|LYSC_HUMAN | -1.296 | 0.00077 | 0.0041 |
| P30050\|RL12_HUMAN | -1.427 | 0.00085 | 0.0044 |
| O43866\|CD5L_HUMAN | 1.130 | 0.00087 | 0.0045 |
| Q9BW04\|SARG_HUMAN | -1.636 | 0.0010 | 0.0053 |
| P35527\|K1C9_HUMAN | -2.069 | 0.0011 | 0.0054 |
| P35321\|SPR1A_HUMAN | -1.791 | 0.0011 | 0.0056 |
| Q9NQ38\|ISK5_HUMAN | -1.352 | 0.0011 | 0.0056 |
| P12830\|CADH1_HUMAN | -0.943 | 0.0013 | 0.0062 |
| P07108\|ACBP_HUMAN | -1.145 | 0.0013 | 0.0062 |
| Q14134\|TRI29_HUMAN | -1.952 | 0.0013 | 0.0064 |
| P17213\|BPI_HUMAN | -1.071 | 0.0014 | 0.0067 |
| P05783\|K1C18_HUMAN | 1.414 | 0.0014 | 0.0067 |
| P30101\|PDIA3_HUMAN | 3.396 | 0.0015 | 0.0070 |
| P08670\|VIME_HUMAN | 1.223 | 0.0015 | 0.0073 |
| P02649\|APOE_HUMAN | 1.179 | 0.0017 | 0.0079 |
| O00515\|LAD1_HUMAN | -1.918 | 0.0019 | 0.0088 |
| P0C0L4\|CO4A_HUMAN | 1.305 | 0.0020 | 0.0092 |
| P32320\|CDD_HUMAN | -1.165 | 0.0020 | 0.0093 |
| P17936\|IBP3_HUMAN | 1.088 | 0.0021 | 0.0098 |
| Q13510\|ASAH1_HUMAN | -0.811 | 0.0023 | 0.010 |
| Q6WKZ4\|RFIP1_HUMAN | -1.653 | 0.0023 | 0.010 |
| P12429\|ANXA3_HUMAN | -0.959 | 0.0024 | 0.011 |
| P02747\|C1QC_HUMAN | 1.126 | 0.0024 | 0.011 |
| P07195\|LDHB_HUMAN | 0.961 | 0.0024 | 0.011 |
| O95274\|LYPD3_HUMAN | -1.618 | 0.0025 | 0.011 |
| P62805\|H4_HUMAN | 1.934 | 0.0025 | 0.011 |
| Q9HD89\|RETN_HUMAN | -0.945 | 0.0029 | 0.012 |
| P16402\|H13_HUMAN | -1.367 | 0.0029 | 0.013 |
| P04003\|C4BPA_HUMAN | 1.980 | 0.0030 | 0.013 |
| P47914\|RL29_HUMAN | -1.486 | 0.0030 | 0.013 |
| P22792\|CPN2_HUMAN | 0.761 | 0.0033 | 0.014 |
| P30086\|PEBP1_HUMAN | -0.811 | 0.0035 | 0.015 |
| P60174\|TPIS_HUMAN | 1.214 | 0.0037 | 0.015 |
| Q8TEA8\|DTD1_HUMAN | -1.097 | 0.0037 | 0.015 |
| Q9Y2P7\|ZN256_HUMAN | 1.198 | 0.0038 | 0.016 |
| P02042\|HBD_HUMAN | 1.749 | 0.0042 | 0.017 |
| P23396\|RS3_HUMAN | -0.836 | 0.0043 | 0.017 |
| P23142\|FBLN1_HUMAN | 0.785 | 0.0044 | 0.018 |
| P28072\|PSB6_HUMAN | -0.714 | 0.0046 | 0.018 |
| P18510\|IL1RA_HUMAN | -1.209 | 0.0046 | 0.018 |
| P19013\|K2C4_HUMAN | -3.218 | 0.0048 | 0.019 |
| O75369\|FLNB_HUMAN | -1.269 | 0.0050 | 0.019 |
| P05090\|APOD_HUMAN | 0.924 | 0.0051 | 0.020 |
| Q8NE71\|ABCF1_HUMAN | 0.576 | 0.0052 | 0.020 |
| Q99460\|PSMD1_HUMAN | -1.238 | 0.0056 | 0.021 |
| P14174\|MIF_HUMAN | 1.275 | 0.0058 | 0.022 |
| P13671\|CO6_HUMAN | 0.499 | 0.0060 | 0.022 |
| P02750\|A2GL_HUMAN | 0.758 | 0.0063 | 0.024 |
| P01610\|KV118_HUMAN | -1.624 | 0.0064 | 0.024 |
| P37837\|TALDO_HUMAN | -0.726 | 0.0069 | 0.025 |
| P62263\|RS14_HUMAN | -1.296 | 0.0070 | 0.026 |
| P13645\|K1C10_HUMAN | -1.156 | 0.0072 | 0.026 |
| Q9UL52\|TM11E_HUMAN | -1.317 | 0.0073 | 0.027 |
| P01768\|HV307_HUMAN_UNMAPPED | 3.027 | 0.0074 | 0.027 |
| O60635\|TSN1_HUMAN | 0.860 | 0.0075 | 0.027 |
| P04075\|ALDOA_HUMAN | -0.708 | 0.0075 | 0.027 |
| O43175\|SERA_HUMAN | -1.165 | 0.0077 | 0.027 |
| Q8N1N4\|K2C78_HUMAN | -1.457 | 0.0084 | 0.030 |
| P49327\|FAS_HUMAN | 1.194 | 0.0086 | 0.030 |
| P01859\|IGHG2_HUMAN | 0.811 | 0.0088 | 0.031 |
| Q9HCY8\|S10AE_HUMAN | -1.267 | 0.0093 | 0.032 |
| P06748\|NPM_HUMAN | 2.450 | 0.0099 | 0.034 |
| P06744\|G6PI_HUMAN | 0.924 | 0.010 | 0.035 |
| P45880\|VDAC2_HUMAN | 1.758 | 0.011 | 0.036 |
| P19652\|A1AG2_HUMAN | 1.323 | 0.011 | 0.036 |
| P63104\|1433Z_HUMAN | 1.023 | 0.011 | 0.036 |
| Q9BRA2\|TXD17_HUMAN | -0.885 | 0.011 | 0.036 |
| P27816\|MAP4_HUMAN | -1.708 | 0.011 | 0.036 |
| P04259\|K2C6B_HUMAN | -2.343 | 0.013 | 0.042 |
| P02746\|C1QB_HUMAN | 0.772 | 0.013 | 0.042 |
| O15231\|ZN185_HUMAN | -1.941 | 0.013 | 0.042 |
| P30048\|PRDX3_HUMAN | 0.560 | 0.013 | 0.044 |
| Q13126\|MTAP_HUMAN | -0.638 | 0.014 | 0.044 |
| P06727\|APOA4_HUMAN | 1.347 | 0.014 | 0.046 |
| Q12802\|AKP13_HUMAN | -0.707 | 0.015 | 0.047 |
| P61106\|RAB14_HUMAN | -0.996 | 0.015 | 0.048 |
| O75368\|SH3L1_HUMAN | -0.503 | 0.016 | 0.052 |
| P07737\|PROF1_HUMAN | 0.847 | 0.017 | 0.053 |
| P05387\|RLA2_HUMAN | -0.666 | 0.017 | 0.053 |
| P11142\|HSP7C_HUMAN | 0.847 | 0.018 | 0.055 |
| P59998\|ARPC4_HUMAN | 0.796 | 0.019 | 0.057 |
| O75342\|LX12B_HUMAN | 0.579 | 0.019 | 0.057 |
| P01861\|IGHG4_HUMAN | 0.799 | 0.019 | 0.059 |
| P07858\|CATB_HUMAN | -1.191 | 0.020 | 0.060 |
| P01034\|CYTC_HUMAN | 0.632 | 0.020 | 0.060 |
| P23526\|SAHH_HUMAN | -0.791 | 0.020 | 0.060 |
| P54652\|HSP72_HUMAN | -1.539 | 0.021 | 0.062 |
| P68104\|EF1A1_HUMAN | -1.478 | 0.021 | 0.063 |
| P04179\|SODM_HUMAN | 0.881 | 0.024 | 0.072 |
| Q6KB66\|K2C80_HUMAN | -1.272 | 0.026 | 0.077 |
| P19957\|ELAF_HUMAN | -1.538 | 0.027 | 0.080 |
| Q9NZT1\|CALL5_HUMAN | -1.190 | 0.028 | 0.081 |
| O75629\|CREG1_HUMAN | -0.608 | 0.028 | 0.082 |
| Q92820\|GGH_HUMAN | -0.578 | 0.028 | 0.082 |
| P35221\|CTNA1_HUMAN | -1.120 | 0.028 | 0.082 |
| Q13835\|PKP1_HUMAN | -0.708 | 0.029 | 0.084 |
| P09651\|ROA1_HUMAN | -1.983 | 0.030 | 0.085 |
| P02656\|APOC3_HUMAN | -1.191 | 0.030 | 0.086 |
| Q6UX06\|OLFM4_HUMAN | 0.877 | 0.031 | 0.087 |
| P62917\|RL8_HUMAN | -1.017 | 0.031 | 0.088 |
| Q6E0U4\|DMKN_HUMAN | -1.444 | 0.032 | 0.090 |
| P36952\|SPB5_HUMAN | 3.088 | 0.033 | 0.091 |
| P06737\|PYGL_HUMAN | 1.327 | 0.033 | 0.093 |
| P62424\|RL7A_HUMAN | -1.666 | 0.034 | 0.094 |
| P22626\|ROA2_HUMAN | -0.750 | 0.035 | 0.096 |
| P08107\|HSP71_HUMAN | 0.754 | 0.036 | 0.097 |
| P01594\|KV102_HUMAN | -0.409 | 0.036 | 0.099 |
| P22894\|MMP8_HUMAN | -0.736 | 0.036 | 0.099 |
| P61019\|RAB2A_HUMAN | -0.713 | 0.038 | 0.10 |
| P05543\|THBG_HUMAN | -0.688 | 0.040 | 0.11 |
| Q13867\|BLMH_HUMAN | -0.614 | 0.040 | 0.11 |
| P16070\|CD44_HUMAN | 0.347 | 0.040 | 0.11 |
| Q9ULC6\|PADI1_HUMAN | -0.852 | 0.041 | 0.11 |
| Q15843\|NEDD8_HUMAN | -0.745 | 0.042 | 0.11 |
| P35326\|SPR2A_HUMAN | -1.908 | 0.042 | 0.11 |
| P61769\|B2MG_HUMAN | 0.608 | 0.043 | 0.11 |
| P36955\|PEDF_HUMAN | 0.545 | 0.043 | 0.11 |
| P25788\|PSA3_HUMAN | -0.417 | 0.044 | 0.11 |
| P12035\|K2C3_HUMAN | -1.407 | 0.044 | 0.11 |
| P26038\|MOES_HUMAN | -0.468 | 0.045 | 0.12 |
| Q14002\|CEAM7_HUMAN | -0.798 | 0.045 | 0.12 |
| P15941\|MUC1_HUMAN | 0.807 | 0.045 | 0.12 |
| P07437\|TBB5_HUMAN | -0.701 | 0.046 | 0.12 |
| P00739\|HPTR_HUMAN | -1.132 | 0.048 | 0.12 |
| P02538\|K2C6A_HUMAN | -2.689 | 0.048 | 0.12 |
| P02654\|APOC1_HUMAN | 0.872 | 0.048 | 0.12 |
| P10153\|RNAS2_HUMAN | -0.691 | 0.048 | 0.12 |
| O43240\|KLK10_HUMAN | -1.963 | 0.049 | 0.12 |
| P20930\|FILA_HUMAN | -1.145 | 0.049 | 0.12 |
| O15143\|ARC1B_HUMAN | -0.593 | 0.050 | 0.12 |
| P15104\|GLNA_HUMAN | 0.889 | 0.052 | 0.13 |
| P60660\|MYL6_HUMAN | 0.694 | 0.053 | 0.13 |
| P29373\|RABP2_HUMAN | -0.622 | 0.053 | 0.13 |
| Q15181\|IPYR_HUMAN | -0.829 | 0.054 | 0.13 |
| P0CG47\|UBB_HUMAN | -0.959 | 0.054 | 0.13 |
| P07339\|CATD_HUMAN | 0.415 | 0.054 | 0.13 |
| P62258\|1433E_HUMAN | 0.684 | 0.055 | 0.13 |
| Q9NZD2\|GLTP_HUMAN | -0.432 | 0.057 | 0.14 |
| P40121\|CAPG_HUMAN | -0.462 | 0.057 | 0.14 |
| Q92597\|NDRG1_HUMAN | -1.235 | 0.057 | 0.14 |
| P62753\|RS6_HUMAN | -0.948 | 0.058 | 0.14 |
| O15511\|ARPC5_HUMAN | 0.463 | 0.059 | 0.14 |
| P01770\|HV309_HUMAN | 0.343 | 0.059 | 0.14 |
| P01703\|LV105_HUMAN | 0.357 | 0.059 | 0.14 |
| P07384\|CAN1_HUMAN | -0.653 | 0.061 | 0.14 |
| P48594\|SPB4_HUMAN | -0.786 | 0.062 | 0.14 |
| P50991\|TCPD_HUMAN | 0.542 | 0.067 | 0.15 |
| P14550\|AK1A1_HUMAN | -0.711 | 0.067 | 0.15 |
| P01877\|IGHA2_HUMAN | -0.506 | 0.068 | 0.16 |
| P01717\|LV403_HUMAN | 0.342 | 0.070 | 0.16 |
| P37802\|TAGL2_HUMAN | -0.633 | 0.069 | 0.16 |
| P55327\|TPD52_HUMAN | -0.624 | 0.071 | 0.16 |
| P15924\|DESP_HUMAN | -1.232 | 0.074 | 0.17 |
| P06310\|KV206_HUMAN | -0.608 | 0.077 | 0.17 |
| Q02878\|RL6_HUMAN | -1.270 | 0.077 | 0.17 |
| P00491\|PNPH_HUMAN | 0.573 | 0.078 | 0.17 |
| P04220\|MUCB_HUMAN | 0.801 | 0.079 | 0.17 |
| P19367\|HXK1_HUMAN | 0.779 | 0.079 | 0.17 |
| Q9UKR3\|KLK13_HUMAN | -1.045 | 0.079 | 0.17 |
| P06576\|ATPB_HUMAN | 0.714 | 0.080 | 0.18 |
| P09382\|LEG1_HUMAN | -0.655 | 0.083 | 0.18 |
| P20618\|PSB1_HUMAN | -0.699 | 0.083 | 0.18 |
| O60437\|PEPL_HUMAN | -1.795 | 0.084 | 0.18 |
| Q12841\|FSTL1_HUMAN | 1.413 | 0.085 | 0.18 |
| P27824\|CALX_HUMAN | 1.002 | 0.085 | 0.18 |
| Q53FA7\|QORX_HUMAN | -0.702 | 0.086 | 0.19 |
| P06312\|KV401_HUMAN | -0.676 | 0.089 | 0.19 |
| P51884\|LUM_HUMAN | 0.613 | 0.089 | 0.19 |
| P08246\|ELNE_HUMAN | -0.565 | 0.091 | 0.19 |
| P80748\|LV302_HUMAN | 0.316 | 0.093 | 0.20 |
| Q99536\|VAT1_HUMAN | -0.356 | 0.093 | 0.20 |
| O75340\|PDCD6_HUMAN | -0.583 | 0.094 | 0.20 |
| P53004\|BIEA_HUMAN | -0.432 | 0.094 | 0.20 |
| P51858\|HDGF_HUMAN | -0.762 | 0.094 | 0.20 |
| P18054\|LOX12_HUMAN | 0.561 | 0.095 | 0.20 |
| Q16658\|FSCN1_HUMAN | -0.783 | 0.097 | 0.20 |
| P05107\|ITB2_HUMAN | -1.059 | 0.097 | 0.20 |
| P01611\|KV119_HUMAN | 0.274 | 0.10 | 0.21 |
| P52597\|HNRPF_HUMAN | 0.485 | 0.11 | 0.23 |
| Q06033\|ITIH3_HUMAN | 0.926 | 0.11 | 0.23 |
| P62937\|PPIA_HUMAN | 0.650 | 0.11 | 0.23 |
| P13010\|XRCC5_HUMAN | 0.552 | 0.11 | 0.23 |
| P02730\|B3AT_HUMAN | 0.842 | 0.12 | 0.24 |
| P12956\|XRCC6_HUMAN | 0.769 | 0.12 | 0.24 |
| Q96FW1\|OTUB1_HUMAN | -0.512 | 0.12 | 0.24 |
| Q86V81\|THOC4_HUMAN | 1.090 | 0.12 | 0.24 |
| P52209\|6PGD_HUMAN | -0.906 | 0.12 | 0.24 |
| Q07654\|TFF3_HUMAN | -1.251 | 0.13 | 0.25 |
| Q92817\|EVPL_HUMAN | -2.189 | 0.13 | 0.25 |
| P60900\|PSA6_HUMAN | -0.335 | 0.13 | 0.26 |
| P05141\|ADT2_HUMAN | 0.480 | 0.13 | 0.26 |
| P55145\|MANF_HUMAN | -0.957 | 0.13 | 0.26 |
| P01714\|LV301_HUMAN | 0.353 | 0.13 | 0.26 |
| P07225\|PROS_HUMAN | 0.503 | 0.14 | 0.27 |
| Q8TDL5\|BPIB1_HUMAN | -0.532 | 0.14 | 0.27 |
| P61158\|ARP3_HUMAN | -0.297 | 0.14 | 0.28 |
| Q8TE68\|ES8L1_HUMAN | 0.876 | 0.14 | 0.28 |
| P01603\|KV111_HUMAN | -0.730 | 0.15 | 0.28 |
| P01622\|KV304_HUMAN | 0.270 | 0.15 | 0.29 |
| Q8N122\|RPTOR_HUMAN | -1.111 | 0.15 | 0.29 |
| P68366\|TBA4A_HUMAN | -0.556 | 0.16 | 0.30 |
| P0CG05\|LAC2_HUMAN | 0.625 | 0.16 | 0.30 |
| P55058\|PLTP_HUMAN | 0.696 | 0.16 | 0.30 |
| P30044\|PRDX5_HUMAN | 0.495 | 0.16 | 0.30 |
| P09211\|GSTP1_HUMAN | 0.574 | 0.16 | 0.31 |
| P52790\|HXK3_HUMAN | 0.508 | 0.16 | 0.31 |
| Q13231\|CHIT1_HUMAN | -0.659 | 0.16 | 0.31 |
| P26373\|RL13_HUMAN | -1.192 | 0.16 | 0.31 |
| P11215\|ITAM_HUMAN | 0.513 | 0.17 | 0.31 |
| P80511\|S10AC_HUMAN | -0.610 | 0.17 | 0.31 |
| P48595\|SPB10_HUMAN | 0.597 | 0.17 | 0.31 |
| Q16831\|UPP1_HUMAN | 0.460 | 0.17 | 0.31 |
| P52943\|CRIP2_HUMAN | -0.682 | 0.17 | 0.32 |
| O75874\|IDHC_HUMAN | 0.653 | 0.18 | 0.32 |
| P18669\|PGAM1_HUMAN | -0.636 | 0.18 | 0.32 |
| P00338\|LDHA_HUMAN | 0.344 | 0.18 | 0.32 |
| P32119\|PRDX2_HUMAN | 0.477 | 0.18 | 0.33 |
| P02511\|CRYAB_HUMAN | -1.458 | 0.18 | 0.33 |
| O75531\|BAF_HUMAN | -0.329 | 0.18 | 0.33 |
| P47929\|LEG7_HUMAN | 0.884 | 0.19 | 0.33 |
| O60888\|CUTA_HUMAN | 0.341 | 0.19 | 0.33 |
| P04430\|KV122_HUMAN | -0.294 | 0.19 | 0.34 |
| P26641\|EF1G_HUMAN | 0.506 | 0.19 | 0.34 |
| O75594\|PGRP1_HUMAN | -0.387 | 0.19 | 0.34 |
| P08779\|K1C16_HUMAN | -1.254 | 0.19 | 0.34 |
| Q15084\|PDIA6_HUMAN | -0.413 | 0.19 | 0.34 |
| P47756\|CAPZB_HUMAN | -0.361 | 0.20 | 0.35 |
| P13797\|PLST_HUMAN | -0.546 | 0.20 | 0.35 |
| P05386\|RLA1_HUMAN | 0.422 | 0.20 | 0.35 |
| P25815\|S100P_HUMAN | -0.372 | 0.21 | 0.35 |
| Q16651\|PRSS8_HUMAN | -0.428 | 0.20 | 0.35 |
| Q6ZVX7\|FBX50_HUMAN | -0.431 | 0.20 | 0.35 |
| P50990\|TCPQ_HUMAN | -0.860 | 0.20 | 0.35 |
| P01764\|HV303_HUMAN | -1.042 | 0.21 | 0.36 |
| P01700\|LV102_HUMAN | -0.303 | 0.21 | 0.36 |
| P24158\|PRTN3_HUMAN | -0.520 | 0.21 | 0.37 |
| P00748\|FA12_HUMAN | 0.543 | 0.22 | 0.37 |
| P68363\|TBA1B_HUMAN | -0.425 | 0.22 | 0.37 |
| P27482\|CALL3_HUMAN | -0.445 | 0.22 | 0.37 |
| P06331\|HV209_HUMAN | 0.183 | 0.22 | 0.37 |
| P25787\|PSA2_HUMAN | -0.325 | 0.22 | 0.37 |
| P31997\|CEAM8_HUMAN | -0.873 | 0.22 | 0.37 |
| Q9Y5Z4\|HEBP2_HUMAN | -0.321 | 0.22 | 0.37 |
| Q6UXB3\|LYPD2_HUMAN | -0.600 | 0.22 | 0.38 |
| P49368\|TCPG_HUMAN | 0.604 | 0.23 | 0.38 |
| P14625\|ENPL_HUMAN | 0.359 | 0.23 | 0.38 |
| O60814\|H2B1K_HUMAN | 0.517 | 0.23 | 0.38 |
| P01834\|IGKC_HUMAN | 0.266 | 0.23 | 0.38 |
| P27169\|PON1_HUMAN | 0.495 | 0.23 | 0.39 |
| P01780\|HV319_HUMAN | -0.455 | 0.23 | 0.39 |
| P00390\|GSHR_HUMAN | -0.312 | 0.24 | 0.39 |
| P12955\|PEPD_HUMAN | -0.510 | 0.24 | 0.39 |
| P34932\|HSP74_HUMAN | -0.373 | 0.24 | 0.40 |
| Q96DA0\|ZG16B_HUMAN | -0.421 | 0.25 | 0.40 |
| P11413\|G6PD_HUMAN | -0.342 | 0.25 | 0.40 |
| P07357\|CO8A_HUMAN | 0.281 | 0.25 | 0.41 |
| Q9H299\|SH3L3_HUMAN | -0.418 | 0.25 | 0.41 |
| P22234\|PUR6_HUMAN | -0.297 | 0.25 | 0.41 |
| P02655\|APOC2_HUMAN | 0.721 | 0.26 | 0.41 |
| P26583\|HMGB2_HUMAN | -0.329 | 0.26 | 0.42 |
| P01602\|KV110_HUMAN | 0.586 | 0.26 | 0.42 |
| Q9UN36\|NDRG2_HUMAN | -0.366 | 0.26 | 0.42 |
| Q02413\|DSG1_HUMAN | -0.492 | 0.26 | 0.42 |
| P36871\|PGM1_HUMAN | -0.827 | 0.26 | 0.42 |
| Q6ZMR5\|TM11A_HUMAN | -0.431 | 0.27 | 0.42 |
| Q9BQE3\|TBA1C_HUMAN | -0.327 | 0.27 | 0.42 |
| P23528\|COF1_HUMAN | 0.283 | 0.27 | 0.42 |
| Q9Y2V2\|CHSP1_HUMAN | -0.416 | 0.27 | 0.42 |
| P13639\|EF2_HUMAN | 0.409 | 0.27 | 0.43 |
| Q7Z406\|MYH14_HUMAN | 0.785 | 0.28 | 0.43 |
| Q9Y3Q3\|TMED3_HUMAN | 0.406 | 0.28 | 0.43 |
| Q06830\|PRDX1_HUMAN | 0.271 | 0.28 | 0.43 |
| P30041\|PRDX6_HUMAN | -0.257 | 0.28 | 0.43 |
| Q96C19\|EFHD2_HUMAN | -0.304 | 0.28 | 0.43 |
| Q86T26\|TM11B_HUMAN | -0.407 | 0.28 | 0.43 |
| P17174\|AATC_HUMAN | -0.455 | 0.28 | 0.43 |
| P06311\|KV311_HUMAN | -0.343 | 0.28 | 0.44 |
| O43707\|ACTN4_HUMAN | 0.495 | 0.29 | 0.44 |
| P07237\|PDIA1_HUMAN | 0.310 | 0.29 | 0.45 |
| Q5D862\|FILA2_HUMAN | -0.317 | 0.30 | 0.45 |
| P15153\|RAC2_HUMAN | -0.342 | 0.30 | 0.45 |
| P08185\|CBG_HUMAN | 0.371 | 0.30 | 0.46 |
| P04208\|LV106_HUMAN | 0.283 | 0.30 | 0.46 |
| P01596\|KV104_HUMAN | -0.531 | 0.30 | 0.46 |
| P48637\|GSHB_HUMAN | -0.291 | 0.31 | 0.46 |
| P62826\|RAN_HUMAN | -0.429 | 0.31 | 0.47 |
| Q96PD5\|PGRP2_HUMAN | 0.697 | 0.31 | 0.47 |
| P08865\|RSSA_HUMAN | -0.306 | 0.31 | 0.47 |
| Q13263\|TIF1B_HUMAN | 0.685 | 0.32 | 0.47 |
| P09525\|ANXA4_HUMAN | -0.358 | 0.32 | 0.47 |
| P25789\|PSA4_HUMAN | -0.263 | 0.32 | 0.48 |
| P31944\|CASPE_HUMAN | -0.320 | 0.32 | 0.48 |
| Q9UHA7\|IL36A_HUMAN | -0.588 | 0.33 | 0.48 |
| P17987\|TCPA_HUMAN | 0.681 | 0.33 | 0.49 |
| A0AV96\|RBM47_HUMAN | 0.234 | 0.34 | 0.49 |
| P35579\|MYH9_HUMAN | 0.430 | 0.34 | 0.50 |
| P00918\|CAH2_HUMAN | 0.509 | 0.35 | 0.50 |
| P29622\|KAIN_HUMAN | 0.456 | 0.36 | 0.52 |
| P03952\|KLKB1_HUMAN | 0.286 | 0.36 | 0.52 |
| P57735\|RAB25_HUMAN | 0.857 | 0.36 | 0.52 |
| P05164\|PERM_HUMAN | -0.392 | 0.36 | 0.53 |
| Q9ULD9\|ZN608_HUMAN | -0.276 | 0.38 | 0.54 |
| P60709\|ACTB_HUMAN | -0.377 | 0.38 | 0.54 |
| P09467\|F16P1_HUMAN | -0.446 | 0.38 | 0.54 |
| P08758\|ANXA5_HUMAN | -0.492 | 0.38 | 0.55 |
| P01880\|IGHD_HUMAN | 0.425 | 0.39 | 0.56 |
| P47755\|CAZA2_HUMAN | 0.295 | 0.39 | 0.56 |
| P09960\|LKHA4_HUMAN | 0.273 | 0.39 | 0.56 |
| P62081\|RS7_HUMAN | 0.297 | 0.40 | 0.56 |
| P01620\|KV302_HUMAN | -0.208 | 0.40 | 0.56 |
| P01106\|MYC_HUMAN | -0.531 | 0.40 | 0.56 |
| Q9Y490\|TLN1_HUMAN | -0.285 | 0.40 | 0.56 |
| P33992\|MCM5_HUMAN | 0.363 | 0.41 | 0.57 |
| P06753\|TPM3_HUMAN | -0.271 | 0.41 | 0.57 |
| P15311\|EZRI_HUMAN | -0.274 | 0.41 | 0.57 |
| Q8WUM4\|PDC6I_HUMAN | -0.371 | 0.41 | 0.57 |
| P04080\|CYTB_HUMAN | -0.393 | 0.41 | 0.57 |
| P04406\|G3P_HUMAN | -0.357 | 0.41 | 0.57 |
| P04792\|HSPB1_HUMAN | -0.404 | 0.42 | 0.58 |
| O15144\|ARPC2_HUMAN | 0.414 | 0.42 | 0.58 |
| P11279\|LAMP1_HUMAN | -0.403 | 0.42 | 0.58 |
| Q9UJ70\|NAGK_HUMAN | -0.323 | 0.42 | 0.58 |
| Q01518\|CAP1_HUMAN | 0.258 | 0.43 | 0.58 |
| P28066\|PSA5_HUMAN | -0.244 | 0.43 | 0.58 |
| P22314\|UBA1_HUMAN | 0.271 | 0.43 | 0.59 |
| Q9P1F3\|ABRAL_HUMAN | -0.228 | 0.44 | 0.60 |
| Q9H4A4\|AMPB_HUMAN | -0.475 | 0.44 | 0.60 |
| P04207\|KV308_HUMAN | 0.484 | 0.44 | 0.60 |
| P55072\|TERA_HUMAN | -0.273 | 0.45 | 0.60 |
| Q6XQN6\|PNCB_HUMAN | -0.314 | 0.45 | 0.60 |
| P23246\|SFPQ_HUMAN | -0.589 | 0.45 | 0.60 |
| P16152\|CBR1_HUMAN | -0.696 | 0.45 | 0.60 |
| P80723\|BASP1_HUMAN | -0.301 | 0.45 | 0.60 |
| Q01130\|SRSF2_HUMAN | -0.293 | 0.46 | 0.61 |
| P35268\|RL22_HUMAN | -0.380 | 0.46 | 0.62 |
| P18135\|KV312_HUMAN | -0.390 | 0.47 | 0.62 |
| Q96HE7\|ERO1A_HUMAN | -0.283 | 0.47 | 0.62 |
| P05787\|K2C8_HUMAN | 0.960 | 0.47 | 0.63 |
| P43251\|BTD_HUMAN | 0.156 | 0.51 | 0.68 |
| Q07812\|BAX_HUMAN | -0.195 | 0.51 | 0.68 |
| P01766\|HV305_HUMAN | -0.113 | 0.52 | 0.69 |
| P20160\|CAP7_HUMAN | -0.199 | 0.52 | 0.69 |
| Q7Z4W1\|DCXR_HUMAN | -0.183 | 0.52 | 0.69 |
| P05556\|ITB1_HUMAN | 0.199 | 0.53 | 0.69 |
| P01743\|HV102_HUMAN | 0.112 | 0.53 | 0.69 |
| Q9BRF8\|CPPED_HUMAN | -0.199 | 0.53 | 0.69 |
| P12111\|CO6A3_HUMAN | -0.216 | 0.53 | 0.69 |
| P10643\|CO7_HUMAN | 0.202 | 0.53 | 0.69 |
| P02652\|APOA2_HUMAN | 0.329 | 0.54 | 0.69 |
| P0C0L5\|CO4B_HUMAN | 0.328 | 0.54 | 0.69 |
| O75556\|SG2A1_HUMAN | -0.118 | 0.54 | 0.69 |
| O60664\|PLIN3_HUMAN | -0.413 | 0.54 | 0.69 |
| P14780\|MMP9_HUMAN | 0.249 | 0.54 | 0.69 |
| O14745\|NHRF1_HUMAN | 0.188 | 0.54 | 0.69 |
| P49720\|PSB3_HUMAN | -0.170 | 0.54 | 0.69 |
| P67936\|TPM4_HUMAN | -0.196 | 0.54 | 0.69 |
| P04908\|H2A1B_HUMAN | 0.188 | 0.55 | 0.70 |
| Q6XPR3\|RPTN_HUMAN | -0.621 | 0.55 | 0.70 |
| P40925\|MDHC_HUMAN | 0.196 | 0.56 | 0.71 |
| P36222\|CH3L1_HUMAN | -0.150 | 0.57 | 0.72 |
| P62942\|FKB1A_HUMAN | -0.193 | 0.57 | 0.72 |
| P63261\|ACTG_HUMAN | -0.238 | 0.57 | 0.72 |
| P12273\|PIP_HUMAN | -0.297 | 0.57 | 0.72 |
| O43768\|ENSA_HUMAN | 0.370 | 0.58 | 0.73 |
| P05089\|ARGI1_HUMAN | 0.212 | 0.59 | 0.73 |
| P61978\|HNRPK_HUMAN | -0.148 | 0.59 | 0.73 |
| P21333\|FLNA_HUMAN | 0.207 | 0.60 | 0.74 |
| P01762\|HV301_HUMAN | -0.077 | 0.60 | 0.74 |
| P68036\|UB2L3_HUMAN | -0.140 | 0.60 | 0.74 |
| P06454\|PTMA_HUMAN | -0.172 | 0.60 | 0.74 |
| P48735\|IDHP_HUMAN | -0.180 | 0.60 | 0.74 |
| P06681\|CO2_HUMAN | -0.211 | 0.60 | 0.74 |
| P05120\|PAI2_HUMAN | -0.245 | 0.61 | 0.75 |
| P55786\|PSA_HUMAN | 0.327 | 0.61 | 0.75 |
| P08519\|APOA_HUMAN | 0.268 | 0.62 | 0.76 |
| P21926\|CD9_HUMAN | 0.163 | 0.62 | 0.76 |
| P09871\|C1S_HUMAN | 0.202 | 0.64 | 0.77 |
| Q04917\|1433F_HUMAN | 0.191 | 0.63 | 0.77 |
| Q00610\|CLH1_HUMAN | 0.189 | 0.63 | 0.77 |
| Q13228\|SBP1_HUMAN | 0.156 | 0.64 | 0.77 |
| P50395\|GDIB_HUMAN | 0.152 | 0.64 | 0.77 |
| P27348\|1433T_HUMAN | 0.149 | 0.64 | 0.77 |
| P01625\|KV402_HUMAN | -0.062 | 0.63 | 0.77 |
| P01781\|HV320_HUMAN | -0.099 | 0.64 | 0.77 |
| P20061\|TCO1_HUMAN | -0.127 | 0.63 | 0.77 |
| O60218\|AK1BA_HUMAN | -0.209 | 0.63 | 0.77 |
| P14317\|HCLS1_HUMAN | -0.237 | 0.65 | 0.77 |
| P00915\|CAH1_HUMAN | 0.280 | 0.65 | 0.78 |
| P60842\|IF4A1_HUMAN | 0.162 | 0.65 | 0.78 |
| P41218\|MNDA_HUMAN | 0.142 | 0.66 | 0.78 |
| P01597\|KV105_HUMAN_UNMAPPED | -0.117 | 0.66 | 0.78 |
| P23381\|SYWC_HUMAN | -0.123 | 0.66 | 0.78 |
| Q14764\|MVP_HUMAN | 0.294 | 0.66 | 0.78 |
| P01624\|KV306_HUMAN | -0.105 | 0.66 | 0.78 |
| P01619\|KV301_HUMAN | 0.208 | 0.67 | 0.79 |
| O00299\|CLIC1_HUMAN | 0.132 | 0.67 | 0.79 |
| P12724\|ECP_HUMAN | 0.226 | 0.68 | 0.79 |
| P52565\|GDIR1_HUMAN | -0.105 | 0.68 | 0.79 |
| P01597\|KV105_HUMAN | -0.156 | 0.68 | 0.79 |
| P30043\|BLVRB_HUMAN | 0.160 | 0.68 | 0.79 |
| O14818\|PSA7_HUMAN | -0.136 | 0.68 | 0.79 |
| P01772\|HV311_HUMAN | -0.143 | 0.69 | 0.80 |
| O14880\|MGST3_HUMAN | 0.160 | 0.70 | 0.80 |
| P43490\|NAMPT_HUMAN | 0.132 | 0.70 | 0.80 |
| P17931\|LEG3_HUMAN | 0.123 | 0.70 | 0.80 |
| P08311\|CATG_HUMAN | 0.102 | 0.69 | 0.80 |
| P01857\|IGHG1_HUMAN | 0.090 | 0.70 | 0.80 |
| O75347\|TBCA_HUMAN | -0.152 | 0.69 | 0.80 |
| P60953\|CDC42_HUMAN | -0.147 | 0.70 | 0.81 |
| Q14974\|IMB1_HUMAN | -0.096 | 0.71 | 0.81 |
| Q96AE4\|FUBP1_HUMAN | 0.192 | 0.71 | 0.81 |
| P01767\|HV306_HUMAN | -0.295 | 0.71 | 0.81 |
| P61981\|1433G_HUMAN | -0.110 | 0.72 | 0.81 |
| P01601\|KV109_HUMAN | -0.068 | 0.73 | 0.82 |
| P49913\|CAMP_HUMAN | -0.141 | 0.73 | 0.83 |
| O14791\|APOL1_HUMAN | 0.124 | 0.73 | 0.83 |
| Q13751\|LAMB3_HUMAN | 0.168 | 0.73 | 0.83 |
| P01598\|KV106_HUMAN | -0.062 | 0.74 | 0.83 |
| P62318\|SMD3_HUMAN | -0.097 | 0.74 | 0.83 |
| Q15847\|ADIRF_HUMAN | -0.294 | 0.74 | 0.83 |
| P46781\|RS9_HUMAN | 0.160 | 0.75 | 0.84 |
| P49419\|AL7A1_HUMAN | -0.140 | 0.75 | 0.84 |
| Q9UBD6\|RHCG_HUMAN | -0.160 | 0.75 | 0.84 |
| P04433\|KV309_HUMAN | -0.059 | 0.76 | 0.84 |
| Q96G03\|PGM2_HUMAN | -0.135 | 0.76 | 0.84 |
| O43760\|SNG2_HUMAN | -0.089 | 0.76 | 0.84 |
| P23284\|PPIB_HUMAN | -0.066 | 0.77 | 0.85 |
| O60361\|NDK8_HUMAN | -0.223 | 0.77 | 0.85 |
| P01876\|IGHA1_HUMAN | -0.064 | 0.78 | 0.87 |
| P01833\|PIGR_HUMAN | -0.100 | 0.79 | 0.87 |
| P01765\|HV304_HUMAN | -0.047 | 0.80 | 0.87 |
| Q9NUQ9\|FA49B_HUMAN | -0.095 | 0.80 | 0.88 |
| P52566\|GDIR2_HUMAN | -0.086 | 0.80 | 0.88 |
| Q14103\|HNRPD_HUMAN | -0.065 | 0.81 | 0.88 |
| P37108\|SRP14_HUMAN | -0.084 | 0.81 | 0.88 |
| O75882\|ATRN_HUMAN | -0.071 | 0.81 | 0.88 |
| P46776\|RL27A_HUMAN | -0.096 | 0.81 | 0.88 |
| P49189\|AL9A1_HUMAN | -0.107 | 0.82 | 0.89 |
| P00505\|AATM_HUMAN | 0.082 | 0.82 | 0.89 |
| P01591\|IGJ_HUMAN | -0.065 | 0.82 | 0.89 |
| Q9HC84\|MUC5B_HUMAN | -0.098 | 0.82 | 0.89 |
| P29034\|S10A2_HUMAN | -0.115 | 0.83 | 0.90 |
| P25705\|ATPA_HUMAN | 0.105 | 0.83 | 0.90 |
| Q14019\|COTL1_HUMAN | -0.074 | 0.84 | 0.90 |
| P08697\|A2AP_HUMAN | 0.120 | 0.84 | 0.90 |
| P01763\|HV302_HUMAN | -0.029 | 0.85 | 0.91 |
| P13796\|PLSL_HUMAN | -0.068 | 0.85 | 0.91 |
| P02788\|TRFL_HUMAN | -0.070 | 0.85 | 0.91 |
| P01701\|LV103_HUMAN | -0.029 | 0.86 | 0.91 |
| P35908\|K22E_HUMAN | 0.086 | 0.87 | 0.91 |
| P40926\|MDHM_HUMAN | 0.043 | 0.87 | 0.91 |
| P01617\|KV204_HUMAN | 0.036 | 0.86 | 0.91 |
| P04899\|GNAI2_HUMAN | -0.042 | 0.86 | 0.91 |
| Q99497\|PARK7_HUMAN | -0.065 | 0.87 | 0.91 |
| P63244\|GBLP_HUMAN | -0.081 | 0.86 | 0.91 |
| P09429\|HMGB1_HUMAN | -0.044 | 0.87 | 0.92 |
| P20810\|ICAL_HUMAN | 0.131 | 0.88 | 0.92 |
| P60903\|S10AA_HUMAN | 0.130 | 0.88 | 0.92 |
| P13489\|RINI_HUMAN | -0.046 | 0.88 | 0.92 |
| Q99102\|MUC4_HUMAN | 0.072 | 0.89 | 0.93 |
| P04839\|CY24B_HUMAN | -0.047 | 0.90 | 0.93 |
| P31146\|COR1A_HUMAN | -0.048 | 0.90 | 0.93 |
| Q9UGM3\|DMBT1_HUMAN | -0.049 | 0.90 | 0.93 |
| P27797\|CALR_HUMAN | -0.056 | 0.90 | 0.93 |
| P01742\|HV101_HUMAN | -0.032 | 0.91 | 0.94 |
| P09758\|TACD2_HUMAN | -0.056 | 0.92 | 0.95 |
| Q05315\|LEG10_HUMAN | -0.014 | 0.93 | 0.95 |
| P49411\|EFTU_HUMAN | -0.060 | 0.93 | 0.96 |
| P09972\|ALDOC_HUMAN | 0.020 | 0.94 | 0.97 |
| P62158\|CALM_HUMAN | 0.020 | 0.96 | 0.98 |
| P27695\|APEX1_HUMAN | -0.024 | 0.96 | 0.98 |
| Q8NFJ5\|RAI3_HUMAN | -0.014 | 0.96 | 0.98 |
| P01609\|KV117_HUMAN | -0.014 | 0.96 | 0.98 |
| P51149\|RAB7A_HUMAN | 0.014 | 0.97 | 0.98 |
| P46940\|IQGA1_HUMAN | -0.015 | 0.97 | 0.98 |
| P50995\|ANX11_HUMAN | -0.017 | 0.97 | 0.98 |
| P04040\|CATA_HUMAN | 0.012 | 0.97 | 0.98 |
| Q07020\|RL18_HUMAN | 0.009 | 0.98 | 0.98 |
| P19971\|TYPH_HUMAN | -0.012 | 0.97 | 0.98 |
| Q14508\|WFDC2_HUMAN | 0.012 | 0.98 | 0.98 |
| Q96KP4\|CNDP2_HUMAN | 0.007 | 0.98 | 0.99 |
| P61026\|RAB10_HUMAN | 0.006 | 0.98 | 0.99 |
| P01621\|KV303_HUMAN | -0.004 | 0.99 | 0.99 |
| O75367\|H2AY_HUMAN | -0.008 | 0.99 | 0.99 |
| P06733\|ENOA_HUMAN | -0.004 | 0.99 | 0.99 |

**Supplementary Table S2**: List of identified proteins and their log2 fold change values comparing cases and controls in cervico-vaginal fluid cell pellet/debris samples with unadjusted and adjusted p values (Benjamini–Hochberg correction method).

| **Proteins** | **LogFC** | **P-value** | **adj.P.Val** |  |
| --- | --- | --- | --- | --- |
| P04217\|A1BG_HUMAN | 1.677 | 0.00040 | 0.076 |  |
| P69905\|HBA_HUMAN | 1.706 | 0.00063 | 0.076 |  |
| P19957\|ELAF_HUMAN | -1.392 | 0.00073 | 0.076 |  |
| P31944\|CASPE_HUMAN | -2.344 | 0.0030 | 0.17 |  |
| P68871\|HBB_HUMAN | 1.935 | 0.0032 | 0.17 |  |
| P14174\|MIF_HUMAN | 0.993 | 0.0040 | 0.17 |  |
| P00558\|PGK1_HUMAN | 1.226 | 0.0042 | 0.17 |  |
| P68363\|TBA1B_HUMAN | 1.296 | 0.0050 | 0.17 |  |
| P25705\|ATPA_HUMAN | 1.063 | 0.0051 | 0.17 |  |
| P13646\|K1C13_HUMAN | -1.677 | 0.0071 | 0.22 |  |
| P14618\|KPYM_HUMAN | 1.432 | 0.0088 | 0.22 |  |
| P16402\|H13_HUMAN | 1.050 | 0.0092 | 0.22 |  |
| P02042\|HBD_HUMAN | 3.474 | 0.0098 | 0.22 |  |
| P23528\|COF1_HUMAN | 0.696 | 0.010 | 0.22 |  |
| P04083\|ANXA1_HUMAN | -0.709 | 0.014 | 0.25 |  |
| P23246\|SFPQ_HUMAN | 1.756 | 0.015 | 0.25 |  |
| P10412\|H14_HUMAN | 0.996 | 0.015 | 0.25 |  |
| Q06830\|PRDX1_HUMAN | 0.767 | 0.016 | 0.25 |  |
| Q14210\|LY6D_HUMAN | -0.871 | 0.017 | 0.25 |  |
| P05787\|K2C8_HUMAN | 1.906 | 0.017 | 0.25 |  |
| P04406\|G3P_HUMAN | 1.280 | 0.017 | 0.25 |  |
| P15311\|EZRI_HUMAN | -1.133 | 0.019 | 0.27 |  |
| P32119\|PRDX2_HUMAN | -1.389 | 0.022 | 0.30 |  |
| P16403\|H12_HUMAN | 0.953 | 0.023 | 0.30 |  |
| P23142\|FBLN1_HUMAN | 2.040 | 0.024 | 0.30 |  |
| Q15847\|ADIRF_HUMAN | 1.347 | 0.029 | 0.35 |  |
| P19012\|K1C15_HUMAN | -1.063 | 0.031 | 0.35 |  |
| P19823\|ITIH2_HUMAN | -1.242 | 0.032 | 0.35 |  |
| P00505\|AATM_HUMAN | 1.194 | 0.034 | 0.37 |  |
| P80188\|NGAL_HUMAN | -0.678 | 0.037 | 0.38 |  |
| O60437\|PEPL_HUMAN | 0.605 | 0.038 | 0.38 |  |
| P06753\|TPM3_HUMAN | -1.111 | 0.040 | 0.38 |  |
| P05109\|S10A8_HUMAN | -0.751 | 0.041 | 0.38 |  |
| P32926\|DSG3_HUMAN | -1.259 | 0.044 | 0.38 |  |
| Q9HC84\|MUC5B_HUMAN | -1.016 | 0.044 | 0.38 |  |
| P63261\|ACTG_HUMAN | 1.100 | 0.044 | 0.38 |  |
| Q01469\|FABP5_HUMAN | -1.062 | 0.047 | 0.39 |  |
| Q16610\|ECM1_HUMAN | -0.712 | 0.048 | 0.39 |  |
| Q86U42\|PABP2_HUMAN | 0.778 | 0.050 | 0.40 |  |
| P06744\|G6PI_HUMAN | -0.920 | 0.052 | 0.40 |  |
| P35321\|SPR1A_HUMAN | -1.876 | 0.055 | 0.40 |  |
| P62158\|CALM_HUMAN | -0.541 | 0.056 | 0.40 |  |
| P22735\|TGM1_HUMAN | -0.824 | 0.057 | 0.40 |  |
| P11021\|GRP78_HUMAN | -1.751 | 0.057 | 0.40 |  |
| P04259\|K2C6B_HUMAN | -1.067 | 0.057 | 0.40 |  |
| Q8N122\|RPTOR_HUMAN | -0.980 | 0.061 | 0.40 |  |
| O60235\|TM11D_HUMAN | -1.054 | 0.061 | 0.40 |  |
| P02774\|VTDB_HUMAN | 0.844 | 0.063 | 0.41 |  |
| Q6UX06\|OLFM4_HUMAN | -1.016 | 0.065 | 0.41 |  |
| P07476\|INVO_HUMAN | 0.637 | 0.072 | 0.45 |  |
| Q13263\|TIF1B_HUMAN | 1.214 | 0.076 | 0.46 |  |
| Q96HE7\|ERO1A_HUMAN | 0.698 | 0.080 | 0.48 |  |
| P02790\|HEMO_HUMAN | 0.777 | 0.087 | 0.49 |  |
| P16401\|H15_HUMAN | 0.638 | 0.088 | 0.49 |  |
| P48668\|K2C6C_HUMAN | -1.057 | 0.088 | 0.49 |  |
| P00450\|CERU_HUMAN | -0.542 | 0.089 | 0.49 |  |
| P40926\|MDHM_HUMAN | -0.724 | 0.090 | 0.49 |  |
| P04114\|APOB_HUMAN | 1.729 | 0.093 | 0.49 |  |
| P36952\|SPB5_HUMAN | -0.631 | 0.094 | 0.49 |  |
| P49913\|CAMP_HUMAN | 0.700 | 0.095 | 0.49 |  |
| P62328\|TYB4_HUMAN | 0.661 | 0.097 | 0.49 |  |
| P02788\|TRFL_HUMAN | 0.862 | 0.10 | 0.51 |  |
| P02760\|AMBP_HUMAN | 0.478 | 0.11 | 0.52 |  |
| P08729\|K2C7_HUMAN | -0.935 | 0.11 | 0.52 |  |
| P04839\|CY24B_HUMAN | 0.538 | 0.11 | 0.54 |  |
| P07737\|PROF1_HUMAN | 0.516 | 0.12 | 0.56 |  |
| Q9HCY8\|S10AE_HUMAN | -0.472 | 0.13 | 0.59 |  |
| P35908\|K22E_HUMAN | 0.801 | 0.13 | 0.59 |  |
| P02545\|LMNA_HUMAN | 0.459 | 0.13 | 0.59 |  |
| P09211\|GSTP1_HUMAN | 0.537 | 0.14 | 0.59 |  |
| P59998\|ARPC4_HUMAN | 0.562 | 0.14 | 0.59 |  |
| P30043\|BLVRB_HUMAN | 1.659 | 0.14 | 0.59 |  |
| P30101\|PDIA3_HUMAN | 0.701 | 0.14 | 0.59 |  |
| O15144\|ARPC2_HUMAN | 0.798 | 0.14 | 0.59 |  |
| P60660\|MYL6_HUMAN | 0.448 | 0.14 | 0.60 |  |
| P63104\|1433Z_HUMAN | 0.516 | 0.15 | 0.61 |  |
| P31151\|S10A7_HUMAN | -0.518 | 0.15 | 0.61 |  |
| P46940\|IQGA1_HUMAN | 0.925 | 0.15 | 0.61 |  |
| P04207\|KV308_HUMAN | 0.639 | 0.16 | 0.61 |  |
| Q6ZVX7\|FBX50_HUMAN | 0.502 | 0.16 | 0.61 |  |
| P30740\|ILEU_HUMAN | 0.487 | 0.16 | 0.61 |  |
| P37837\|TALDO_HUMAN | 0.523 | 0.16 | 0.61 |  |
| P25815\|S100P_HUMAN | 0.715 | 0.16 | 0.61 |  |
| A8K2U0\|A2ML1_HUMAN | 1.420 | 0.17 | 0.61 |  |
| P18669\|PGAM1_HUMAN | 0.693 | 0.17 | 0.61 |  |
| P02749\|APOH_HUMAN | 0.485 | 0.17 | 0.61 |  |
| P47929\|LEG7_HUMAN | 0.518 | 0.17 | 0.61 |  |
| P27482\|CALL3_HUMAN | -0.461 | 0.18 | 0.61 |  |
| P07237\|PDIA1_HUMAN | 0.605 | 0.18 | 0.61 |  |
| P02748\|CO9_HUMAN | 0.754 | 0.18 | 0.61 |  |
| Q8TDL5\|BPIB1_HUMAN | -0.772 | 0.18 | 0.61 |  |
| P05164\|PERM_HUMAN | 0.622 | 0.18 | 0.61 |  |
| Q9UBC9\|SPRR3_HUMAN | -0.741 | 0.18 | 0.61 |  |
| P01610\|KV118_HUMAN | -0.573 | 0.18 | 0.61 |  |
| P31997\|CEAM8_HUMAN | 0.746 | 0.19 | 0.62 |  |
| Q9UBG3\|CRNN_HUMAN | 0.506 | 0.19 | 0.62 |  |
| Q14134\|TRI29_HUMAN | -0.858 | 0.20 | 0.63 |  |
| Q8WWA1\|TMM40_HUMAN | -0.546 | 0.21 | 0.63 |  |
| P54108\|CRIS3_HUMAN | -0.466 | 0.21 | 0.63 |  |
| P20160\|CAP7_HUMAN | -1.290 | 0.21 | 0.63 |  |
| P08246\|ELNE_HUMAN | 0.545 | 0.21 | 0.63 |  |
| O95274\|LYPD3_HUMAN | -0.583 | 0.21 | 0.63 |  |
| P01040\|CYTA_HUMAN | -1.183 | 0.21 | 0.63 |  |
| P52565\|GDIR1_HUMAN | -0.476 | 0.21 | 0.63 |  |
| P11142\|HSP7C_HUMAN | -0.456 | 0.21 | 0.63 |  |
| P68431\|H31_HUMAN | 0.840 | 0.22 | 0.63 |  |
| Q9H2G2\|SLK_HUMAN | 0.388 | 0.22 | 0.64 |  |
| Q13838\|DX39B_HUMAN | -0.832 | 0.22 | 0.64 |  |
| P61158\|ARP3_HUMAN | -0.454 | 0.23 | 0.65 |  |
| Q96FQ6\|S10AG_HUMAN | -0.388 | 0.24 | 0.65 |  |
| P48637\|GSHB_HUMAN | 0.691 | 0.24 | 0.65 |  |
| Q06323\|PSME1_HUMAN | -1.103 | 0.24 | 0.65 |  |
| P17931\|LEG3_HUMAN | -1.023 | 0.24 | 0.66 |  |
| Q92817\|EVPL_HUMAN | 0.914 | 0.25 | 0.67 |  |
| P08311\|CATG_HUMAN | -0.405 | 0.25 | 0.68 |  |
| P10599\|THIO_HUMAN | -0.420 | 0.26 | 0.68 |  |
| P60709\|ACTB_HUMAN | 0.680 | 0.26 | 0.69 |  |
| P52566\|GDIR2_HUMAN | -0.503 | 0.26 | 0.69 |  |
| P25788\|PSA3_HUMAN | -0.359 | 0.27 | 0.70 |  |
| Q8N1N4\|K2C78_HUMAN | -0.401 | 0.27 | 0.70 |  |
| P01591\|IGJ_HUMAN | 0.488 | 0.27 | 0.70 |  |
| P22894\|MMP8_HUMAN | -0.413 | 0.28 | 0.70 |  |
| P61026\|RAB10_HUMAN | -0.445 | 0.28 | 0.70 |  |
| P06731\|CEAM5_HUMAN | -0.324 | 0.28 | 0.70 |  |
| P12429\|ANXA3_HUMAN | 0.579 | 0.28 | 0.70 |  |
| P52209\|6PGD_HUMAN | -0.597 | 0.29 | 0.70 |  |
| P05090\|APOD_HUMAN | 0.368 | 0.30 | 0.71 |  |
| P13688\|CEAM1_HUMAN | -0.327 | 0.30 | 0.71 |  |
| P00734\|THRB_HUMAN | 0.749 | 0.30 | 0.71 |  |
| P35527\|K1C9_HUMAN | 0.813 | 0.30 | 0.71 |  |
| Q02878\|RL6_HUMAN | 0.489 | 0.31 | 0.71 |  |
| P02730\|B3AT_HUMAN | 0.599 | 0.31 | 0.71 |  |
| Q562R1\|ACTBL_HUMAN | -0.558 | 0.31 | 0.71 |  |
| P81605\|DCD_HUMAN | 0.863 | 0.32 | 0.71 |  |
| P12273\|PIP_HUMAN | 0.646 | 0.32 | 0.71 |  |
| P14625\|ENPL_HUMAN | 0.670 | 0.32 | 0.71 |  |
| P02538\|K2C6A_HUMAN | -0.535 | 0.32 | 0.71 |  |
| O43240\|KLK10_HUMAN | -0.425 | 0.32 | 0.71 |  |
| Q15084\|PDIA6_HUMAN | 0.442 | 0.32 | 0.71 |  |
| P52272\|HNRPM_HUMAN | -0.412 | 0.32 | 0.71 |  |
| P61626\|LYSC_HUMAN | 0.354 | 0.32 | 0.71 |  |
| P48594\|SPB4_HUMAN | -0.478 | 0.33 | 0.72 |  |
| Q9NQC3\|RTN4_HUMAN | 0.320 | 0.33 | 0.72 |  |
| P15924\|DESP_HUMAN | 0.703 | 0.34 | 0.72 |  |
| P61160\|ARP2_HUMAN | 0.279 | 0.34 | 0.72 |  |
| P07339\|CATD_HUMAN | 0.403 | 0.35 | 0.72 |  |
| P55072\|TERA_HUMAN | -0.273 | 0.35 | 0.72 |  |
| P31949\|S10AB_HUMAN | 0.318 | 0.35 | 0.72 |  |
| P01598\|KV106_HUMAN | -0.373 | 0.35 | 0.72 |  |
| P08603\|CFAH_HUMAN | -0.396 | 0.35 | 0.72 |  |
| P68104\|EF1A1_HUMAN | -0.854 | 0.35 | 0.73 |  |
| P24158\|PRTN3_HUMAN | 0.343 | 0.36 | 0.73 |  |
| Q9NQ38\|ISK5_HUMAN | -0.346 | 0.37 | 0.73 |  |
| P28676\|GRAN_HUMAN | 0.349 | 0.37 | 0.73 |  |
| P50995\|ANX11_HUMAN | -0.337 | 0.37 | 0.73 |  |
| P11279\|LAMP1_HUMAN | 0.261 | 0.38 | 0.73 |  |
| P62826\|RAN_HUMAN | 0.329 | 0.38 | 0.73 |  |
| Q8TE68\|ES8L1_HUMAN | -0.389 | 0.39 | 0.73 |  |
| O75223\|GGCT_HUMAN | 0.541 | 0.39 | 0.73 |  |
| P61604\|CH10_HUMAN | 0.356 | 0.39 | 0.73 |  |
| P04908\|H2A1B_HUMAN | -0.360 | 0.39 | 0.73 |  |
| Q9Y285\|SYFA_HUMAN | 0.362 | 0.39 | 0.73 |  |
| P62805\|H4_HUMAN | 0.400 | 0.39 | 0.73 |  |
| O15511\|ARPC5_HUMAN | 0.299 | 0.40 | 0.73 |  |
| Q01518\|CAP1_HUMAN | -0.444 | 0.40 | 0.73 |  |
| P04080\|CYTB_HUMAN | 0.422 | 0.40 | 0.73 |  |
| Q14CN2\|CLCA4_HUMAN | -0.347 | 0.40 | 0.73 |  |
| P03973\|SLPI_HUMAN | -0.368 | 0.40 | 0.73 |  |
| P60900\|PSA6_HUMAN | -0.229 | 0.40 | 0.73 |  |
| P22528\|SPR1B_HUMAN | -0.398 | 0.40 | 0.73 |  |
| P06733\|ENOA_HUMAN | -0.248 | 0.41 | 0.73 |  |
| P46776\|RL27A_HUMAN | 0.566 | 0.41 | 0.73 |  |
| P02763\|A1AG1_HUMAN | 0.294 | 0.41 | 0.73 |  |
| Q6UWP8\|SBSN_HUMAN | 0.866 | 0.43 | 0.76 |  |
| P00390\|GSHR_HUMAN | 0.286 | 0.43 | 0.76 |  |
| P62258\|1433E_HUMAN | -0.366 | 0.43 | 0.76 |  |
| P28072\|PSB6_HUMAN | -0.258 | 0.43 | 0.76 |  |
| P01031\|CO5_HUMAN | -0.585 | 0.44 | 0.76 |  |
| P62937\|PPIA_HUMAN | 0.238 | 0.44 | 0.76 |  |
| P06702\|S10A9_HUMAN | -0.307 | 0.44 | 0.76 |  |
| P04264\|K2C1_HUMAN | -0.855 | 0.45 | 0.77 |  |
| P02750\|A2GL_HUMAN | 0.264 | 0.45 | 0.77 |  |
| Q9Y446\|PKP3_HUMAN | 0.376 | 0.46 | 0.78 |  |
| Q04695\|K1C17_HUMAN | -0.388 | 0.47 | 0.78 |  |
| P62701\|RS4X_HUMAN | 0.398 | 0.47 | 0.78 |  |
| P62269\|RS18_HUMAN | 0.196 | 0.47 | 0.78 |  |
| O43175\|SERA_HUMAN | -0.318 | 0.47 | 0.78 |  |
| P04003\|C4BPA_HUMAN | -0.472 | 0.48 | 0.78 |  |
| P62318\|SMD3_HUMAN | 0.236 | 0.48 | 0.78 |  |
| P04075\|ALDOA_HUMAN | 0.278 | 0.48 | 0.78 |  |
| P31146\|COR1A_HUMAN | 0.264 | 0.48 | 0.78 |  |
| Q08188\|TGM3_HUMAN | -0.552 | 0.49 | 0.79 |  |
| P08107\|HSP71_HUMAN | -0.228 | 0.49 | 0.79 |  |
| P31947\|1433S_HUMAN | -0.303 | 0.50 | 0.80 |  |
| P37802\|TAGL2_HUMAN | 0.223 | 0.50 | 0.80 |  |
| P04196\|HRG_HUMAN | 0.223 | 0.51 | 0.81 |  |
| P06576\|ATPB_HUMAN | 0.270 | 0.52 | 0.82 |  |
| P30086\|PEBP1_HUMAN | 0.283 | 0.52 | 0.82 |  |
| P80723\|BASP1_HUMAN | 0.658 | 0.52 | 0.82 |  |
| P62753\|RS6_HUMAN | 0.802 | 0.53 | 0.83 |  |
| O43707\|ACTN4_HUMAN | 0.201 | 0.54 | 0.83 |  |
| P29401\|TKT_HUMAN | 0.255 | 0.54 | 0.83 |  |
| P34932\|HSP74_HUMAN | -0.539 | 0.54 | 0.83 |  |
| P09525\|ANXA4_HUMAN | 0.225 | 0.55 | 0.83 |  |
| Q7Z4W1\|DCXR_HUMAN | 0.268 | 0.55 | 0.83 |  |
| P04632\|CPNS1_HUMAN | 0.146 | 0.55 | 0.83 |  |
| Q00610\|CLH1_HUMAN | 0.245 | 0.56 | 0.83 |  |
| P00338\|LDHA_HUMAN | 0.162 | 0.56 | 0.84 |  |
| P20700\|LMNB1_HUMAN | -0.201 | 0.57 | 0.84 |  |
| P05107\|ITB2_HUMAN | -0.382 | 0.57 | 0.84 |  |
| Q12802\|AKP13_HUMAN | -0.153 | 0.58 | 0.84 |  |
| P13647\|K2C5_HUMAN | 0.250 | 0.58 | 0.84 |  |
| Q9UJ70\|NAGK_HUMAN | 0.131 | 0.58 | 0.84 |  |
| Q13510\|ASAH1_HUMAN | -0.241 | 0.58 | 0.84 |  |
| P13489\|RINI_HUMAN | -0.395 | 0.59 | 0.85 |  |
| P29373\|RABP2_HUMAN | -0.404 | 0.59 | 0.85 |  |
| Q8NFJ5\|RAI3_HUMAN | -0.191 | 0.60 | 0.85 |  |
| P04792\|HSPB1_HUMAN | -0.292 | 0.61 | 0.86 |  |
| P47756\|CAPZB_HUMAN | -0.202 | 0.61 | 0.86 |  |
| P59665\|DEF1_HUMAN | 0.268 | 0.61 | 0.86 |  |
| Q7Z353\|HDX_HUMAN | -0.226 | 0.62 | 0.86 |  |
| P13639\|EF2_HUMAN | -0.225 | 0.62 | 0.86 |  |
| P20810\|ICAL_HUMAN | -0.232 | 0.62 | 0.86 |  |
| O14950\|ML12B_HUMAN | 0.188 | 0.62 | 0.86 |  |
| P13987\|CD59_HUMAN | -0.286 | 0.63 | 0.86 |  |
| O60814\|H2B1K_HUMAN | -0.175 | 0.63 | 0.86 |  |
| P30050\|RL12_HUMAN | -0.119 | 0.63 | 0.86 |  |
| P20930\|FILA_HUMAN | -0.262 | 0.63 | 0.86 |  |
| P07305\|H10_HUMAN | 0.182 | 0.63 | 0.86 |  |
| P41218\|MNDA_HUMAN | 0.217 | 0.64 | 0.86 |  |
| O60888\|CUTA_HUMAN | 0.176 | 0.64 | 0.86 |  |
| P35579\|MYH9_HUMAN | 0.280 | 0.64 | 0.86 |  |
| P08670\|VIME_HUMAN | 0.197 | 0.64 | 0.86 |  |
| P18054\|LOX12_HUMAN | 0.223 | 0.65 | 0.86 |  |
| P06454\|PTMA_HUMAN | 0.272 | 0.66 | 0.86 |  |
| P40121\|CAPG_HUMAN | 0.164 | 0.66 | 0.86 |  |
| P01024\|CO3_HUMAN | 0.230 | 0.66 | 0.86 |  |
| O60218\|AK1BA_HUMAN | 0.182 | 0.66 | 0.86 |  |
| P01621\|KV303_HUMAN | -0.119 | 0.66 | 0.86 |  |
| P10606\|COX5B_HUMAN | -0.218 | 0.67 | 0.86 |  |
| P22314\|UBA1_HUMAN | -0.164 | 0.67 | 0.86 |  |
| P04040\|CATA_HUMAN | 0.171 | 0.67 | 0.86 |  |
| P00747\|PLMN_HUMAN | 0.242 | 0.67 | 0.86 |  |
| P02533\|K1C14_HUMAN | 0.210 | 0.68 | 0.86 |  |
| Q9BVC6\|TM109_HUMAN | 0.169 | 0.69 | 0.86 |  |
| P26583\|HMGB2_HUMAN | 0.201 | 0.69 | 0.86 |  |
| P23284\|PPIB_HUMAN | 0.164 | 0.69 | 0.86 |  |
| Q9BRF8\|CPPED_HUMAN | 0.106 | 0.69 | 0.86 |  |
| P00966\|ASSY_HUMAN | 0.200 | 0.69 | 0.86 |  |
| Q7Z794\|K2C1B_HUMAN | -0.239 | 0.71 | 0.88 |  |
| Q9HDC9\|APMAP_HUMAN | 0.133 | 0.71 | 0.88 |  |
| P31946\|1433B_HUMAN | 0.203 | 0.72 | 0.88 |  |
| Q92876\|KLK6_HUMAN | 0.306 | 0.72 | 0.88 |  |
| P13473\|LAMP2_HUMAN | -0.089 | 0.72 | 0.88 |  |
| P05120\|PAI2_HUMAN | 0.119 | 0.73 | 0.88 |  |
| P98160\|PGBM_HUMAN | 0.268 | 0.73 | 0.88 |  |
| P14780\|MMP9_HUMAN | 0.119 | 0.74 | 0.89 |  |
| P05387\|RLA2_HUMAN | 0.224 | 0.74 | 0.89 |  |
| P12035\|K2C3_HUMAN | 0.140 | 0.75 | 0.90 |  |
| Q6P4A8\|PLBL1_HUMAN | -0.112 | 0.75 | 0.90 |  |
| P15104\|GLNA_HUMAN | 0.136 | 0.76 | 0.90 |  |
| P30049\|ATPD_HUMAN | 0.111 | 0.76 | 0.90 |  |
| P04179\|SODM_HUMAN | 0.173 | 0.76 | 0.90 |  |
| P07108\|ACBP_HUMAN | 0.251 | 0.77 | 0.90 |  |
| Q9HD89\|RETN_HUMAN | 0.090 | 0.77 | 0.90 |  |
| Q9Y6R7\|FCGBP_HUMAN | 0.090 | 0.77 | 0.90 |  |
| O75874\|IDHC_HUMAN | 0.210 | 0.78 | 0.91 |  |
| P01765\|HV304_HUMAN | 0.094 | 0.80 | 0.92 |  |
| Q9NZD2\|GLTP_HUMAN | -0.108 | 0.80 | 0.92 |  |
| P08758\|ANXA5_HUMAN | 0.149 | 0.80 | 0.92 |  |
| Q8WXK1\|ASB15_HUMAN | 0.107 | 0.81 | 0.92 |  |
| P0C0S8\|H2A1_HUMAN | 0.085 | 0.81 | 0.92 |  |
| P00491\|PNPH_HUMAN | -0.096 | 0.81 | 0.92 |  |
| Q9H299\|SH3L3_HUMAN | -0.112 | 0.82 | 0.92 |  |
| P00915\|CAH1_HUMAN | 0.180 | 0.83 | 0.93 |  |
| P08779\|K1C16_HUMAN | -0.097 | 0.83 | 0.93 |  |
| P01042\|KNG1_HUMAN | 0.156 | 0.83 | 0.93 |  |
| P60174\|TPIS_HUMAN | -0.067 | 0.84 | 0.93 |  |
| P01770\|HV309_HUMAN | 0.068 | 0.84 | 0.93 |  |
| Q13835\|PKP1_HUMAN | 0.065 | 0.84 | 0.93 |  |
| Q08380\|LG3BP_HUMAN | 0.074 | 0.86 | 0.95 |  |
| Q99102\|MUC4_HUMAN | -0.101 | 0.86 | 0.95 |  |
| P18206\|VINC_HUMAN | -0.047 | 0.87 | 0.96 |  |
| P30041\|PRDX6_HUMAN | 0.056 | 0.88 | 0.96 |  |
| P07355\|ANXA2_HUMAN | 0.063 | 0.88 | 0.96 |  |
| P13645\|K1C10_HUMAN | 0.070 | 0.89 | 0.96 |  |
| Q14002\|CEAM7_HUMAN | 0.080 | 0.89 | 0.96 |  |
| P17213\|BPI_HUMAN | -0.089 | 0.89 | 0.96 |  |
| O00391\|QSOX1_HUMAN | -0.099 | 0.89 | 0.96 |  |
| Q6N043\|Z280D_HUMAN | 0.024 | 0.92 | 0.98 |  |
| P29692\|EF1D_HUMAN | -0.029 | 0.93 | 0.98 |  |
| P06396\|GELS_HUMAN | -0.028 | 0.93 | 0.98 |  |
| P30048\|PRDX3_HUMAN | 0.025 | 0.93 | 0.98 |  |
| P01011\|AACT_HUMAN | 0.033 | 0.94 | 0.98 |  |
| P07858\|CATB_HUMAN | -0.042 | 0.94 | 0.98 |  |
| O95171\|SCEL_HUMAN | -0.020 | 0.94 | 0.98 |  |
| P07384\|CAN1_HUMAN | 0.056 | 0.94 | 0.98 |  |
| Q6XQN6\|PNCB_HUMAN | -0.031 | 0.94 | 0.98 |  |
| P51149\|RAB7A_HUMAN | 0.024 | 0.95 | 0.98 |  |
| P08727\|K1C19_HUMAN | 0.060 | 0.95 | 0.98 |  |
| O75594\|PGRP1_HUMAN | 0.019 | 0.95 | 0.98 |  |
| P35268\|RL22_HUMAN | 0.029 | 0.95 | 0.98 |  |
| O43760\|SNG2_HUMAN | -0.022 | 0.95 | 0.98 |  |
| P61019\|RAB2A_HUMAN | -0.014 | 0.97 | 0.99 |  |
| P35232\|PHB_HUMAN | -0.015 | 0.97 | 0.99 |  |
| P27797\|CALR_HUMAN | -0.013 | 0.98 | 0.99 |  |
| P30044\|PRDX5_HUMAN | -0.008 | 0.99 | 0.99 |  |
| P19827\|ITIH1_HUMAN | -0.005 | 0.99 | 0.99 |  |
| P46781\|RS9_HUMAN | 0.008 | 0.99 | 0.99 |  |
| P84098\|RL19_HUMAN | 0.001 | 1.00 | 1.00 |  |

**Supplementary Table S3**: List of identified proteins and their log2 fold change values comparing cases and controls in plasma samples with unadjusted and adjusted p values (Benjamini–Hochberg correction method).

| **Uniprot** | **Gene name** | **Log FC** | **P.Value** | **adj.P.Val** |
| --- | --- | --- | --- | --- |
| P02652 | APOA2 | -0.655 | <0.0001 | 0.0029 |
| P02654 | APOC1 | -0.563 | <0.0001 | 0.0055 |
| P00738 | HP | 1.131 | <0.0001 | 0.011 |
| P05090 | APOD | -0.546 | <0.0001 | 0.011 |
| P05452 | CLEC3B | -0.405 | 0.00013 | 0.011 |
| P06576 | ATP5F1B | 0.488 | 0.00014 | 0.011 |
| P22352 | GPX3 | -0.408 | 0.00015 | 0.011 |
| P35858 | IGFALS | -0.458 | 0.00017 | 0.011 |
| P02741 | CRP | 1.129 | 0.00076 | 0.045 |
| P00747 | PLG | -0.302 | 0.00087 | 0.046 |
| P08185 | SERPINA6 | -0.355 | 0.0010 | 0.046 |
| Q99729 | HNRNPAB | 0.698 | 0.0011 | 0.046 |
| P28482 | MAPK1 | 1.596 | 0.0012 | 0.046 |
| P01042 | KNG1 | -0.273 | 0.0013 | 0.046 |
| Q96KN2 | CNDP1 | -0.520 | 0.0013 | 0.046 |
| P03952 | KLKB1 | -0.307 | 0.0015 | 0.049 |
| P14151 | SELL | -0.317 | 0.0017 | 0.053 |
| P05165 | PCCA | 0.944 | 0.0020 | 0.054 |
| Q9UHG3 | PCYOX1 | -0.736 | 0.0020 | 0.054 |
| P04196 | HRG | -0.377 | 0.0020 | 0.054 |
| O95445 | APOM | -0.379 | 0.0030 | 0.077 |
| P30041 | PRDX6 | 0.299 | 0.0032 | 0.079 |
| O14818 | PSMA7 | 0.906 | 0.0037 | 0.086 |
| Q96PD5 | PGLYRP2 | -0.275 | 0.0047 | 0.098 |
| O75882 | ATRN | -0.271 | 0.0047 | 0.098 |
| P00742 | F10 | -0.201 | 0.0048 | 0.098 |
| Q9NTX5 | ECHDC1 | -0.399 | 0.0053 | 0.11 |
| P02753 | RBP4 | -0.325 | 0.0066 | 0.11 |
| P00338 | LDHA | 0.367 | 0.0067 | 0.11 |
| P02768 | ALB | -0.948 | 0.0067 | 0.11 |
| Q99459 | CDC5L | 0.477 | 0.0069 | 0.11 |
| Q99832 | CCT7 | 0.413 | 0.0069 | 0.11 |
| P02656 | APOC3 | -0.503 | 0.0073 | 0.12 |
| O43707 | ACTN4 | 0.319 | 0.0079 | 0.12 |
| P27824 | CANX | 0.321 | 0.0082 | 0.12 |
| A0PJW6 | TMEM223 | 0.309 | 0.0083 | 0.12 |
| P10645 | CHGA | -0.463 | 0.0093 | 0.13 |
| P06396 | GSN | -0.274 | 0.0097 | 0.13 |
| P29622 | SERPINA4 | -0.274 | 0.0099 | 0.13 |
| P46939 | UTRN | -0.369 | 0.0100 | 0.13 |
| Q96BZ4 | PLD4 | 0.451 | 0.011 | 0.14 |
| P02766 | TTR | -0.323 | 0.012 | 0.14 |
| P00441 | SOD1 | -0.350 | 0.012 | 0.14 |
| Q12931 | TRAP1 | -0.568 | 0.012 | 0.14 |
| P02750 | LRG1 | 0.296 | 0.013 | 0.15 |
| P27169 | PON1 | -0.290 | 0.013 | 0.16 |
| P06276 | BCHE | -0.264 | 0.014 | 0.16 |
| P43251 | BTD | -0.239 | 0.015 | 0.16 |
| Q9Y6U3 | SCIN | -0.325 | 0.015 | 0.17 |
| P01344 | IGF2 | -0.228 | 0.016 | 0.17 |
| Q13790 | APOF | -0.256 | 0.016 | 0.17 |
| P46821 | MAP1B | 0.666 | 0.017 | 0.18 |
| P05166 | PCCB | -0.739 | 0.018 | 0.18 |
| Q13103 | SPP2 | 0.528 | 0.018 | 0.18 |
| P01009 | SERPINA1 | 0.235 | 0.024 | 0.23 |
| P23229 | ITGA6 | -0.441 | 0.024 | 0.23 |
| P02748 | C9 | 0.250 | 0.025 | 0.23 |
| P12081 | HARS1 | -0.245 | 0.025 | 0.23 |
| P68871 | HBB | 0.616 | 0.027 | 0.24 |
| P35542 | SAA4 | 0.327 | 0.029 | 0.25 |
| P02647 | APOA1 | -0.336 | 0.029 | 0.25 |
| Q7KZF4 | SND1 | -1.874 | 0.029 | 0.25 |
| P28066 | PSMA5 | -0.390 | 0.032 | 0.27 |
| Q6UXB8 | PI16 | -0.442 | 0.033 | 0.27 |
| Q96BY6 | DOCK10 | 0.364 | 0.033 | 0.27 |
| Q9H3U1 | UNC45A | 0.404 | 0.034 | 0.27 |
| P25705 | ATP5F1A | -0.811 | 0.037 | 0.29 |
| Q86VP6 | CAND1 | 0.529 | 0.038 | 0.29 |
| P06702 | S100A9 | 0.506 | 0.038 | 0.29 |
| P01861 | IGHG4 | 1.203 | 0.038 | 0.29 |
| P01011 | SERPINA3 | 0.162 | 0.039 | 0.29 |
| O75376 | NCOR1 | 0.819 | 0.041 | 0.30 |
| P16070 | CD44 | -0.212 | 0.043 | 0.31 |
| P25787 | PSMA2 | -0.411 | 0.044 | 0.31 |
| P01857 | IGHG1 | -0.738 | 0.044 | 0.31 |
| Q04756 | HGFAC | -0.449 | 0.045 | 0.31 |
| P61224 | RAP1B | -0.338 | 0.045 | 0.31 |
| P09651 | HNRNPA1 | -0.352 | 0.050 | 0.34 |
| P02774 | GC | -0.154 | 0.050 | 0.34 |
| Q5SSJ5 | HP1BP3 | 0.150 | 0.051 | 0.34 |
| Q08257 | CRYZ | -0.305 | 0.055 | 0.36 |
| Q8NBS9 | TXNDC5 | -0.411 | 0.055 | 0.36 |
| P23368 | ME2 | 0.302 | 0.056 | 0.36 |
| Q01581 | HMGCS1 | -0.176 | 0.057 | 0.36 |
| Q5JRA6 | MIA3 | 0.454 | 0.057 | 0.36 |
| P02792 | FTL | 0.225 | 0.063 | 0.39 |
| Q9Y6C2 | EMILIN1 | 0.760 | 0.064 | 0.39 |
| P22102 | GART | 0.405 | 0.064 | 0.39 |
| P22792 | CPN2 | -0.188 | 0.065 | 0.39 |
| P54108 | CRISP3 | -0.430 | 0.069 | 0.41 |
| P02655 | APOC2 | -0.290 | 0.070 | 0.41 |
| P17936 | IGFBP3 | -0.244 | 0.071 | 0.41 |
| P04114 | APOB | -0.223 | 0.071 | 0.41 |
| Q14146 | URB2 | -0.246 | 0.072 | 0.41 |
| P62847 | RPS24 | -0.308 | 0.075 | 0.42 |
| P05154 | SERPINA5 | -0.264 | 0.075 | 0.42 |
| P01834 | IGKC | -0.637 | 0.077 | 0.42 |
| Q16851 | UGP2 | -0.929 | 0.078 | 0.42 |
| P10909 | CLU | -0.208 | 0.079 | 0.42 |
| P19823 | ITIH2 | -0.130 | 0.081 | 0.43 |
| P00739 | HPR | -0.308 | 0.081 | 0.43 |
| P50225 | SULT1A1 | 1.209 | 0.085 | 0.43 |
| P07237 | P4HB | -0.199 | 0.085 | 0.43 |
| P18428 | LBP | 0.333 | 0.085 | 0.43 |
| Q13557 | CAMK2D | -0.961 | 0.087 | 0.43 |
| P55058 | PLTP | -0.251 | 0.087 | 0.43 |
| P04070 | PROC | -0.168 | 0.087 | 0.43 |
| P06737 | PYGL | -0.233 | 0.088 | 0.43 |
| O75822 | EIF3J | 0.814 | 0.088 | 0.43 |
| Q13740 | ALCAM | -0.655 | 0.089 | 0.43 |
| P00734 | F2 | -0.145 | 0.090 | 0.43 |
| P02787 | TF | -0.428 | 0.092 | 0.44 |
| P06753 | TPM3 | 0.593 | 0.094 | 0.44 |
| P42330 | AKR1C3 | 0.313 | 0.098 | 0.45 |
| P80108 | GPLD1 | -0.169 | 0.099 | 0.45 |
| P69905 | HBA1 | 0.451 | 0.099 | 0.45 |
| P02765 | AHSG | -0.190 | 0.10 | 0.46 |
| Q9NTJ3 | SMC4 | -0.982 | 0.10 | 0.46 |
| P04406 | GAPDH | -0.211 | 0.10 | 0.46 |
| P02675 | FGB | 0.277 | 0.11 | 0.48 |
| Q6UWP8 | SBSN | -0.389 | 0.11 | 0.48 |
| P07357 | C8A | -0.160 | 0.11 | 0.48 |
| P02743 | APCS | -0.224 | 0.11 | 0.48 |
| Q13228 | SELENBP1 | 0.371 | 0.11 | 0.48 |
| Q86UX7 | FERMT3 | -0.091 | 0.11 | 0.48 |
| P51570 | GALK1 | 0.349 | 0.11 | 0.48 |
| Q02880 | TOP2B | -0.175 | 0.11 | 0.48 |
| Q96IY4 | CPB2 | -0.121 | 0.12 | 0.50 |
| O43776 | NARS1 | 0.189 | 0.12 | 0.51 |
| O00533 | CHL1 | -0.165 | 0.13 | 0.52 |
| P02452 | COL1A1 | 0.513 | 0.13 | 0.53 |
| P08195 | SLC3A2 | -0.304 | 0.13 | 0.53 |
| P09619 | PDGFRB | 0.327 | 0.13 | 0.53 |
| P08697 | SERPINF2 | -0.136 | 0.13 | 0.53 |
| P08514 | ITGA2B | -0.925 | 0.14 | 0.53 |
| P04004 | VTN | -0.150 | 0.14 | 0.54 |
| P98160 | HSPG2 | 0.537 | 0.14 | 0.54 |
| Q13263 | TRIM28 | 0.492 | 0.14 | 0.54 |
| P04264 | KRT1 | -0.586 | 0.14 | 0.54 |
| Q99460 | PSMD1 | 0.386 | 0.14 | 0.54 |
| P23142 | FBLN1 | -0.160 | 0.14 | 0.54 |
| Q9Y2S2 | CRYL1 | -0.181 | 0.14 | 0.54 |
| O75636 | FCN3 | -0.154 | 0.15 | 0.54 |
| O75116 | ROCK2 | 0.278 | 0.15 | 0.54 |
| P07355 | ANXA2 | 0.891 | 0.15 | 0.54 |
| P26038 | MSN | 0.160 | 0.15 | 0.54 |
| P07108 | DBI | 0.798 | 0.15 | 0.54 |
| O75369 | FLNB | 0.376 | 0.15 | 0.54 |
| P41240 | CSK | -0.285 | 0.15 | 0.54 |
| P02749 | APOH | -0.142 | 0.15 | 0.54 |
| P04211 | IGLV7-43 | 0.466 | 0.15 | 0.54 |
| P02679 | FGG | 0.238 | 0.16 | 0.54 |
| P00450 | CP | -0.196 | 0.16 | 0.54 |
| P49247 | RPIA | -0.628 | 0.16 | 0.54 |
| P01019 | AGT | -0.187 | 0.16 | 0.55 |
| P12259 | F5 | -0.236 | 0.16 | 0.55 |
| P01859 | IGHG2 | -0.957 | 0.16 | 0.55 |
| O15145 | ARPC3 | 0.218 | 0.17 | 0.56 |
| O60610 | DIAPH1 | 0.462 | 0.17 | 0.56 |
| P20700 | LMNB1 | 0.331 | 0.17 | 0.56 |
| P35900 | KRT20 | 0.374 | 0.17 | 0.56 |
| O75347 | TBCA | -0.343 | 0.17 | 0.56 |
| Q15485 | FCN2 | -0.320 | 0.17 | 0.56 |
| Q9ULV4 | CORO1C | -0.230 | 0.18 | 0.57 |
| P23528 | CFL1 | -0.348 | 0.18 | 0.58 |
| Q9UGM5 | FETUB | -0.139 | 0.18 | 0.58 |
| Q99784 | OLFM1 | 0.193 | 0.18 | 0.58 |
| Q13201 | MMRN1 | 0.333 | 0.18 | 0.58 |
| Q3LXA3 | TKFC | 0.558 | 0.19 | 0.58 |
| P07900 | HSP90AA1 | -0.551 | 0.19 | 0.58 |
| P19827 | ITIH1 | -0.094 | 0.19 | 0.58 |
| P08294 | SOD3 | 0.671 | 0.19 | 0.58 |
| P49368 | CCT3 | 0.439 | 0.19 | 0.58 |
| Q92626 | PXDN | 0.200 | 0.19 | 0.58 |
| O14791 | APOL1 | -0.138 | 0.19 | 0.58 |
| Q15848 | ADIPOQ | -0.222 | 0.19 | 0.58 |
| P38646 | HSPA9 | -0.288 | 0.19 | 0.58 |
| P60174 | TPI1 | -0.240 | 0.20 | 0.58 |
| P12277 | CKB | -0.959 | 0.20 | 0.58 |
| O60664 | PLIN3 | 0.313 | 0.20 | 0.59 |
| P11279 | LAMP1 | -0.225 | 0.20 | 0.59 |
| P14625 | HSP90B1 | -0.322 | 0.20 | 0.60 |
| Q9HDC9 | APMAP | -0.752 | 0.21 | 0.60 |
| P13473 | LAMP2 | -0.251 | 0.21 | 0.61 |
| P51884 | LUM | -0.156 | 0.21 | 0.61 |
| Q7Z3U7 | MON2 | 0.207 | 0.22 | 0.61 |
| P02760 | AMBP | -0.118 | 0.22 | 0.61 |
| Q562R1 | ACTBL2 | 0.209 | 0.22 | 0.61 |
| P55786 | NPEPPS | -0.786 | 0.22 | 0.61 |
| P05156 | CFI | 0.114 | 0.22 | 0.62 |
| Q9P260 | RELCH | -0.253 | 0.22 | 0.62 |
| P01024 | C3 | 0.123 | 0.22 | 0.62 |
| Q16853 | AOC3 | -0.636 | 0.22 | 0.62 |
| P20810 | CAST | 0.400 | 0.23 | 0.62 |
| P12429 | ANXA3 | -0.342 | 0.23 | 0.62 |
| Q15691 | MAPRE1 | 0.185 | 0.23 | 0.62 |
| P07359 | GP1BA | -0.124 | 0.23 | 0.63 |
| Q8N163 | CCAR2 | 0.210 | 0.23 | 0.63 |
| P05160 | F13B | -0.219 | 0.24 | 0.63 |
| P00736 | C1R | 0.083 | 0.24 | 0.63 |
| B9A064 | IGLL5 | 0.573 | 0.24 | 0.63 |
| Q14141 | SEPTIN6 | -0.384 | 0.24 | 0.63 |
| Q14974 | KPNB1 | -0.166 | 0.24 | 0.64 |
| P00488 | F13A1 | -0.175 | 0.25 | 0.64 |
| P01871 | IGHM | -0.315 | 0.25 | 0.64 |
| P29350 | PTPN6 | -0.151 | 0.25 | 0.65 |
| P48740 | MASP1 | -0.217 | 0.26 | 0.65 |
| Q9UI17 | DMGDH | 0.351 | 0.26 | 0.65 |
| O43505 | B4GAT1 | -0.654 | 0.26 | 0.65 |
| P01008 | SERPINC1 | -0.096 | 0.26 | 0.65 |
| Q9UNW1 | MINPP1 | 0.268 | 0.27 | 0.68 |
| P52566 | ARHGDIB | -0.231 | 0.27 | 0.68 |
| Q9UKX7 | NUP50 | 0.439 | 0.27 | 0.68 |
| Q6PI48 | DARS2 | -0.343 | 0.27 | 0.68 |
| Q14520 | HABP2 | -0.128 | 0.28 | 0.68 |
| Q06033 | ITIH3 | 1.400 | 0.28 | 0.68 |
| Q86WR0 | CCDC25 | -0.098 | 0.28 | 0.68 |
| P02763 | ORM1 | 0.237 | 0.28 | 0.68 |
| P07195 | LDHB | 0.372 | 0.28 | 0.68 |
| P31939 | ATIC | 0.552 | 0.28 | 0.68 |
| Q15029 | EFTUD2 | 0.213 | 0.28 | 0.68 |
| P02649 | APOE | -0.112 | 0.29 | 0.68 |
| P05109 | S100A8 | 0.539 | 0.29 | 0.68 |
| P01023 | A2M | 0.225 | 0.29 | 0.68 |
| Q9Y490 | TLN1 | -0.249 | 0.29 | 0.68 |
| Q9UMX5 | NENF | 0.169 | 0.29 | 0.68 |
| O75122 | CLASP2 | -0.242 | 0.29 | 0.68 |
| P08519 | LPA | 0.493 | 0.29 | 0.68 |
| O76013 | KRT36 | -0.228 | 0.29 | 0.68 |
| P13667 | PDIA4 | 0.286 | 0.30 | 0.69 |
| Q15149 | PLEC | 0.282 | 0.30 | 0.69 |
| P07360 | C8G | 0.459 | 0.30 | 0.69 |
| O00203 | AP3B1 | 0.189 | 0.30 | 0.69 |
| P00966 | ASS1 | -0.160 | 0.30 | 0.69 |
| O43175 | PHGDH | -0.139 | 0.30 | 0.69 |
| P07954 | FH | -0.176 | 0.31 | 0.69 |
| Q15075 | EEA1 | -0.732 | 0.31 | 0.69 |
| O14556 | GAPDHS | 0.254 | 0.31 | 0.69 |
| P07942 | LAMB1 | 0.200 | 0.31 | 0.69 |
| Q96KP4 | CNDP2 | 0.184 | 0.31 | 0.69 |
| P10586 | PTPRF | 0.457 | 0.31 | 0.69 |
| P07225 | PROS1 | -0.086 | 0.31 | 0.69 |
| P02671 | FGA | 0.165 | 0.32 | 0.70 |
| P37802 | TAGLN2 | 0.078 | 0.32 | 0.70 |
| P08670 | VIM | -0.155 | 0.32 | 0.70 |
| Q14315 | FLNC | -0.174 | 0.32 | 0.70 |
| Q01082 | SPTBN1 | 0.233 | 0.33 | 0.70 |
| P02775 | PPBP | 0.230 | 0.33 | 0.70 |
| P20930 | FLG | -0.278 | 0.33 | 0.70 |
| P23396 | RPS3 | -0.258 | 0.33 | 0.70 |
| P01876 | IGHA1 | -0.357 | 0.33 | 0.70 |
| Q14112 | NID2 | -0.308 | 0.34 | 0.71 |
| Q9H0W9 | C11orf54 | -0.438 | 0.34 | 0.71 |
| Q9Y3B4 | SF3B6 | 0.186 | 0.34 | 0.71 |
| P15169 | CPN1 | -0.071 | 0.34 | 0.71 |
| Q9Y5Y7 | LYVE1 | -0.119 | 0.34 | 0.71 |
| P33176 | KIF5B | -0.130 | 0.34 | 0.71 |
| P35611 | ADD1 | 0.285 | 0.34 | 0.71 |
| P01031 | C5 | 0.076 | 0.35 | 0.71 |
| Q02790 | FKBP4 | -0.176 | 0.35 | 0.71 |
| P49321 | NASP | -0.370 | 0.35 | 0.71 |
| Q02747 | GUCA2A | 0.196 | 0.35 | 0.71 |
| P01714 | IGLV3-19 | 0.464 | 0.35 | 0.71 |
| Q14914 | PTGR1 | 0.174 | 0.35 | 0.71 |
| P68036 | UBE2L3 | -0.149 | 0.36 | 0.71 |
| P48047 | ATP5PO | -0.507 | 0.36 | 0.71 |
| P27918 | CFP | -0.130 | 0.36 | 0.71 |
| Q9P2E9 | RRBP1 | 0.351 | 0.36 | 0.72 |
| Q9BRP8 | PYM1 | -0.138 | 0.36 | 0.72 |
| P84095 | RHOG | 0.306 | 0.37 | 0.72 |
| P43490 | NAMPT | -0.419 | 0.37 | 0.72 |
| P25311 | AZGP1 | -0.106 | 0.37 | 0.72 |
| P19320 | VCAM1 | -0.201 | 0.37 | 0.72 |
| P36955 | SERPINF1 | -0.071 | 0.37 | 0.72 |
| Q9ULA0 | DNPEP | 0.204 | 0.37 | 0.72 |
| P09871 | C1S | -0.063 | 0.37 | 0.72 |
| P34932 | HSPA4 | 0.143 | 0.38 | 0.72 |
| P62263 | RPS14 | -0.385 | 0.38 | 0.72 |
| Q9NPD3 | EXOSC4 | 0.122 | 0.38 | 0.72 |
| O14980 | XPO1 | 0.110 | 0.38 | 0.72 |
| P06733 | ENO1 | -0.356 | 0.38 | 0.72 |
| Q10589 | BST2 | 0.107 | 0.38 | 0.72 |
| P05164 | MPO | 0.097 | 0.38 | 0.72 |
| P30101 | PDIA3 | 0.206 | 0.39 | 0.73 |
| P22087 | FBL | -0.318 | 0.39 | 0.73 |
| Q96JB5 | CDK5RAP3 | -0.202 | 0.39 | 0.73 |
| P50570 | DNM2 | -0.173 | 0.39 | 0.73 |
| P16284 | PECAM1 | -0.296 | 0.41 | 0.74 |
| P02751 | FN1 | 0.280 | 0.41 | 0.74 |
| P00915 | CA1 | -0.224 | 0.41 | 0.74 |
| P11047 | LAMC1 | -0.578 | 0.41 | 0.74 |
| P30084 | ECHS1 | 0.157 | 0.41 | 0.74 |
| P04278 | SHBG | -0.150 | 0.41 | 0.74 |
| Q9Y6R7 | FCGBP | 0.324 | 0.41 | 0.74 |
| Q9Y4L1 | HYOU1 | 0.112 | 0.41 | 0.74 |
| P11413 | G6PD | 0.164 | 0.41 | 0.74 |
| Q9UBG0 | MRC2 | -0.228 | 0.41 | 0.74 |
| P01717 | IGLV3-25 | 0.315 | 0.42 | 0.74 |
| Q15582 | TGFBI | 0.485 | 0.42 | 0.74 |
| P02747 | C1QC | -0.126 | 0.42 | 0.74 |
| Q9NNW7 | TXNRD2 | -0.246 | 0.42 | 0.74 |
| P43652 | AFM | -0.090 | 0.42 | 0.74 |
| P21796 | VDAC1 | -0.180 | 0.42 | 0.74 |
| P22897 | MRC1 | -0.093 | 0.42 | 0.74 |
| P02746 | C1QB | -0.108 | 0.42 | 0.74 |
| P31327 | CPS1 | -0.121 | 0.42 | 0.74 |
| P19652 | ORM2 | 0.151 | 0.43 | 0.74 |
| Q9UHD1 | CHORDC1 | 0.600 | 0.43 | 0.74 |
| P13591 | NCAM1 | -0.119 | 0.43 | 0.74 |
| Q08380 | LGALS3BP | 0.103 | 0.43 | 0.74 |
| P16435 | POR | -0.169 | 0.43 | 0.74 |
| Q9Y446 | PKP3 | -0.081 | 0.44 | 0.75 |
| Q86YZ3 | HRNR | 0.180 | 0.44 | 0.75 |
| Q01518 | CAP1 | 0.554 | 0.45 | 0.76 |
| P36222 | CHI3L1 | 0.108 | 0.45 | 0.76 |
| O00391 | QSOX1 | -0.212 | 0.45 | 0.76 |
| Q9Y2Z0 | SUGT1 | -0.144 | 0.46 | 0.77 |
| P35052 | GPC1 | 0.143 | 0.46 | 0.78 |
| P19367 | HK1 | 0.152 | 0.47 | 0.78 |
| Q12805 | EFEMP1 | 0.077 | 0.47 | 0.78 |
| P07358 | C8B | -0.084 | 0.47 | 0.78 |
| P00533 | EGFR | 0.133 | 0.47 | 0.78 |
| P21333 | FLNA | -0.109 | 0.48 | 0.79 |
| P0C0L5 | C4B | 0.127 | 0.48 | 0.79 |
| O15143 | ARPC1B | -0.059 | 0.48 | 0.79 |
| P08603 | CFH | 0.088 | 0.48 | 0.79 |
| P53396 | ACLY | -0.262 | 0.49 | 0.79 |
| Q14690 | PDCD11 | 0.235 | 0.49 | 0.79 |
| Q9P0L0 | VAPA | 0.257 | 0.49 | 0.79 |
| P30153 | PPP2R1A | -0.256 | 0.49 | 0.79 |
| P38606 | ATP6V1A | 0.151 | 0.49 | 0.79 |
| P12955 | PEPD | 0.154 | 0.50 | 0.79 |
| P02790 | HPX | 0.055 | 0.50 | 0.80 |
| P49588 | AARS1 | 0.053 | 0.51 | 0.80 |
| Q16610 | ECM1 | -0.131 | 0.51 | 0.80 |
| P05186 | ALPL | 0.056 | 0.51 | 0.81 |
| Q8NC51 | SERBP1 | -0.569 | 0.51 | 0.81 |
| O43143 | DHX15 | 0.170 | 0.51 | 0.81 |
| P04217 | A1BG | -0.067 | 0.51 | 0.81 |
| P17948 | FLT1 | 0.052 | 0.52 | 0.81 |
| P09104 | ENO2 | -0.061 | 0.52 | 0.81 |
| Q16181 | SEPTIN7 | 0.448 | 0.52 | 0.81 |
| P24666 | ACP1 | -0.119 | 0.52 | 0.81 |
| Q7Z7E8 | UBE2Q1 | -0.174 | 0.53 | 0.81 |
| P33908 | MAN1A1 | 0.085 | 0.53 | 0.81 |
| Q9UDT6 | CLIP2 | 0.058 | 0.53 | 0.81 |
| P13645 | KRT10 | 0.205 | 0.54 | 0.82 |
| P26599 | PTBP1 | -0.259 | 0.54 | 0.82 |
| P61769 | B2M | 0.088 | 0.54 | 0.82 |
| P14314 | PRKCSH | -0.541 | 0.54 | 0.82 |
| Q13576 | IQGAP2 | 0.093 | 0.54 | 0.82 |
| P02042 | HBD | 0.268 | 0.55 | 0.83 |
| P62701 | RPS4X | 0.051 | 0.55 | 0.83 |
| P31943 | HNRNPH1 | 0.077 | 0.55 | 0.83 |
| Q9HC38 | GLOD4 | -0.111 | 0.56 | 0.83 |
| P80748 | IGLV3-21 | -0.316 | 0.56 | 0.83 |
| P30050 | RPL12 | -0.122 | 0.56 | 0.83 |
| P61604 | HSPE1 | -0.154 | 0.56 | 0.83 |
| O75874 | IDH1 | -0.107 | 0.56 | 0.83 |
| P13647 | KRT5 | 0.070 | 0.56 | 0.83 |
| P01771 | IGHV3-33 | 0.466 | 0.57 | 0.83 |
| P22891 | PROZ | -0.084 | 0.57 | 0.84 |
| P19971 | TYMP | -0.229 | 0.57 | 0.84 |
| P04003 | C4BPA | 0.057 | 0.58 | 0.84 |
| P04180 | LCAT | -0.039 | 0.58 | 0.84 |
| Q9UK55 | SERPINA10 | -0.047 | 0.58 | 0.84 |
| P35908 | KRT2 | 0.293 | 0.59 | 0.85 |
| Q6EMK4 | VASN | -0.050 | 0.59 | 0.85 |
| Q00839 | HNRNPU | 0.062 | 0.59 | 0.85 |
| P08107 | HSPA1A | 0.061 | 0.59 | 0.85 |
| P24821 | TNC | -0.069 | 0.59 | 0.85 |
| P49908 | SELENOP | -0.130 | 0.59 | 0.85 |
| P81605 | DCD | -0.180 | 0.59 | 0.85 |
| P12821 | ACE | -0.058 | 0.60 | 0.85 |
| P22234 | PAICS | 0.144 | 0.60 | 0.85 |
| Q6UX04 | CWC27 | -0.081 | 0.60 | 0.85 |
| Q15904 | ATP6AP1 | -0.095 | 0.62 | 0.86 |
| Q14247 | CTTN | 0.147 | 0.62 | 0.86 |
| P04433 | IGKV3-11 | -0.161 | 0.62 | 0.86 |
| O43583 | DENR | -0.311 | 0.62 | 0.86 |
| Q9Y2H5 | PLEKHA6 | 0.068 | 0.62 | 0.86 |
| P0C0L4 | C4A | 0.095 | 0.62 | 0.86 |
| P20851 | C4BPB | 0.046 | 0.62 | 0.87 |
| P00740 | F9 | 0.040 | 0.63 | 0.87 |
| P35232 | PHB1 | -0.104 | 0.63 | 0.87 |
| P60842 | EIF4A1 | 0.312 | 0.63 | 0.87 |
| P50440 | GATM | -0.111 | 0.63 | 0.87 |
| P00751 | CFB | 0.061 | 0.64 | 0.87 |
| P11532 | DMD | 0.065 | 0.64 | 0.88 |
| Q09666 | AHNAK | 0.073 | 0.64 | 0.88 |
| P04040 | CAT | 0.136 | 0.64 | 0.88 |
| Q8NCW5 | NAXE | -0.058 | 0.65 | 0.88 |
| Q92945 | KHSRP | -0.281 | 0.66 | 0.88 |
| P09172 | DBH | 0.269 | 0.66 | 0.88 |
| P01776 | IGHV3-23 | -0.277 | 0.66 | 0.88 |
| P78417 | GSTO1 | 0.174 | 0.66 | 0.88 |
| P36952 | SERPINB5 | -0.081 | 0.66 | 0.88 |
| P14923 | JUP | 0.225 | 0.66 | 0.88 |
| Q9UQ35 | SRRM2 | -0.061 | 0.66 | 0.88 |
| Q9NQ79 | CRTAC1 | -0.156 | 0.67 | 0.88 |
| Q7Z794 | KRT77 | -0.153 | 0.67 | 0.89 |
| P35520 | CBS | 0.069 | 0.67 | 0.89 |
| P04220 | IGHM | 0.145 | 0.67 | 0.89 |
| P00918 | CA2 | 0.134 | 0.67 | 0.89 |
| Q16363 | LAMA4 | 0.055 | 0.68 | 0.90 |
| P08865 | RPSA | -0.225 | 0.69 | 0.90 |
| P12111 | COL6A3 | -0.052 | 0.69 | 0.90 |
| P06681 | C2 | 0.044 | 0.69 | 0.90 |
| Q9NYL9 | TMOD3 | 0.259 | 0.69 | 0.90 |
| P07996 | THBS1 | -0.505 | 0.69 | 0.90 |
| Q14353 | GAMT | 0.196 | 0.70 | 0.90 |
| Q07960 | ARHGAP1 | 0.060 | 0.71 | 0.92 |
| P01591 | JCHAIN | 0.109 | 0.71 | 0.92 |
| Q14203 | DCTN1 | 0.063 | 0.72 | 0.92 |
| P31948 | STIP1 | -0.059 | 0.72 | 0.92 |
| P32119 | PRDX2 | -0.101 | 0.72 | 0.92 |
| P01860 | IGHG3 | -0.264 | 0.72 | 0.92 |
| P30048 | PRDX3 | 0.086 | 0.72 | 0.92 |
| P48506 | GCLC | -0.081 | 0.73 | 0.93 |
| P08311 | CTSG | -0.042 | 0.73 | 0.93 |
| Q27J81 | INF2 | -0.232 | 0.73 | 0.93 |
| P08637 | FCGR3A | 0.061 | 0.74 | 0.93 |
| P49327 | FASN | -0.038 | 0.74 | 0.93 |
| Q9NP79 | VTA1 | 0.053 | 0.74 | 0.93 |
| O75368 | SH3BGRL | 0.174 | 0.75 | 0.93 |
| P24928 | POLR2A | -0.080 | 0.75 | 0.93 |
| P49913 | CAMP | -0.080 | 0.75 | 0.93 |
| Q99714 | HSD17B10 | -0.219 | 0.75 | 0.93 |
| P20618 | PSMB1 | 0.056 | 0.76 | 0.93 |
| P13727 | PRG2 | 0.055 | 0.76 | 0.93 |
| Q14012 | CAMK1 | -0.150 | 0.76 | 0.93 |
| P10643 | C7 | -0.036 | 0.76 | 0.93 |
| P35442 | THBS2 | -0.065 | 0.76 | 0.93 |
| O43866 | CD5L | 0.071 | 0.76 | 0.93 |
| P15924 | DSP | -0.044 | 0.77 | 0.94 |
| Q92835 | INPP5D | 0.055 | 0.77 | 0.94 |
| Q93099 | HGD | 0.030 | 0.77 | 0.94 |
| O15394 | NCAM2 | 0.052 | 0.77 | 0.94 |
| P05543 | SERPINA7 | -0.032 | 0.77 | 0.94 |
| Q04695 | KRT17 | 0.040 | 0.78 | 0.94 |
| P50552 | VASP | -0.083 | 0.78 | 0.94 |
| Q9BR76 | CORO1B | 0.102 | 0.78 | 0.94 |
| P60660 | MYL6 | 0.078 | 0.78 | 0.94 |
| P01781 | IGHV3-7 | -0.093 | 0.78 | 0.94 |
| Q8WXI7 | MUC16 | -0.102 | 0.78 | 0.94 |
| P60981 | DSTN | 0.039 | 0.79 | 0.94 |
| O75083 | WDR1 | -0.184 | 0.79 | 0.94 |
| Q92954 | PRG4 | -0.053 | 0.79 | 0.94 |
| Q9NZP8 | C1RL | -0.024 | 0.80 | 0.94 |
| P01034 | CST3 | -0.032 | 0.80 | 0.94 |
| P09417 | QDPR | 0.075 | 0.80 | 0.94 |
| O43488 | AKR7A2 | -0.098 | 0.80 | 0.94 |
| P54578 | USP14 | 0.061 | 0.81 | 0.94 |
| P12109 | COL6A1 | -0.063 | 0.81 | 0.94 |
| P62158 | CALM1 | 0.105 | 0.81 | 0.94 |
| P18206 | VCL | -0.040 | 0.82 | 0.95 |
| P20742 | PZP | 0.046 | 0.82 | 0.95 |
| P05155 | SERPING1 | -0.027 | 0.82 | 0.95 |
| P19013 | KRT4 | -0.134 | 0.82 | 0.95 |
| P13671 | C6 | 0.014 | 0.82 | 0.95 |
| P49736 | MCM2 | -0.055 | 0.82 | 0.95 |
| P61106 | RAB14 | -0.032 | 0.82 | 0.95 |
| O60716 | CTNND1 | 0.032 | 0.83 | 0.95 |
| P31040 | SDHA | -0.054 | 0.83 | 0.95 |
| P00746 | CFD | -0.029 | 0.83 | 0.95 |
| Q14789 | GOLGB1 | -0.046 | 0.83 | 0.95 |
| P31997 | CEACAM8 | -0.044 | 0.83 | 0.95 |
| P11277 | SPTB | 0.045 | 0.83 | 0.95 |
| Q12906 | ILF3 | 0.063 | 0.84 | 0.95 |
| P07333 | CSF1R | 0.031 | 0.85 | 0.95 |
| Q03252 | LMNB2 | 0.091 | 0.85 | 0.95 |
| Q9NQC3 | RTN4 | -0.022 | 0.85 | 0.95 |
| Q7L1Q6 | BZW1 | 0.028 | 0.85 | 0.95 |
| P78371 | CCT2 | -0.017 | 0.85 | 0.95 |
| P26927 | MST1 | 0.039 | 0.85 | 0.95 |
| O60240 | PLIN1 | 0.050 | 0.85 | 0.95 |
| P34913 | EPHX2 | 0.014 | 0.86 | 0.95 |
| O95678 | KRT75 | 0.078 | 0.86 | 0.95 |
| P15311 | EZR | -0.019 | 0.86 | 0.95 |
| O95831 | AIFM1 | 0.073 | 0.86 | 0.95 |
| P11766 | ADH5 | 0.019 | 0.87 | 0.95 |
| P36980 | CFHR2 | -0.032 | 0.87 | 0.95 |
| P41250 | GARS1 | -0.027 | 0.87 | 0.95 |
| Q96HR3 | MED30 | 0.017 | 0.87 | 0.95 |
| Q8WU39 | MZB1 | -0.056 | 0.87 | 0.95 |
| P30520 | ADSS2 | -0.077 | 0.87 | 0.96 |
| Q07954 | LRP1 | -0.019 | 0.88 | 0.96 |
| Q14240 | EIF4A2 | 0.062 | 0.88 | 0.96 |
| P00748 | F12 | -0.022 | 0.90 | 0.97 |
| P50395 | GDI2 | 0.059 | 0.90 | 0.97 |
| P23470 | PTPRG | 0.023 | 0.90 | 0.97 |
| P50454 | SERPINH1 | -0.015 | 0.90 | 0.97 |
| Q99683 | MAP3K5 | 0.013 | 0.90 | 0.97 |
| P82979 | SARNP | 0.022 | 0.90 | 0.97 |
| P12830 | CDH1 | 0.028 | 0.91 | 0.97 |
| P02745 | C1QA | 0.034 | 0.91 | 0.98 |
| Q14697 | GANAB | 0.023 | 0.92 | 0.98 |
| P06727 | APOA4 | -0.016 | 0.92 | 0.98 |
| P11717 | IGF2R | -0.023 | 0.92 | 0.98 |
| O75144 | ICOSLG | -0.028 | 0.92 | 0.98 |
| Q9Y2V2 | CARHSP1 | 0.007 | 0.92 | 0.98 |
| P42765 | ACAA2 | 0.013 | 0.92 | 0.98 |
| P10809 | HSPD1 | -0.045 | 0.93 | 0.98 |
| Q14624 | ITIH4 | 0.008 | 0.93 | 0.98 |
| P04075 | ALDOA | 0.024 | 0.93 | 0.98 |
| P09972 | ALDOC | 0.025 | 0.93 | 0.98 |
| P06331 | IGHV4-34 | -0.034 | 0.94 | 0.98 |
| P35527 | KRT9 | -0.028 | 0.94 | 0.99 |
| P15144 | ANPEP | 0.006 | 0.94 | 0.99 |
| P07339 | CTSD | 0.043 | 0.95 | 0.99 |
| Q6P179 | ERAP2 | 0.024 | 0.95 | 0.99 |
| P11021 | HSPA5 | -0.008 | 0.96 | 0.99 |
| P04792 | HSPB1 | 0.035 | 0.96 | 0.99 |
| O75390 | CS | 0.005 | 0.96 | 0.99 |
| Q9H4M9 | EHD1 | -0.010 | 0.96 | 0.99 |
| P08571 | CD14 | -0.008 | 0.97 | 0.99 |
| Q6IBS0 | TWF2 | 0.008 | 0.97 | 0.99 |
| P05546 | SERPIND1 | -0.003 | 0.97 | 0.99 |
| P04275 | VWF | -0.006 | 0.97 | 0.99 |
| P01780 | IGHV3-7 | -0.018 | 0.97 | 0.99 |
| P17980 | PSMC3 | -0.014 | 0.98 | 1.00 |
| Q08209 | PPP3CA | -0.007 | 0.98 | 1.00 |
| P47897 | QARS1 | -0.003 | 0.98 | 1.00 |
| Q12913 | PTPRJ | -0.009 | 0.98 | 1.00 |
| Q9UNN8 | PROCR | 0.003 | 0.98 | 1.00 |
| P35579 | MYH9 | 0.005 | 0.99 | 1.00 |
| P50991 | CCT4 | 0.002 | 0.99 | 1.00 |
| P01621 | IGKV3-20 | -0.004 | 0.99 | 1.00 |
| Q92820 | GGH | 0.002 | 0.99 | 1.00 |
| Q86VB7 | CD163 | 0.002 | 0.99 | 1.00 |
| Q9UKV8 | AGO2 | 0.002 | 0.99 | 1.00 |
| P03951 | F11 | 0.001 | 1.00 | 1.00 |

**Supplementary Table S4**: Nested diagnostic model composition and predictive accuracy based on discriminatory biomarkers in cervico-vaginal fluid debris using the mean decrease accuracy metric of the random forest model

|  | SLP1 | FBLN1 | DSG3 | AIBG | ANXA1 | TPI1 | AUC (95% CI) | AIC | SEN ,%(95%CI) | SPE, %(95%CI) | PPV^a^ ,%(95%CI) | NPV^a^ ,%(95%CI) |
| --- | --- | --- | --- | --- | --- | --- | --- | --- | --- | --- | --- | --- |
| Model 1 | X |  |  |  |  |  | 0.77(0.67-0.86) | 130 | 62 (48-76) | 85(76-94) | 30 (12-49) | 96 (92-100) |
| Model 2 | X | X |  |  |  |  | 0.81(0.72-0.90) | 118 | 67 (53-80) | 87(78-95) | 33 (13-53) | 96 (92-100) |
| Model 3 | X | X | X |  |  |  | 0.85(0.78-0.93) | 111 | 73 (60-86) | 85 (76-94) | 33 (14-52) | 97 (93-100) |
| Model 4 | X | X | X | X |  |  | 0.90(0.85-0.96) | 96 | 78 (66-90) | 90 (82-97) | 42 (20-64) | 98 (95-100) |
| Model 5 | X | X | X | X | X |  | 0.90(0.85-0.96) | 98 | 78 (66-90) | 88 (80-96) | 38 (17-59) | 98 (95-100) |
| Model 6 | X | X | X | X | X | X | 0.91(0.86-0.97) | 98 | 80 (68-92) | 90 (82-98) | 42 (20-64) | 98 (95-100) |

The order in which the classifiers were entered in the model was determined by their ranking in the mean decrease accuracy metric of random forest. All models were adjusted for age and BMI as continuous variables. AUC=area under the receiver operator characteristic curve. AIC=Akaike information criterion. SEN=sensitivity. SPE=specificity. PPV=positive predictive value. NPV=negative predictive value. CI=Confidence interval.The parsimonious model of cervico-vaginal fluid debris/pellet proteins for endometrial cancer detection comprised SLP1, FBLN1, DSG3 and AIBG.

**a: assumed disease prevalence of 9%.**

**Supplementary Table S5:** Spearman’s rank correlation analyses of overlapping proteins in cervicovaginal fluid and plasma samples.

| **Uniprot Accession** | **Uniprot ID** | **Spearman rho** | **Lower CI** | **Upper CI** | **p-value** |  |
| --- | --- | --- | --- | --- | --- | --- |
| O43866 | CD5L_HUMAN | 0.413 | 0.240 | 0.562 | <0.0001 |  |
| P02748 | CO9_HUMAN | 0.385 | 0.215 | 0.533 | <0.0001 |  |
| P08519 | APOA_HUMAN | 0.380 | 0.187 | 0.541 | <0.0001 |  |
| P01023 | A2MG_HUMAN | 0.309 | 0.116 | 0.469 | 0.0012 |  |
| P04003 | C4BPA_HUMAN | 0.281 | 0.096 | 0.451 | 0.0031 |  |
| P01024 | CO3_HUMAN | 0.262 | 0.071 | 0.431 | 0.0060 |  |
| Q01518 | CAP1_HUMAN | 0.237 | 0.050 | 0.393 | 0.013 |  |
| P28066 | PSA5_HUMAN | 0.230 | 0.032 | 0.423 | 0.016 |  |
| O43707 | ACTN4_HUMAN | 0.225 | 0.039 | 0.384 | 0.018 |  |
| P00739 | HPTR_HUMAN | 0.216 | 0.017 | 0.392 | 0.024 |  |
| P60660 | MYL6_HUMAN | 0.215 | 0.020 | 0.390 | 0.025 |  |
| P07357 | CO8A_HUMAN | 0.211 | 0.038 | 0.383 | 0.028 |  |
| P04792 | HSPB1_HUMAN | 0.209 | 0.018 | 0.381 | 0.029 |  |
| P02750 | A2GL_HUMAN | 0.209 | 0.006 | 0.390 | 0.029 |  |
| P14625 | ENPL_HUMAN | -0.209 | -0.375 | -0.024 | 0.029 |  |
| P36952 | SPB5_HUMAN | 0.208 | 0.005 | 0.391 | 0.030 |  |
| P01876 | IGHA1_HUMAN | 0.199 | 0.026 | 0.367 | 0.038 |  |
| P01591 | IGJ_HUMAN | 0.198 | 0.015 | 0.376 | 0.039 |  |
| P01621 | KV320_HUMAN | 0.198 | 0.029 | 0.374 | 0.039 |  |
| P02749 | APOH_HUMAN | 0.194 | 0.012 | 0.367 | 0.043 |  |
| P20810 | ICAL_HUMAN | -0.188 | -0.340 | -0.021 | 0.050 |  |
| P01031 | CO5_HUMAN | 0.184 | -0.004 | 0.362 | 0.055 |  |
| P80748 | LV321_HUMAN | 0.184 | -0.010 | 0.365 | 0.056 |  |
| Q99460 | PSMD1_HUMAN | -0.182 | -0.362 | 0.009 | 0.058 |  |
| P02763 | A1AG1_HUMAN | 0.182 | -0.021 | 0.374 | 0.058 |  |
| P08670 | VIME_HUMAN | -0.182 | -0.350 | 0.007 | 0.058 |  |
| P02675 | FIBB_HUMAN | 0.178 | -0.014 | 0.358 | 0.064 |  |
| Q06033 | ITIH3_HUMAN | 0.178 | -0.015 | 0.365 | 0.064 |  |
| P0C0L5 | CO4B_HUMAN | -0.175 | -0.353 | 0.016 | 0.068 |  |
| P01042 | KNG1_HUMAN | -0.174 | -0.346 | 0.012 | 0.070 |  |
| P00748 | FA12_HUMAN | 0.170 | -0.008 | 0.342 | 0.076 |  |
| P06733 | ENOA_HUMAN | 0.168 | -0.013 | 0.343 | 0.080 |  |
| Q9Y490 | TLN1_HUMAN | 0.167 | -0.024 | 0.336 | 0.082 |  |
| P01011 | AACT_HUMAN | 0.166 | -0.035 | 0.356 | 0.084 |  |
| P08603 | CFAH_HUMAN | 0.166 | -0.017 | 0.336 | 0.085 |  |
| P07237 | PDIA1_HUMAN | 0.166 | -0.021 | 0.336 | 0.085 |  |
| P00751 | CFAB_HUMAN | 0.164 | -0.042 | 0.334 | 0.088 |  |
| P06702 | S10A9_HUMAN | -0.164 | -0.335 | 0.015 | 0.088 |  |
| P13647 | K2C5_HUMAN | -0.164 | -0.365 | 0.040 | 0.089 |  |
| P02679 | FIBG_HUMAN | 0.158 | -0.028 | 0.337 | 0.10 |  |
| P21333 | FLNA_HUMAN | 0.153 | -0.034 | 0.340 | 0.11 |  |
| P25311 | ZA2G_HUMAN | -0.153 | -0.325 | 0.048 | 0.11 |  |
| P07360 | CO8G_HUMAN | 0.153 | -0.022 | 0.338 | 0.11 |  |
| P50395 | GDIB_HUMAN | -0.151 | -0.333 | 0.040 | 0.12 |  |
| P00450 | CERU_HUMAN | -0.149 | -0.332 | 0.032 | 0.12 |  |
| P02656 | APOC3_HUMAN | 0.146 | -0.039 | 0.316 | 0.13 |  |
| P13645 | K1C10_HUMAN | 0.145 | -0.075 | 0.351 | 0.13 |  |
| P02042 | HBD_HUMAN | -0.142 | -0.324 | 0.054 | 0.14 |  |
| P01781 | HV307_HUMAN | 0.141 | -0.038 | 0.308 | 0.14 |  |
| P69905 | HBA_HUMAN | -0.138 | -0.302 | 0.028 | 0.15 |  |
| P30050 | RL12_HUMAN | 0.133 | -0.056 | 0.333 | 0.17 |  |
| P02649 | APOE_HUMAN | 0.132 | -0.070 | 0.311 | 0.17 |  |
| P05156 | CFAI_HUMAN | 0.131 | -0.070 | 0.312 | 0.18 |  |
| P23142 | FBLN1_HUMAN | 0.130 | -0.056 | 0.335 | 0.18 |  |
| P02751 | FINC_HUMAN | 0.124 | -0.123 | 0.208 | 0.18 |  |
| P05109 | S10A8_HUMAN | -0.129 | -0.292 | 0.061 | 0.18 |  |
| P13671 | CO6_HUMAN | 0.128 | -0.052 | 0.320 | 0.18 |  |
| P12429 | ANXA3_HUMAN | -0.127 | -0.307 | 0.044 | 0.19 |  |
| Q16610 | ECM1_HUMAN | 0.127 | -0.060 | 0.312 | 0.19 |  |
| P22792 | CPN2_HUMAN | 0.124 | -0.058 | 0.304 | 0.20 |  |
| P01009 | A1AT_HUMAN | 0.124 | -0.071 | 0.314 | 0.20 |  |
| P17936 | IBP3_HUMAN | 0.123 | -0.081 | 0.311 | 0.20 |  |
| P27824 | CALX_HUMAN | -0.123 | -0.307 | 0.067 | 0.20 |  |
| P04040 | CATA_HUMAN | -0.121 | -0.304 | 0.073 | 0.21 |  |
| P68871 | HBB_HUMAN | -0.119 | -0.307 | 0.064 | 0.22 |  |
| P00915 | CAH1_HUMAN | -0.119 | -0.303 | 0.094 | 0.22 |  |
| P02743 | SAMP_HUMAN | 0.118 | -0.075 | 0.307 | 0.22 |  |
| P29622 | KAIN_HUMAN | -0.117 | -0.290 | 0.093 | 0.23 |  |
| P30048 | PRDX3_HUMAN | -0.115 | -0.293 | 0.076 | 0.24 |  |
| P03952 | KLKB1_HUMAN | -0.114 | -0.290 | 0.071 | 0.24 |  |
| P04004 | VTNC_HUMAN | -0.113 | -0.292 | 0.063 | 0.24 |  |
| P18206 | VINC_HUMAN | -0.113 | -0.296 | 0.070 | 0.24 |  |
| P07195 | LDHB_HUMAN | 0.112 | -0.076 | 0.303 | 0.25 |  |
| O75874 | IDHC_HUMAN | 0.111 | -0.097 | 0.309 | 0.25 |  |
| P15924 | DESP_HUMAN | -0.110 | -0.275 | 0.072 | 0.25 |  |
| P04433 | KV311_HUMAN | 0.110 | -0.067 | 0.292 | 0.25 |  |
| P01857 | IGHG1_HUMAN | 0.108 | -0.075 | 0.276 | 0.26 |  |
| Q9Y6R7 | FCGBP_HUMAN | 0.108 | -0.072 | 0.292 | 0.27 |  |
| P01034 | CYTC_HUMAN | 0.107 | -0.078 | 0.299 | 0.27 |  |
| P01859 | IGHG2_HUMAN | 0.106 | -0.068 | 0.278 | 0.27 |  |
| P20618 | PSB1_HUMAN | 0.103 | -0.110 | 0.300 | 0.29 |  |
| P0C0L4 | CO4A_HUMAN | -0.102 | -0.291 | 0.082 | 0.29 |  |
| O60664 | PLIN3_HUMAN | 0.102 | -0.068 | 0.285 | 0.29 |  |
| P04196 | HRG_HUMAN | -0.100 | -0.289 | 0.112 | 0.30 |  |
| P50991 | TCPD_HUMAN | -0.100 | -0.285 | 0.090 | 0.30 |  |
| P25705 | ATPA_HUMAN | -0.097 | -0.279 | 0.106 | 0.32 |  |
| P06576 | ATPB_HUMAN | 0.095 | -0.092 | 0.291 | 0.32 |  |
| O14818 | PSA7_HUMAN | 0.091 | -0.091 | 0.267 | 0.33 |  |
| P52566 | GDIR2_HUMAN | -0.093 | -0.284 | 0.090 | 0.33 |  |
| O75369 | FLNB_HUMAN | -0.092 | -0.282 | 0.101 | 0.34 |  |
| P11279 | LAMP1_HUMAN | 0.092 | -0.093 | 0.295 | 0.34 |  |
| P02671 | FIBA_HUMAN | -0.877 | -0.913 | -0.827 | 0.34 |  |
| P23396 | RS3_HUMAN | -0.089 | -0.277 | 0.104 | 0.36 |  |
| P30041 | PRDX6_HUMAN | 0.089 | -0.100 | 0.267 | 0.36 |  |
| P06331 | HV434_HUMAN | 0.089 | -0.116 | 0.267 | 0.36 |  |
| P10643 | CO7_HUMAN | 0.088 | -0.106 | 0.270 | 0.36 |  |
| P36955 | PEDF_HUMAN | 0.088 | -0.123 | 0.271 | 0.36 |  |
| P07225 | PROS_HUMAN | 0.087 | -0.119 | 0.284 | 0.37 |  |
| O14791 | APOL1_HUMAN | 0.087 | -0.112 | 0.257 | 0.37 |  |
| P26038 | MOES_HUMAN | 0.087 | -0.103 | 0.262 | 0.37 |  |
| P49327 | FAS_HUMAN | 0.086 | -0.122 | 0.283 | 0.37 |  |
| P02787 | TRFE_HUMAN | 0.085 | -0.117 | 0.262 | 0.38 |  |
| P06727 | APOA4_HUMAN | 0.085 | -0.114 | 0.276 | 0.38 |  |
| Q08380 | LG3BP_HUMAN | -0.081 | -0.258 | 0.101 | 0.39 |  |
| P05543 | THBG_HUMAN | 0.083 | -0.124 | 0.271 | 0.39 |  |
| P19013 | K2C4_HUMAN | 0.081 | -0.098 | 0.275 | 0.40 |  |
| P00338 | LDHA_HUMAN | 0.080 | -0.116 | 0.261 | 0.41 |  |
| P08107 | HS71A_HUMAN | 0.080 | -0.112 | 0.275 | 0.41 |  |
| Q13263 | TIF1B_HUMAN | -0.080 | -0.267 | 0.117 | 0.41 |  |
| Q96KP4 | CNDP2_HUMAN | 0.078 | -0.124 | 0.262 | 0.42 |  |
| P02655 | APOC2_HUMAN | 0.078 | -0.104 | 0.260 | 0.42 |  |
| P00734 | THRB_HUMAN | -0.077 | -0.272 | 0.123 | 0.43 |  |
| P09972 | ALDOC_HUMAN | 0.077 | -0.120 | 0.271 | 0.43 |  |
| P00738 | HPT_HUMAN | -0.070 | -0.248 | 0.112 | 0.43 |  |
| P02790 | HEMO_HUMAN | 0.075 | -0.114 | 0.257 | 0.44 |  |
| P11413 | G6PD_HUMAN | 0.075 | -0.128 | 0.286 | 0.44 |  |
| P55786 | PSA_HUMAN | -0.075 | -0.258 | 0.114 | 0.44 |  |
| P19367 | HXK1_HUMAN | -0.075 | -0.273 | 0.134 | 0.44 |  |
| P61769 | B2MG_HUMAN | 0.075 | -0.123 | 0.276 | 0.44 |  |
| P68036 | UB2L3_HUMAN | 0.073 | -0.122 | 0.259 | 0.45 |  |
| P01860 | IGHG3_HUMAN | 0.073 | -0.125 | 0.263 | 0.45 |  |
| P49368 | TCPG_HUMAN | 0.073 | -0.106 | 0.260 | 0.45 |  |
| P49913 | CAMP_HUMAN | 0.072 | -0.135 | 0.259 | 0.46 |  |
| P35527 | K1C9_HUMAN | -0.071 | -0.253 | 0.113 | 0.46 |  |
| P22234 | PUR6_HUMAN | 0.071 | -0.120 | 0.248 | 0.47 |  |
| P02753 | RET4_HUMAN | 0.070 | -0.131 | 0.263 | 0.47 |  |
| P30101 | PDIA3_HUMAN | -0.069 | -0.260 | 0.119 | 0.47 |  |
| P01714 | LV319_HUMAN | 0.068 | -0.119 | 0.255 | 0.48 |  |
| P02765 | FETUA_HUMAN | 0.067 | -0.129 | 0.252 | 0.49 |  |
| Q09666 | AHNK_HUMAN | -0.067 | -0.255 | 0.118 | 0.49 |  |
| P09651 | ROA1_HUMAN | 0.064 | -0.122 | 0.275 | 0.51 |  |
| O75882 | ATRN_HUMAN | 0.064 | -0.127 | 0.242 | 0.51 |  |
| Q92820 | GGH_HUMAN | 0.062 | -0.147 | 0.239 | 0.52 |  |
| P31997 | CEAM8_HUMAN | -0.061 | -0.228 | 0.124 | 0.53 |  |
| P37802 | TAGL2_HUMAN | 0.061 | -0.139 | 0.242 | 0.53 |  |
| P04264 | K2C1_HUMAN | -0.059 | -0.248 | 0.114 | 0.54 |  |
| P20930 | FILA_HUMAN | -0.056 | -0.251 | 0.152 | 0.56 |  |
| P19827 | ITIH1_HUMAN | -0.053 | -0.224 | 0.129 | 0.59 |  |
| P07355 | ANXA2_HUMAN | -0.050 | -0.236 | 0.149 | 0.60 |  |
| Q96PD5 | PGRP2_HUMAN | -0.050 | -0.228 | 0.124 | 0.60 |  |
| Q7Z794 | K2C1B_HUMAN | -0.049 | -0.234 | 0.134 | 0.61 |  |
| P51884 | LUM_HUMAN | 0.048 | -0.142 | 0.225 | 0.62 |  |
| Q13228 | SBP1_HUMAN | -0.046 | -0.228 | 0.144 | 0.64 |  |
| P19971 | TYPH_HUMAN | -0.045 | -0.247 | 0.140 | 0.64 |  |
| Q14974 | IMB1_HUMAN | 0.045 | -0.135 | 0.230 | 0.64 |  |
| P04220 | IGHM_HUMAN | 0.044 | -0.167 | 0.250 | 0.65 |  |
| P02647 | APOA1_HUMAN | -0.043 | -0.206 | 0.138 | 0.65 |  |
| P19823 | ITIH2_HUMAN | -0.043 | -0.220 | 0.141 | 0.66 |  |
| P35908 | K22E_HUMAN | 0.043 | -0.152 | 0.239 | 0.66 |  |
| P10909 | CLUS_HUMAN | -0.043 | -0.219 | 0.139 | 0.66 |  |
| P60174 | TPIS_HUMAN | -0.042 | -0.228 | 0.158 | 0.66 |  |
| P07339 | CATD_HUMAN | -0.037 | -0.211 | 0.155 | 0.70 |  |
| P36222 | CH3L1_HUMAN | -0.037 | -0.214 | 0.172 | 0.71 |  |
| P12830 | CADH1_HUMAN | 0.036 | -0.148 | 0.207 | 0.71 |  |
| P19652 | A1AG2_HUMAN | 0.035 | -0.147 | 0.222 | 0.71 |  |
| O43175 | SERA_HUMAN | -0.035 | -0.209 | 0.159 | 0.71 |  |
| P55058 | PLTP_HUMAN | -0.035 | -0.226 | 0.154 | 0.72 |  |
| P04406 | G3P_HUMAN | -0.035 | -0.227 | 0.167 | 0.72 |  |
| P01019 | ANGT_HUMAN | -0.034 | -0.211 | 0.129 | 0.72 |  |
| P43490 | NAMPT_HUMAN | -0.034 | -0.227 | 0.162 | 0.72 |  |
| P01834 | IGKC_HUMAN | 0.034 | -0.144 | 0.207 | 0.72 |  |
| P00747 | PLMN_HUMAN | -0.034 | -0.230 | 0.146 | 0.73 |  |
| P09871 | C1S_HUMAN | 0.033 | -0.152 | 0.202 | 0.73 |  |
| P01008 | ANT3_HUMAN | 0.032 | -0.152 | 0.202 | 0.74 |  |
| P27169 | PON1_HUMAN | 0.032 | -0.145 | 0.207 | 0.74 |  |
| P02747 | C1QC_HUMAN | -0.031 | -0.232 | 0.170 | 0.75 |  |
| Q14624 | ITIH4_HUMAN | 0.031 | -0.148 | 0.226 | 0.75 |  |
| P11021 | BIP_HUMAN | -0.031 | -0.224 | 0.162 | 0.75 |  |
| P04217 | A1BG_HUMAN | 0.031 | -0.152 | 0.211 | 0.75 |  |
| P60842 | IF4A1_HUMAN | -0.030 | -0.213 | 0.151 | 0.75 |  |
| P08697 | A2AP_HUMAN | 0.030 | -0.166 | 0.228 | 0.76 |  |
| P06753 | TPM3_HUMAN | -0.029 | -0.214 | 0.164 | 0.76 |  |
| P02760 | AMBP_HUMAN | -0.029 | -0.217 | 0.164 | 0.76 |  |
| P12111 | CO6A3_HUMAN | 0.025 | -0.180 | 0.224 | 0.80 |  |
| P23528 | COF1_HUMAN | 0.024 | -0.160 | 0.215 | 0.80 |  |
| P01877 | IGHA2_HUMAN | 0.024 | -0.161 | 0.198 | 0.81 |  |
| P02652 | APOA2_HUMAN | 0.023 | -0.047 | 0.268 | 0.81 |  |
| P43251 | BTD_HUMAN | -0.023 | -0.211 | 0.167 | 0.81 |  |
| P06681 | CO2_HUMAN | 0.023 | -0.151 | 0.222 | 0.81 |  |
| P12955 | PEPD_HUMAN | 0.023 | -0.175 | 0.225 | 0.81 |  |
| P00918 | CAH2_HUMAN | -0.023 | -0.208 | 0.176 | 0.82 |  |
| P08865 | RSSA_HUMAN | 0.022 | -0.166 | 0.218 | 0.82 |  |
| P34932 | HSP74_HUMAN | -0.022 | -0.216 | 0.152 | 0.82 |  |
| P02654 | APOC1_HUMAN | 0.020 | -0.171 | 0.209 | 0.84 |  |
| P05546 | HEP2_HUMAN | -0.019 | -0.186 | 0.169 | 0.84 |  |
| P02774 | VTDB_HUMAN | -0.019 | -0.190 | 0.141 | 0.84 |  |
| P08185 | CBG_HUMAN | 0.019 | -0.169 | 0.198 | 0.85 |  |
| P07108 | ACBP_HUMAN | 0.019 | -0.158 | 0.199 | 0.85 |  |
| P01780 | HV307_HUMAN | -0.019 | -0.198 | 0.170 | 0.85 |  |
| P01871 | IGHM_HUMAN | -0.017 | -0.197 | 0.164 | 0.85 |  |
| P13473 | LAMP2_HUMAN | -0.018 | -0.213 | 0.177 | 0.86 |  |
| P00441 | SODC_HUMAN | -0.018 | -0.210 | 0.165 | 0.86 |  |
| P08311 | CATG_HUMAN | 0.016 | -0.173 | 0.193 | 0.87 |  |
| P25787 | PSA2_HUMAN | 0.014 | -0.174 | 0.211 | 0.89 |  |
| P62158 | CALM2_HUMAN | -0.012 | -0.184 | 0.185 | 0.90 |  |
| O15143 | ARC1B_HUMAN | 0.011 | -0.172 | 0.194 | 0.91 |  |
| P04114 | APOB_HUMAN | 0.011 | -0.184 | 0.181 | 0.91 |  |
| P01861 | IGHG4_HUMAN | 0.010 | -0.185 | 0.195 | 0.91 |  |
| P02768 | ALBU_HUMAN | 0.010 | -0.165 | 0.194 | 0.92 |  |
| P62263 | RS14_HUMAN | 0.010 | -0.184 | 0.223 | 0.92 |  |
| P05164 | PERM_HUMAN | 0.009 | -0.186 | 0.185 | 0.92 |  |
| Q9Y2V2 | CHSP1_HUMAN | -0.009 | -0.195 | 0.176 | 0.92 |  |
| P06737 | PYGL_HUMAN | 0.009 | -0.188 | 0.213 | 0.93 |  |
| P35579 | MYH9_HUMAN | -0.008 | -0.200 | 0.163 | 0.94 |  |
| P54108 | CRIS3_HUMAN | 0.007 | -0.199 | 0.198 | 0.94 |  |
| P04075 | ALDOA_HUMAN | 0.006 | -0.168 | 0.182 | 0.95 |  |
| P05090 | APOD_HUMAN | 0.005 | -0.185 | 0.191 | 0.96 |  |
| P16070 | CD44_HUMAN | -0.005 | -0.192 | 0.186 | 0.96 |  |
| O75368 | SH3L1_HUMAN | -0.005 | -0.196 | 0.192 | 0.96 |  |
| P02746 | C1QB_HUMAN | 0.004 | -0.198 | 0.184 | 0.97 |  |
| P32119 | PRDX2_HUMAN | 0.004 | -0.168 | 0.187 | 0.97 |  |
| P61106 | RAB14_HUMAN | -0.004 | -0.188 | 0.191 | 0.97 |  |
| P02766 | TTHY_HUMAN | -0.003 | -0.205 | 0.185 | 0.97 |  |
| O75347 | TBCA_HUMAN | 0.001 | -0.192 | 0.204 | 0.99 |  |
| P15311 | EZRI_HUMAN | 0.001 | -0.177 | 0.171 | 0.99 |  |
| P01717 | LV325_HUMAN | 0.000 | -0.188 | 0.193 | 1.00 |  |
|  |  |  |  |  |  |  |
|  |  |  |  |  |  |  |

**Supplementary Figure S4:** (A) Box plots showing the permutation importance of the cervico-vaginal cell pellet derived proteins confirmed by the Boruta algorithm to be important. (B) Cumulative AUC for the Boruta-identified proteins based on multiple forward stepwise logistic regression. (C) Gene ontological analysis of the unique Boruta identified biomarkers using the webserver WebGestalt and showing the biological (red), cellular (blue) and molecular (green) functions.


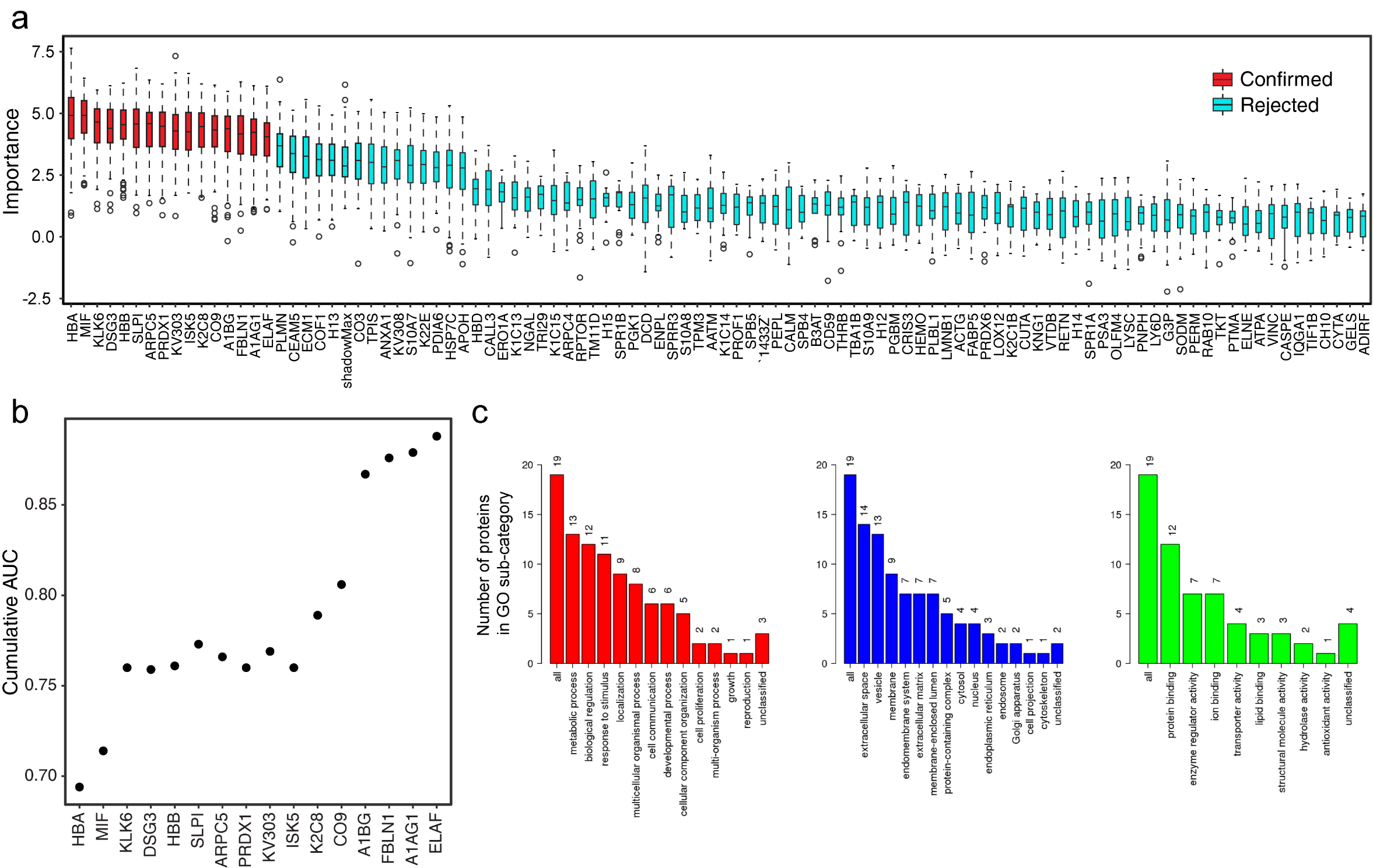


**Supplementary Table S6:** Nested diagnostic model composition and predictive accuracy based on the top discriminatory biomarkers in cervico-vaginal fluid supernatant for the detection of stage I endometrial cancer using the mean decrease accuracy metric of the random Forest model

|  | HPT | LY6D | C5 | A2MG | ITIH4 | FGA | FGB | AUC (95% CI) | AIC | SEN, %(95%CI) | SPE, %(95%CI) | PPV^a^, %(95%CI) | NPV^a^, %(95%CI) |
| --- | --- | --- | --- | --- | --- | --- | --- | --- | --- | --- | --- | --- | --- |
| Model 1 | X |  |  |  |  |  |  | 0.89(0.83-0.95) | 99 | 66 (51-80) | 88 (79-96) | 35 (14-56) | 96 (92-100) |
| Model 2 | X | X |  |  |  |  |  | 0.91(0.85-0.96) | 88 | 83 (71-94) | 86 (78-95) | 38 (18-57) | 98 (95-100) |
| Model 3 | X | X | X |  |  |  |  | 0.92(0.87-0.97) | 84 | 85 (75-96) | 88 (80-96) | 41 (20-61) | 98 (95-100) |
| Model 4 | X | X | X | X |  |  |  | 0.93(0.88-0.97) | 85 | 83 (71-94) | 88 (80-96) | 41 (20-61) | 98 (95-100) |
| Model 5 | X | X | X | X | X |  |  | 0.93(0.89-0.98) | 86 | 85 (75-96) | 89 (82-97) | 43 (22-64) | 98 (95-100) |
| Model 6 | X | X | X | X | X | X |  | 0.93(0.89-0.98) | 87 | 80 (68-93) | 88 (80-96) | 38 (17-59) | 98 (95-100) |
| Model 7 | X | X | X | X | X | X | X | 0.93(0.89-0.98) | 88 | 83 (71-94) | 88 (80-96) | 41 (20-61) | 98 (95-100) |

The order in which the classifiers were entered in the model was determined by their ranking in the mean decrease accuracy metric of random forest. All models were adjusted for age and BMI as continuous variables. AUC=area under the receiver operator characteristic curve. AIC=Akaike information criterion. SEN=sensitivity. SPE=specificity. PPV=positive predictive value. NPV=negative predictive value. CI=Confidence interval. A 3-marker panel of cervico-vaginal fluid proteins comprising of HPT, LY6D and C5 predicted stage I endometrial cancers with AUC 0.92.

**a: assumed disease prevalence of 9%.**

**Supplementary Table S7**: Nested diagnostic model composition and predictive accuracy based on the top discriminatory biomarkers in plasma for the detection of stage I endometrial cancer using the mean decrease accuracy metric of the random forest model.

|  | CNDPI | CDC5L | APOD | PRDX6 | IGFALS | ATP5 | C3 | AUC (95% CI) | AIC | SEN,%(95%CI) | SPE,%(95%CI) | PPV^a^ ,%(95%CI) | NPV^a^ ,%(95%CI) |
| --- | --- | --- | --- | --- | --- | --- | --- | --- | --- | --- | --- | --- | --- |
| Model 1 | X |  |  |  |  |  |  | 0.83(0.75-0.91) | 122 | 63 (49-77) | 86 (77-94) | 32 (12-51) | 96 (92-100) |
| Model 2 | X | X |  |  |  |  |  | 0.84(0.77-0.92) | 120 | 70 (56-83) | 88 (79-96) | 35 (14-56) | 97 (93-100) |
| Model 3 | X | X | X |  |  |  |  | 0.86(0.79-0.93) | 115 | 72 (59-85) | 83 (74-92) | 31 (13-49) | 97 (93-100) |
| Model 4 | X | X | X | X |  |  |  | 0.88(0.82-0.95) | 111 | 76 (64-88) | 84 (75-93) | 32 (14-50) | 97 (93-100) |
| Model 5 | X | X | X | X | X |  |  | 0.89(0.82-0.95) | 113 | 80 (69-92) | 83 (74-92) | 31 (13-49) | 98 (95-100) |
| Model 6 | X | X | X | X | X | X |  | 0.89(0.83-0.96) | 114 | 78 (66-90) | 86 (77-94) | 35 (15-54) | 98 (95-100) |
| Model 7 | X | X | X | X | X | X | X | 0.89(0.83-0.96) | 116 | 76 (64-88) | 84 (75-93) | 32 (14-50) | 97 (93-100) |

The order in which the classifiers were entered in the model was determined by their ranking in the mean decrease accuracy metric of random forest. All models were adjusted for age and BMI as continuous variables. AUC=area under the receiver operator characteristic curve. AIC=Akaike information criterion. SEN=sensitivity. SPE=specificity. PPV=positive predictive value. NPV=negative predictive value.CI=Confidence interval. A 4-marker panel of plasma proteins incorporating CNDP1, CDC5L. APOD and PRDX6 predicted stage 1 endometrial cancer with an AUC of 0.88 (0.82-0.95).

**a: assumed disease prevalence of 9%.**
